# A checklist of vascular plants of the W National Park in Burkina Faso, including the adjacent hunting zones of Tapoa-Djerma and Kondio

**DOI:** 10.3897/BDJ.8.e54205

**Published:** 2020-07-03

**Authors:** Blandine M. I. Nacoulma, Marco Schmidt, Karen Hahn, Adjima Thiombiano

**Affiliations:** 1 Université Joseph Ki-Zerbo, Ouagadougou, Burkina Faso Université Joseph Ki-Zerbo Ouagadougou Burkina Faso; 2 Senckenberg Biodiversity and Climate Research Centre, Frankfurt am Main, Germany Senckenberg Biodiversity and Climate Research Centre Frankfurt am Main Germany; 3 Palmengarten, Frankfurt am Main, Germany Palmengarten Frankfurt am Main Germany; 4 Goethe University, Frankfurt am Main, Germany Goethe University Frankfurt am Main Germany

**Keywords:** plant diversity, new species, flora of Burkina Faso, protected areas, WAP Complex, West Africa

## Abstract

**Background:**

The W National Park and its two hunting zones represent a unique ecosystem in Burkina Faso for biodiversity conservation. This study aims at providing a detailed view of the current state of the floristic diversity as a baseline for future projects aiming at protecting and managing its resources. We combined intensive inventories and distribution records from vegetation plots, photo records and herbarium collections.

**New information:**

This is the first comprehensive checklist of vascular plants of the Burkina Faso part of the transborder W National Park. With 721 documented species including 19 species new to Burkina Faso, the Burkina Faso part of the W National Park is, so far, the nature reserve with most plant species in Burkina Faso. To a large extent, this may be assigned to its high habitat diversity and moderate degree of disturbance, but also to a relatively large area within an even larger complex of neighbouring protected areas, as well as comprehensive spatial, ecological and seasonal sampling efforts. However, as a World Heritage Site and regarding the current general context of insecurity, the W National Park and the entire WAP Complex require more international attention in order to ensure its conservation.

## Introduction

In times of growing land use pressure in West Africa, protected areas play an increasingly important role in the conservation of wildlife as well as plant species (Clerici et al. 2007, Zoungrana et al. 2018). The W-Arly-Pendjari Complex (WAP Complex) is the largest transborder complex of West African nature reserves shared amongst the three countries Benin, Burkina Faso and Niger and a World Heritage Site of the UNESCO (UNESCO World Heritage Committee 2017). The three partner countries seek to further optimise and formalise joint management systems for the regional park, which are already partially in place (Miller et al. 2015). Remote sensing studies (Schulte to Bühne et al. 2017) showed vegetation cover within the protected areas to remain relatively stable, while decreasing in the surroundings between 2000 and 2013. Due to agricultural expansion and intensification, especially in the context of cotton farming, the WAP Complex is becoming increasingly isolated (Clerici et al. 2007, Konrad 2015), leaving little space for near-natural habitats in the surrounding communal area, being composed of croplands, fallows of different ages and only few savannas in sites unsuitable for agriculture.

The complex is well-known for its role in the conservation of large mammals and typical Sudanian savanna ecosystems (e.g. Thiombiano et al. 2010, Thiombiano and Kampmann 2010, see next paragraph for more details). It is a continuous area of nine protected areas which include the trinational complex of W Regional Park (named after the course of the river Niger forming a “W” in this area), Arly National Park in Burkina Faso and Pendjari National Park in Benin connected and enlarged by protected areas of other categories and hunting reserves (Konrad 2015).

The wildlife, especially the large mammals (Lamarque 2004) and birds (Balança et al. 2007) of the WAP Complex, is well documented. Likewise, the flora of W National Park of Niger (Mahamane 2005), Arly (Ouédraogo et al. 2011), Pama (Mbayngone et al. 2008) and Pendjari (Assédé et al. 2012) have recently been documented by published checklists. The flora of the W National Park in Burkina Faso, however, remained scarcely and poorly documented.

The present checklist has been produced to fill this gap by providing a comprehensive and detailed view of the floristic diversity of the W National Park including the surrounding hunting reserves in Burkina Faso as a baseline for future projects aiming at protecting and managing the resources of the WAP Complex. We assume that the core area has a high plant diversity due to the geographical and climatic setting, as well as the diversity of its habitats.

## Materials and methods

### Study area

**Figure 1.**
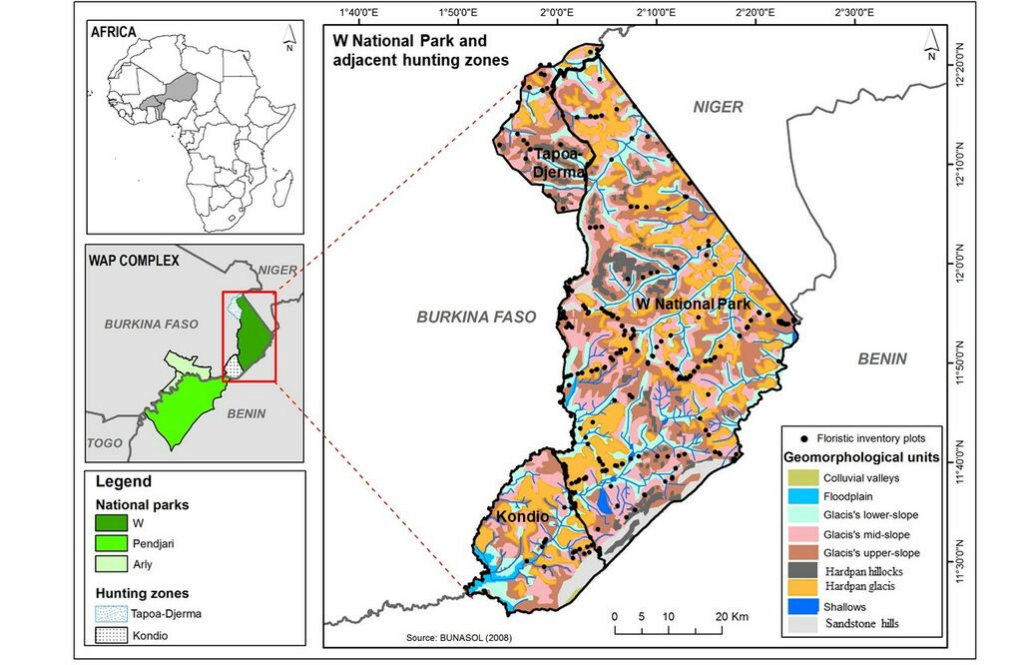
Map of W National Park in Burkina Faso and adjacent hunting zones with georeferenced occurrence records, considering different geomorphological units.

The W National Park (235,000 ha) and its two hunting reserves, Kondio (also called Kourtiagou 51,000 ha) and Tapoa Djerma (30,000 ha) are located in eastern Burkina Faso, between 12.4° and 11.4° N and 1.8° and 2.4° E (Fig. 1). The W National Park in Burkina Faso, together with its equivalents in Benin and Niger, constitute a transfrontier park that is designated “wetland of international importance” under the Ramsar Convention in 2007. Altogether, they are part of the larger W-Arly-Pendjari transfrontier complex shared amongst the three countries Benin, Burkina Faso and Niger and one of the most important West African transboundary Biosphere Reserves of UNESCO-MAB.

The national park belongs to IUCN Category II, whereas the hunting reserves belong to Category IV. Both categories act to protect large terrestrial and aquatic ecosystems, as well as threatened and rare species (both fauna and flora), serving as refuges and enhancing resilience. The National Park and the hunting zones are managed by prescribed fires ignited in October or November every year to mitigate the effect of accidental late fire and also to stimulate an off-season re-growth of perennial herbs for wildlife and to open the vegetation and increase the visibility of animals for tourists.

The W National Park and the two adjacent hunting zones of Burkina Faso are located in the Sahelo-Sudanian climate zone with an average rainfall of 750 to 950 mm, annual mean temperatures between 26 and 29°C and a dry season of 6-7 months. It belongs to the Pendjari-Mekrou biogeographical sector, shared between the Volta basin in its southern part and the Niger basin in the north. In the central western part of W National Park, eastern outliers of the Gobnangou range reach into the park and eastern outliers of the Atakora mountains are located on the southern edge of Kondio reserve and W National Park. The largest rivers in the reserves are the Mekrou on the eastern border and the Tapoa in the north, both tributaries of the Niger. In addition to the artificial water points, there are also numerous natural pools, with permanent and/or temporary water regimes (PNUD 2004). The vegetation is composed of a mosaic of various types of savannas i.e. woodland, grass, shrub and tree savanna, dry and gallery forests (Fontès and Guinko 1995), mostly distributed along elevation and soil gradients (Nacoulma et al. 2011) .

### Data collection and management

The checklist is a result of extensive field surveys by the first author during the years 2007-2010, (622 records from a species list based on 369 vegetation plots; Nacoulma 2012), as well as photo records (545 records; Dressler et al. 2014, Schmidt et al. 2016) and herbarium collections from the W National Park, including the herbaria of OUA, FR (733 and 583 records via their respective collection databases) and WAG (five records via GBIF). The vegetation plots followed standard sizes of 10 m x 10 m for the herb layer and 10 m x 50 m or 30 m x 30 m for the woody layer shown to be suitable for the characterisation of Sudanian savanna vegetation by Hahn-Hadjali (1998) and Ouédraogo (2006). They were taken mostly during the end of the rainy season (September-October) in order to maximise the number of flowering individuals for identification. The smaller plots for herbs were randomly located inside the corresponding plots for woody plants. The locations were chosen by a stratified random design using satellite images and soil maps to best represent the geomorphological units (colluvial plains: 5, floodplain: 17, lower slope glacis: 45, mid-slope glacis: 101, upper slope glacis: 54, hardpan hillocks: 18, hardpan glacis: 73, shallows: 31, sandstone hills: 25) and vegetation types (woodland: 39, gallery forest: 17, savanna woodland: 58, tree savanna: 92, shrub savanna: 134, grass savanna: 29) following the definitions of the Yangambi conference (Conseil Scientifique pour l'Afrique au Sud du Sahara 1956), while taking accessibility into account (see Fig. 1 for the plot locations and geomorphological units). Herbarium and photo records were partly taken during the afore-mentioned work on vegetation plots, partly during collecting expeditions for the assessment of Burkina Faso's flora, focussing on previously under-sampled habitats (van der Burgt et al. 2010) and in the course of other research in the area with different focus (Erpenbach et al. 2012, Schumann et al. 2016, Zwarg et al. 2012). For data analysis, all these records have been combined into an MS Access database. Species concepts and synonymy, as well as families, follow Thiombiano et al. (2012) and, for orthographic corrections of primary data, we used the Taxonomic Name Resolution Service (TNRS; http://tnrs.iplantcollaborative.org). In order to identify species new to the flora of Burkina Faso, we compared the total floristic list of the study area to the catalogue of Burkina Faso‘s vascular plants (Thiombiano et al. 2012), supplemented by new species records from Schmidt (2018). Introduced species have been identified using a list extracted from Thiombiano et al. (2012), based upon distribution notes and contributed to the Global Register of Introduced and Invasive Species (Schmidt et al. 2020).

### Species identification

Species have been identified using the Flora of West Tropical Africa (Hutchinson and Dalziel 1972), Flore Analytique du Bénin (Akoegninou et al. 2006), as well as group-specific literature for grasses (Poilecot 1995, Poilecot 1999), orchids (Perez-Vera 2003) and woody plants (Arbonnier 2000) in combination with online guides such as African Plants - a photo guide (Dressler et al. 2014) and the herbarium collections of Ouagadougou University (OUA) and Herbarium Senckenbergianum (FR).

### Notes on the checklist

The list is arranged alphabetically by family and species. Synonyms are only given if these have been used in our primary data in herbarium, vegetation plot or photo records. Herbarium records are given with collector and collector’s number, followed in parentheses by herbarium code and herbarium number. Photo records are marked with APPG (African Plants - a photo guide: Dressler et al. 2014) and the photo ID within the guide’s website and database. New country records are specifically mentioned as "New species record for Burkina Faso". Introduced species are marked by "(introduced)" following the distribution type.

## Checklists

### Vascular plants of W National Park, Burkina Faso and adjacent hunting zones

#### Acanthaceae

204FA951-7615-5CD3-83E0-EBCF61B4A24C

#### Blepharis linariifolia

Pers.

034D5643-5B59-50BC-BD73-65BCA4D424A9

##### Distribution

Sudano-Zambesian

##### Notes

Life Form: therophyte; Voucher: Schumann (APPG-3817)

#### Blepharis maderaspatensis

(L.) B.Heyne ex Roth

7FCE5CBC-6114-503C-8DFC-3156DF4AF04E

##### Distribution

Pantropical

##### Notes

Life Form: therophyte; Voucher: Zwarg 118 (FR)

#### Dicliptera paniculata

(Forssk.) I.Darbysh.

2EC12C44-90D4-5CFF-9D1E-EEBE08BEA8F9

Peristrophe paniculata (Forssk.) Brummitt|Peristrophe bicalyculata (Retz.) Nees

##### Distribution

Pluriregional African

##### Notes

Life Form: therophyte; Voucher: Nacoulma 207 (OUA-13570)

#### Dyschoriste nagchana

(Nees) Bennet

3BC45385-519B-5012-B28D-4BBAFDF1CF65

Dyschoriste perrottetii (Nees) Kuntze

##### Distribution

Pantropical

##### Notes

Life Form: therophyte; Voucher: Schmidt et al. (FR-0007461)

#### Hygrophila auriculata

(Schumach.) Heine

946633C1-A756-51B0-AA69-D5E184BF41A9

##### Distribution

Pantropical

##### Notes

Life Form: therophyte; Voucher: Schmidt et al. (FR-0007402)

#### Hygrophila senegalensis

(Nees) T.Anderson

F30824B0-6703-57F5-BE78-D97C12417CD5

##### Distribution

Sudanian

##### Notes

Life Form: geophyte; Voucher: Nacoulma 4509 (OUA-17092)

#### Hypoestes aristata

(Vahl) Roem. & Schult.

7C2E35DC-A044-5566-8A96-0F4DCA33DB33

Hypoestes verticillaris (L.f.) Sol. ex Roem. & Schult.

##### Distribution

Pantropical

##### Notes

Life Form: phanerophyte; Voucher: Schmidt et al. (FR-0007656)

#### Justicia flava

(Forssk.) Vahl

1E774733-F810-5F85-AD9C-4FBD59533DC3

##### Distribution

Afrotropical

##### Notes

Life Form: chamaephyte; Voucher: Schmidt et al. (FR-0007646)

#### Justicia insularis

T.Anderson

F8908FBA-49E9-5EB2-AB2D-5E302DEF16F5

##### Distribution

Sudano-Zambesian

##### Notes

Life Form: therophyte; Voucher: Zwarg 102 (FR)

#### Lepidagathis anobrya

Nees

804AB65E-1770-5948-A1D3-9B69AEC94E9A

##### Distribution

Sudanian

##### Notes

Life Form: chamaephyte; Voucher: Nacoulma (APPG-70171)

#### Lepidagathis collina

(Endl.) Milne-Redh.

69684AF4-D1EF-5C9E-A0B5-053C6143AF75

##### Distribution

Sudanian

##### Notes

Life Form: phanerophyte; Voucher: Nacoulma 71 (OUA-13435)

#### Monechma ciliatum

(Jacq.) Milne-Redh.

87353226-B5CE-55B1-A156-437085366DD3

##### Distribution

Afrotropical

##### Notes

Life Form: therophyte; Voucher: Zwarg 47 (FR)

#### Monechma depauperatum

(T.Anderson) C.B.Clarke

264AF0A7-5C12-5F54-95C1-1A58475CEB44

##### Distribution

Afrotropical

##### Notes

Life Form: chamaephyte; Voucher: Nacoulma (OUA-17240)

#### Nelsonia canescens

(Lam.) Spreng.

35326693-C023-5469-94A8-1761374298E2

##### Distribution

Pantropical

##### Notes

Life Form: therophyte; Voucher: Nacoulma 204 (OUA-13567)

#### Phaulopsis barteri

T.Anderson

DCD5DEC2-4B2F-503D-8DD7-2385887FB1B3

##### Distribution

Afrotropical

##### Notes

Life Form: therophyte; Voucher: Schmidt et al. (FR-0007431)

#### Alismataceae

07157D27-A444-556C-9664-6BEACCC0F0A4

#### Burnatia enneandra

Micheli

07D9D365-B012-591F-82AD-11668205A196

##### Distribution

Afrotropical

##### Notes

Life Form: hemicryptophyte; Voucher: Schmidt et al. (FR-0007398)

#### Butomopsis latifolia

(D.Don) Kunth

2392E836-B694-5888-AD04-ED5D1FA8157E

##### Distribution

Afrotropical

##### Notes

Life Form: hemicryptophyte; Voucher: Schmidt (APPG-4847)

#### Limnophyton obtusifolium

(L.) Miq.

70BC4935-3822-5F67-AD37-E2437A4EE4EE

##### Distribution

Paleotropical

##### Notes

Life Form: therophyte; Voucher: Nacoulma 4943 (OUA-17285)

#### Sagittaria guayanensis

Kunth

06C7D1C2-8EA8-5E4F-B41E-2B2CAD426915

##### Distribution

Pantropical

##### Notes

Life Form: therophyte; Voucher: Nacoulma 73 (OUA-13437)

#### Amaranthaceae

15B93C9C-E8E8-5556-9F4F-E93276DC2934

#### Achyranthes aspera

L.

669DB67E-7AD6-53EB-8F91-B8FBB8549D2A

Achyranthes argentea Lam.

##### Distribution

Pantropical

##### Notes

Life Form: chamaephyte; Voucher: Zwarg 45 (FR)

#### Alternanthera sessilis

(L.) R.Br. ex DC.

635A5BC9-1DE9-55F7-B934-7442A95C8744

##### Distribution

Pluriregional African

##### Notes

Life Form: chamaephyte; Voucher: Nacoulma 194 (OUA-13557)

#### Celosia trigyna

L.

276284FF-C7F2-589B-A1FE-4C6B19F71B52

##### Distribution

Afro-Malagasy

##### Notes

Life Form: therophyte; Voucher: Schumann 101 (FR-0083347)

#### Pandiaka angustifolia

(Vahl) Hepper

F1857B15-6074-52F1-BA65-B1E056DAB5F6

##### Distribution

Sudano-Zambesian

##### Notes

Life Form: therophyte; Voucher: Zwarg 12 (FR)

#### Pandiaka involucrata

(Moq.) B.D.Jacks.

F79C8CE4-CC58-5D3E-990A-20D4BBAA2E40

##### Distribution

Sudanian

##### Notes

Life Form: therophyte

#### Pupalia lappacea

(L.) Juss.

C2085FB7-BCE2-591A-9A0E-B1691E56A5D7

##### Distribution

Paleotropical

##### Notes

Life Form: therophyte; Voucher: Schumann (FR-0083346)

#### Amaryllidaceae

B52EA06B-6351-5967-A70E-6207EA372BF1

#### Crinum distichum

Herb.

51931D13-47C4-50FD-A73B-3CB268A85165

##### Distribution

Sudanian

##### Notes

Life Form: geophyte

#### Crinum ornatum

(Aiton) Herb.

38D13A40-CE15-55FE-BC03-6676554C5B7C

##### Distribution

Pluriregional African

##### Notes

Life Form: geophyte; Voucher: Nacoulma (APPG-69869)

#### Pancratium tenuifolium

Hochst. ex A.Rich.

2A48A4CF-0DD5-503F-A647-68AA9C477784

##### Distribution

Guineo-Congolian-Sudano-Zambesian

##### Notes

Life Form: geophyte; Voucher: Katharina Schumann (APPG-3417)

#### Pancratium trianthum

Herb.

1094A6CF-4BE6-5435-B195-D6F3C7462BCB

##### Distribution

Guineo-Congolian-Sudano-Zambesian

##### Notes

Life Form: geophyte; Voucher: Nacoulma 4586 (OUA-17164)

#### Scadoxus multiflorus

(Martyn) Raf.

D714FD6D-185F-5AF1-95AF-920572F2E701

##### Distribution

Sudano-Zambesian

##### Notes

Life Form: geophyte; Voucher: Katharina Schumann (APPG-2330)

#### Anacardiaceae

5FBF9933-2FEC-5120-A3A6-FB0B677713F6

#### Haematostaphis barteri

Hook.f.

19629F03-87A6-548C-81EF-6177E6BB6B5F

##### Distribution

Sudanian

##### Notes

Life Form: phanerophyte; Voucher: Schmidt et al. (FR-0007486)

#### Lannea acida

A.Rich.

B776A4B0-BA49-50D4-812A-853489B0EEE8

##### Distribution

Sudanian

##### Notes

Life Form: phanerophyte; Voucher: Zwarg 107 (FR)

#### Lannea barteri

(Oliv.) Engl.

D75207D3-B7E7-55ED-A960-BA8556BC87C4

##### Distribution

Sudanian

##### Notes

Life Form: phanerophyte

#### Lannea microcarpa

Engl. & K.Krause

065874F8-5858-5963-81A3-FB28520382AF

##### Distribution

Sudano-Zambesian

##### Notes

Life Form: phanerophyte; Voucher: Zwarg 60 (FR)

#### Lannea velutina

A.Rich.

32F15AA2-ECD3-5816-828A-8ECCF75EAD58

##### Distribution

Sudanian

##### Notes

Life Form: phanerophyte; Voucher: Nacoulma (APPG-70154)

#### Ozoroa obovata

(Oliv.) R. Fern. & A. Fern.

71051F27-A73C-5ADE-ACF2-97DE9A4705D9

Ozoroa insignis Delile

##### Distribution

Sudano-Zambesian

##### Notes

Life Form: phanerophyte; Voucher: Schmidt et al. (FR-0007479)

#### Ozoroa pulcherrima

(Schweinf.) R.Fern. & A.Fern.

BABBE2EC-8563-599A-8EDE-ECE74AC3AE50

##### Distribution

Sudanian

##### Notes

Life Form: phanerophyte

#### Rhus crenulata

A.Rich.

470A4DA3-6528-53D7-A737-3F6E1C2C39BE

Rhus natalensis Bernh. ex C.Krauss

##### Distribution

Afrotropical

##### Notes

Life Form: phanerophyte; Voucher: Thiombiano et al. 3308 (OUA)

#### Sclerocarya birrea

(A.Rich.) Hochst.

DAB53860-8648-516B-8E2E-30320FD7BE47

##### Distribution

Sudanian

##### Notes

Life Form: phanerophyte; Voucher: Katharina Schumann (APPG-2342)

#### Spondias mombin

L.

34648D34-1FEB-57F7-85A6-0C3CD4FBD5C2

##### Distribution

Pantropical

##### Notes

Life Form: phanerophyte; Voucher: Schmidt et al. (FR-0007644)

#### Annonaceae

96C1D496-A0E0-5D5B-A6D1-BA48E41EE75D

#### Annona senegalensis

Pers.

D126C8F0-C96A-5C1B-9D0C-511295C828CE

##### Distribution

Sudanian

##### Notes

Life Form: phanerophyte; Voucher: Zwarg 73 (FR)

#### Hexalobus monopetalus

(A.Rich.) Engl. & Diels

4319AB17-0F29-509F-9980-3B24BA675606

##### Distribution

Sudanian

##### Notes

Life Form: phanerophyte; Voucher: Nacoulma 147 (OUA-13511)

#### Uvaria chamae

P.Beauv.

DFD1DE13-5817-512A-B950-E1607857791F

##### Distribution

Pluriregional African

##### Notes

Life Form: phanerophyte; Voucher: Nacoulma 4949 (OUA-17281)

#### Xylopia aethiopica

(Dunal) A.Rich.

09D16DCF-3E1D-5419-8AEF-0E230F0D3D5D

##### Distribution

Afrotropical

##### Notes

Life Form: phanerophyte

#### Apocynaceae

29061D4E-F476-556B-9FA6-E9DD6C1E4A75

#### Baissea multiflora

A.DC.

731EB664-4336-54A7-A8A3-5B8EA7F2604C

##### Distribution

Sudanian

##### Notes

Life Form: phanerophyte; Voucher: Nacoulma 168 (OUA-13532)

#### Calotropis procera

(Aiton) Dryand.

4BB4C6C0-3322-5F57-8302-08463FDF7A83

##### Distribution

Paleotropical

##### Notes

Life Form: phanerophyte; Voucher: Schumann (APPG-471)

#### Caralluma adscendens

(Roxb.) R.Br.

EB3E3E4C-DBC4-585F-9E7B-788631A4F311

##### Distribution

Sudano-Zambesian

##### Notes

Life Form: chamaephyte; Voucher: Schumann (APPG-493)

#### Ceropegia fusiformis

N.E.Br.

4C6C993F-8434-59DC-82D7-6A9C2A46604C

##### Distribution

Paleotropical

##### Notes

Life Form: phanerophyte

#### Ceropegia racemosa

N.E.Br.

24B11867-AA14-578A-AE18-557540199878

##### Distribution

Afrotropical

##### Notes

Life Form: phanerophyte; Voucher: Nacoulma 57 (OUA-13422)

#### Ceropegia rhynchantha

Schltr.

1BDB9516-0F6C-533C-91D9-6EA54DAA704F

##### Distribution

Sudanian

##### Notes

Life Form: geophyte; Voucher: Schumann (FR-0083341)

#### Cryptolepis oblongifolia

(Meisn.) Schltr.

B33083A9-099A-5D3D-A307-66380A831044

Ectadiopsis oblongifolia (Meisn.) B.D.Jacks.

##### Distribution

Pluriregional African

##### Notes

Life Form: phanerophyte; Voucher: Nacoulma 90 (OUA-13454)

#### Gymnema sylvestre

(Retz.) R.Br. ex Sm.

2C4C174E-C249-5B77-A9DE-5940B0DA58EF

##### Distribution

Pantropical

##### Notes

Life Form: therophyte; Voucher: Nacoulma 11 (OUA-13381)

#### Leptadenia hastata

Vatke

D0FBDB87-C6D4-5F56-9B17-9BCD610523AD

##### Distribution

Sudanian

##### Notes

Life Form: phanerophyte; Voucher: Nacoulma (APPG-70173)

#### Oxystelma bornouense

R.Br.

7BA8F3AF-B7BB-532A-B275-E6CB1572FD6F

##### Distribution

Guineo-Congolian-Sudano-Zambesian

##### Notes

Life Form: phanerophyte; Voucher: Schumann (APPG-3866)

#### Pergularia daemia

(Forssk.) Chiov.

24713C4F-4625-59F7-940E-19B110B67C21

##### Distribution

Pluriregional African

##### Notes

Life Form: phanerophyte; Voucher: Schumann (FR-0083339)

#### Raphionacme brownii

Scott-Elliot

6EC55AAE-A33F-5C20-A1F9-D66F263FD05F

##### Distribution

Sudanian

##### Notes

Life Form: geophyte; Voucher: Nacoulma 4542 (OUA-17120)

#### Saba comorensis

(Bojer ex A.DC.) Pichon

F25F98E3-68D1-5E43-BEC7-0CA559C655C0

##### Distribution

Pluriregional African

##### Notes

Life Form: phanerophyte; Voucher: Nacoulma 150 (OUA-13514)

#### Saba senegalensis

(A.DC.) Pichon

37ED67BF-E2E1-5FCA-9651-BA45773777ED

##### Distribution

Sudanian

##### Notes

Life Form: phanerophyte; Voucher: Schmidt et al. (FR-0007685)

#### Sarcostemma viminale

(L.) R.Br.

E25D9C60-04D2-5F30-BFD6-0EBEBFA43849

##### Distribution

Pantropical

##### Notes

Life Form: phanerophyte

#### Secamone afzelii

(Roem. & Schult.) K.Schum.

D9672F7A-4EED-5626-841D-EFA0D4C3FAD4

##### Distribution

Guineo-Congolian

##### Notes

Life Form: phanerophyte; Voucher: Schumann (FR-0083338)

#### Sphaerocodon caffrum

(Meisn.) Schltr.

192D8C66-B5AE-568B-899D-8CF8C684B49C

##### Distribution

Afrotropical

##### Notes

Life Form: phanerophyte; Voucher: Nacoulma 64 (OUA-13429)

#### Strophanthus sarmentosus

DC.

28696C1E-AC14-52EC-8C54-0DC4075CA6B6

##### Distribution

Guineo-Congolian

##### Notes

Life Form: phanerophyte; Voucher: Nacoulma 4488 (OUA-17078)

#### Tacazzea apiculata

Oliv.

2A1A0D33-4DA3-58A6-9598-7178E8CEBD74

##### Distribution

Afrotropical

##### Notes

Life Form: phanerophyte; Voucher: Nacoulma 211 (OUA-13574)

#### Aponogetonaceae

6B458D91-14F8-579A-A150-9244F2E537AB

#### Aponogeton vallisnerioides

Baker

19376500-9B0E-551B-B09D-50E51DC8D67F

##### Distribution

Guineo-Congolian

##### Notes

Life Form: hydrophyte; Voucher: Nacoulma (APPG-70417); New species record for Burkina Faso.

#### Araceae

2B7619C4-F9C9-5577-993F-E4BDCAACCD58

#### Amorphophallus abyssinicus

(A.Rich.) N.E.Br.

BF9E2BF0-8CA1-5E89-B2C4-4A1E1BA8343F

##### Distribution

Sudanian

##### Notes

Life Form: geophyte; Voucher: Nacoulma (APPG-69725)

#### Amorphophallus baumannii

(Engl.) N.E.Br.

8A91AA9C-4162-5F4F-BEE7-5ADC7254616F

Amorphophallus flavovirens N.E.Br.

##### Distribution

Sudanian

##### Notes

Life Form: geophyte; Voucher: Nacoulma (APPG-69730)

#### Amorphophallus dracontioides

(Engl.) N.E.Br.

37E2A001-1B8E-56C1-A0A6-E569C2C675B9

##### Distribution

Sudanian

##### Notes

Life Form: geophyte; Voucher: Nacoulma (APPG-69734)

#### Anchomanes difformis

(Blume) Engl.

EF00EB05-C990-5CEA-84AA-B82B4A881A13

##### Distribution

Guineo-Congolian-Sudano-Zambesian

##### Notes

Life Form: geophyte

#### Stylochaeton hypogaeus

Lepr.

339AB582-C75F-5A18-88E8-256AE6B5EE74

##### Distribution

Sudanian

##### Notes

Life Form: geophyte; Voucher: Nacoulma (APPG-70382)

#### Stylochaeton lancifolius

Kotschy & Peyr.

564D9E82-28C9-5932-BAC1-3B7B3EC6A9A9

##### Distribution

Afrotropical

##### Notes

Life Form: geophyte; Voucher: Erpenbach (APPG-30290)

#### Arecaceae

A12C0D7B-A91A-54CE-A1A2-A6CB2A887869

#### Borassus aethiopum

Mart.

2F021758-055E-5CDB-9E7E-600866796131

##### Distribution

Sudano-Zambesian

##### Notes

Life Form: phanerophyte; Voucher: Nacoulma (APPG-69777)

#### Hyphaene thebaica

(L.) Mart.

60CDB2E4-4CD5-5C64-BD45-F3BEBFE5EB05

##### Distribution

Pluriregional African

##### Notes

Life Form: phanerophyte; Voucher: Zizka (APPG-3842)

#### Aristolochiaceae

AA2A10AA-C590-5178-8B72-A36757845F9C

#### Aristolochia albida

Duch.

8CF9D169-110F-536A-8930-BA1C2BB11DDD

##### Distribution

Sudanian

##### Notes

Life Form: phanerophyte; Voucher: Nacoulma (APPG-69746)

#### Asparagaceae

E9ED2DDF-FBD8-5278-8AF4-0D0713F5821A

#### Albuca nigritana

(Baker) Troupin

D2F39183-80A6-52A0-AC84-78077F93B256

Urginea nigritana Baker

##### Distribution

Sudano-Zambesian

##### Notes

Life Form: geophyte; Voucher: Schumann (FR-0083223)

#### Asparagus africanus

Lam.

7CA808EF-7F8D-50B3-A1CA-1EAF2B1CB368

##### Distribution

Paleotropical

##### Notes

Life Form: geophyte

#### Asparagus flagellaris

(Kunth) Baker

C39E04AD-A4FE-51AC-AC7E-5A34DB52E979

##### Distribution

Sudano-Zambesian

##### Notes

Life Form: phanerophyte; Voucher: Nacoulma (APPG-69752)

#### Chlorophytum blepharophyllum

Schweinf. ex Baker

A1355237-698B-5524-B3E8-0998F3D2FA3F

##### Distribution

Sudano-Zambesian

##### Notes

Life Form: geophyte; Voucher: Nacoulma 4588 (OUA-17187)

#### Chlorophytum gallabatense

Schweinf. ex Baker

D2985372-3BA8-5CC2-9916-231CA60764DE

##### Distribution

Sudanian

##### Notes

Life Form: geophyte

#### Chlorophytum geophilum

Peter ex Poelln.

BE5B54FF-B66A-5161-BDE5-61D080C522FA

##### Distribution

Sudanian

##### Notes

Life Form: geophyte

#### Chlorophytum laxum

R.Br.

88EA3A72-CEBC-545C-81F4-03A01090B0C8

##### Distribution

Sudanian

##### Notes

Life Form: geophyte; Voucher: Zwarg 108 (FR)

#### Chlorophytum macrophyllum

(A.Rich.) Asch.

7A42E59C-8FC9-527C-A4AC-88AE4328D258

##### Distribution

Sudano-Zambesian

##### Notes

Life Form: hemicryptophyte; Voucher: Georg Zizka (APPG-3436)

#### Chlorophytum orchidastrum

Lindl.

369CED60-EE7D-5A97-8B57-1D4D0341B829

##### Distribution

Guineo-Congolian-Sudano-Zambesian

##### Notes

Life Form: hemicryptophyte; Voucher: Schmidt et al. (FR-0007465)

#### Chlorophytum stenopetalum

Baker

46E93D72-46D4-57EA-BAB3-6A857F08B899

##### Distribution

Sudanian

##### Notes

Life Form: geophyte; Voucher: Nacoulma 4558 (OUA-17135)

#### Drimia altissima

(L.f.) Ker Gawl.

B6A42A8A-9499-5577-AFBD-1D3AA7FDD0C7

Urginea altissima (L.f.) Baker

##### Distribution

Afrotropical

##### Notes

Life Form: hemicryptophyte

#### Drimiopsis barteri

Baker

6C32A654-2589-58AB-B992-97D90BFE6396

##### Distribution

Sudanian

##### Notes

Life Form: geophyte; Voucher: Nacoulma (APPG-70165); New species record for Burkina Faso.

#### Eriospermum flagelliforme

(Baker) J.C.Manning

BB2A28C4-0F6E-5D31-8CF8-555038996E90

Eriospermum abyssinicum Baker

##### Distribution

Afrotropical

##### Notes

Life Form: geophyte; Voucher: Zwarg 52 (FR-25053)

#### Ledebouria sudanica

(A.Chev.) Burg

0D1E3A3D-4619-5E57-A3BE-6273944DD229

##### Distribution

Sudanian

##### Notes

Life Form: geophyte; Voucher: Nacoulma (APPG-70170)

#### Ornithogalum viride

(L.) J.C.Manning & Goldblatt

9FA203CD-CD7C-57A0-A0C3-4C872F27E3AF

##### Distribution

Sudano-Zambesian

##### Notes

Life Form: geophyte

#### Sansevieria liberica

Gérôme & Labroy

DFFB2C6B-EAFE-5EB8-8E09-9524441FA66E

##### Distribution

Guineo-Congolian-Sudano-Zambesian

##### Notes

Life Form: geophyte; Voucher: Nacoulma (APPG-70343)

#### Asteraceae

3B351FBF-FE68-5A93-9683-D0273BED1D55

#### Acanthospermum hispidum

DC.

631D228C-8ADE-5980-843C-4DD935109FB7

##### Distribution

Afro-Malagasy (introduced)

##### Notes

Life Form: therophyte; Voucher: Schmidt (APPG-4421)

#### Aedesia glabra

(Klatt) O.Hoffm.

B5D57803-4DFF-5126-89B2-DB1F88032515

Aedesia baumannii O.Hoffm.

##### Distribution

Guineo-Congolian

##### Notes

Life Form: phanerophyte

#### Ageratum conyzoides

L.

0E8F8693-DB03-545E-8450-DE0258FD3156

##### Distribution

Pantropical (introduced)

##### Notes

Life Form: therophyte

#### Aspilia africana

(Pers.) C.D.Adams

ECDBABCC-9449-588A-9AC6-79831F1B5443

##### Distribution

Guineo-Congolian

##### Notes

Life Form: therophyte; Voucher: Nacoulma 4578 (OUA-17161)

#### Aspilia angustifolia

Oliv. & Hiern

E1A51B9A-C2C9-5FDB-A8C1-A65FDD23B128

##### Distribution

Sudanian

##### Notes

Life Form: therophyte

#### Aspilia bussei

O.Hoffm. & Muschl.

B14D8821-5289-5D47-9FE2-23765092A1DE

##### Distribution

Sudanian

##### Notes

Life Form: therophyte; Voucher: Zwarg 17 (FR)

#### Aspilia helianthoides

(Schumach. & Thonn.) Oliv. & Hiern

8C238E1D-3D64-55A7-9788-55429B127D13

##### Distribution

Sudanian

##### Notes

Life Form: therophyte

#### Aspilia kotschyi

(Sch.Bip. ex Hochst.) Oliv.

E78154FB-517F-552D-803A-559EF72A1802

##### Distribution

Sudano-Zambesian

##### Notes

Life Form: therophyte

#### Aspilia paludosa

Berhaut

26433B89-44E4-5950-B851-1E614D142DA9

##### Distribution

Sudanian

##### Notes

Life Form: therophyte; Voucher: Zwarg 71 (FR)

#### Aspilia rudis

Oliv. & Hiern

CFA44BAD-790E-5B09-991D-C75B01D9AD5A

##### Distribution

Sudanian

##### Notes

Life Form: therophyte; Voucher: Schmidt et al. (FR-0007429)

#### Bidens borianiana

(Sch.Bip. ex Schweinf.) Cufod.

DD4D7FD5-EE38-5186-B7FA-15D40E2E1BFF

##### Distribution

Sudanian

##### Notes

Life Form: therophyte; Voucher: Nacoulma 97 (OUA-13461)

#### Bidens engleri

O.E.Schulz

DFC01B10-7C09-5836-9B62-5FA58DF05065

##### Distribution

Sudanian

##### Notes

Life Form: therophyte; Voucher: Nacoulma 4534 (OUA-17110)

#### Bidens pilosa

L.

30B63F12-875F-5362-B7F5-775941AFB21F

##### Distribution

Sudanian (introduced)

##### Notes

Life Form: therophyte

#### Blumea crispata

(Vahl) Merxm.

3A7123FE-C025-5226-A2C5-AB6C068D1435

##### Distribution

Pluriregional African

##### Notes

Life Form: phanerophyte; Voucher: Nacoulma 85 (OUA-13449)

#### Blumea oloptera

DC.

AC64987D-0BA0-5AE0-B086-736D46B52E70

Laggera oloptera (DC.) C.D.Adams

##### Distribution

Sudano-Zambesian

##### Notes

Life Form: phanerophyte

#### Chrysanthellum indicum

DC.

F9611A08-92D4-51A1-93FE-94182B989724

##### Distribution

Pantropical (introduced)

##### Notes

Life Form: therophyte

#### Dicoma tomentosa

Cass.

AFDCD77E-1BD6-58EB-9329-E8A1E5AD8907

##### Distribution

Sudano-Zambesian

##### Notes

Life Form: therophyte

#### Linzia purpurea

(Sch.Bip. ex Walp.) Isawumi

6EBC2080-8226-52B0-BB9E-B496676CCE0D

Vernonia purpurea Sch.Bip. ex Walp.

##### Distribution

Sudano-Zambesian

##### Notes

Life Form: therophyte; Voucher: Nacoulma 36 (OUA-13401)

#### Macledium sessiliflorum

(Harv.) S.Ortiz

D9415725-A890-554C-93A0-380848ED3D97

Dicoma sessiliflora Harv.

##### Distribution

Afrotropical

##### Notes

Life Form: hemicryptophyte; Voucher: Nacoulma (APPG-70184)

#### Melanthera elliptica

O.Hoffm.

86B585A1-3E14-5BC8-A2BC-07F52E0A3EB6

##### Distribution

Sudanian

##### Notes

Life Form: hemicryptophyte; Voucher: Nacoulma 79 (OUA-13443)

#### Melanthera scandens

(Schumach. & Thonn.) Roberty

E4CA15BC-08D3-59B0-979D-D87D8799703F

##### Distribution

Afrotropical

##### Notes

Life Form: therophyte; Voucher: Nacoulma 4627 (OUA-17218)

#### Pentanema indicum

(L.)

96E106B8-2A01-50BA-B221-33A5CADD8604

##### Distribution

Afrotropical

##### Notes

Life Form: therophyte

#### Pseudognaphalium luteoalbum

(L.) Hilliard & B.L.Burtt

3C2EA326-A70E-5325-B3E0-7E8CB83A5831

##### Distribution

Cosmopolitan

##### Notes

Life Form: therophyte; Voucher: Nacoulma (OUA-17193)

#### Synedrella nodiflora

(L.) Gaertn.

B6DF2654-D6C0-5A17-80FE-ED730E18E477

##### Distribution

Pantropical

##### Notes

Life Form: therophyte; Voucher: Schumann (FR-0083332)

#### Tridax procumbens

L.

707DFFF1-121E-5DD0-9809-168962D44688

##### Distribution

Pantropical (introduced)

##### Notes

Life Form: therophyte; Voucher: Schumann (FR-0083331)

#### Vernonia colorata

(Willd.) Drake

534B0493-89BE-5F81-BC97-5B861A53DBFC

##### Distribution

Sudano-Zambesian

##### Notes

Life Form: phanerophyte; Voucher: Schmidt et al. (FR-0007421)

#### Vernonia pumila

(Vell.) Cabrera

598BF7E9-4405-5731-9FA6-E3EFE0660B61

##### Distribution

Sudano-Zambesian

##### Notes

Life Form: therophyte

#### Bignoniaceae

7002E62E-1186-59B0-B3CA-E246723B77DA

#### Kigelia africana

(Lam.) Benth.

ED758433-9B08-5700-8B09-18173BB2B0F5

##### Distribution

Sudano-Zambesian

##### Notes

Life Form: phanerophyte; Voucher: Nacoulma 107 (OUA-13471)

#### Stereospermum kunthianum

Cham.

4B9973A1-9262-5892-85B9-C78C4ADB695F

##### Distribution

Sudanian

##### Notes

Life Form: phanerophyte; Voucher: Katharina Schumann (APPG-2499)

#### Bixaceae

40EDB8AA-9442-525C-BBA1-5EC29BB1D7AB

#### Cochlospermum planchonii

Hook.f. ex Planch.

874339AD-5B80-53FF-BA41-2578AAE0ADE9

##### Distribution

Sudanian

##### Notes

Life Form: chamaephyte; Voucher: Zwarg 83 (FR)

#### Cochlospermum tinctorium

Perrier ex A.Rich.

A66346EF-7EC2-55FE-AA1D-BFA004381568

##### Distribution

Sudanian

##### Notes

Life Form: geophyte; Voucher: Nacoulma (APPG-69817)

#### Boraginaceae

933669E2-77F9-5BA5-859A-0589E5400DFA

#### Euploca strigosa

(Willd.) Diane & Hilger

340E7017-FC3F-52E9-BD58-899FBA77EA32

Heliotropium strigosum Willd.

##### Distribution

Paleotropical

##### Notes

Life Form: chamaephyte; Voucher: Nacoulma 4476 (OUA-17054)

#### Heliotropium indicum

L.

46F49ABA-E8CF-5F59-BC86-1D526EBC18A1

##### Distribution

Pantropical

##### Notes

Life Form: therophyte; Voucher: Nacoulma (APPG-70063)

#### Rotula aquatica

Lour.

6D941A2C-497F-514D-A937-67C987880574

##### Distribution

Pantropical

##### Notes

Life Form: phanerophyte; Voucher: Nacoulma (APPG-70332)

#### Burseraceae

9689A54B-2D86-5804-85E1-A8653B77230B

#### Boswellia dalzielii

Hutch.

9FCF752E-5CD4-56DC-9A80-27D1D1221A56

##### Distribution

Sudanian

##### Notes

Life Form: phanerophyte

#### Commiphora africana

(A.Rich.) Endl.

B4A05F56-015F-5D00-8B8A-466E1E3EB6CA

##### Distribution

Paleotropical

##### Notes

Life Form: phanerophyte

#### Campanulaceae

EB98B4B0-931B-5977-888A-6A6EB22F7752

#### Wahlenbergia hirsuta

(Edgew.) Tuyn

2B31BC06-9200-5AB9-986A-7FA2651DD3C7

##### Distribution

Afro-American

##### Notes

Life Form: therophyte; Voucher: Schumann (FR-0083327)

#### Cannabaceae

781105EC-B646-5A52-9086-01CA829BE048

#### Trema orientalis

(L.) Blume

68D435EC-4185-5094-A26C-568503EE9474

##### Distribution

Afro-Malagasy

##### Notes

Life Form: phanerophyte; Voucher: Schmidt et al. (FR-0007626)

#### Capparaceae

43AD3D10-5639-5D41-9F15-98ED9904AE3E

#### Boscia angustifolia

A.Rich.

24347CAD-D3B4-5185-9CDF-323EF8EB60BC

##### Distribution

Sudanian

##### Notes

Life Form: phanerophyte; Voucher: Thiombiano et al. 3297 (OUA)

#### Boscia senegalensis

Lam.

76FEEE91-9F30-56E6-AD88-C438E663643B

##### Distribution

Sudanian

##### Notes

Life Form: phanerophyte; Voucher: Nacoulma (APPG-69783)

#### Cadaba farinosa

Forssk

9B3AD6C4-2ACC-5CA3-B107-0496CFA81C7C

##### Distribution

Paleotropical

##### Notes

Life Form: phanerophyte; Voucher: Erpenbach (APPG-29466)

#### Capparis sepiaria

L.

B296A2F2-FEF7-5E1B-A24C-351ACC268F23

##### Distribution

Sudanian

##### Notes

Life Form: phanerophyte

#### Capparis tomentosa

Lam.

24BAD4CA-8BB4-5690-9FD0-2A382D9E5986

##### Distribution

Sudanian

##### Notes

Life Form: phanerophyte; Voucher: Katharina Schumann (APPG-4121)

#### Crateva adansonii

DC.

7666F1F1-2CEA-528F-9E7A-72D9A9308173

##### Distribution

Paleotropical

##### Notes

Life Form: phanerophyte; Voucher: Nacoulma 140 (OUA-13504)

#### Maerua angolensis

DC.

9C21385A-EA82-5886-9952-07F59E289581

##### Distribution

Afrotropical

##### Notes

Life Form: phanerophyte; Voucher: Nacoulma (APPG-70194)

#### Maerua oblongifolia

(Forssk.) A.Rich.

1E8E7E7A-1A8A-5211-AE29-F4B2B7B0F17D

##### Distribution

Sudanian

##### Notes

Life Form: phanerophyte; Voucher: Nacoulma (APPG-70196)

#### Ritchiea capparoides

(Andrews) Britten

6D6B685E-5319-5811-BD86-3EE6D04BE35A

##### Distribution

Guineo-Congolian

##### Notes

Life Form: phanerophyte; Voucher: Thiombiano et al. 3311 (OUA)

#### Caryophyllaceae

0E1F012F-89C9-5066-9F0E-A22D4C03F6B4

#### Polycarpaea corymbosa

(L.) Lam.

F9BECE56-2C2C-578E-A052-B41CC6900637

##### Distribution

Pluriregional African

##### Notes

Life Form: therophyte; Voucher: Nacoulma 94 (OUA-13458)

#### Polycarpaea eriantha

Hochst. ex A.Rich.

23581C11-FA53-5BE7-9E06-983CC2014D54

##### Distribution

Sudano-Zambesian

##### Notes

Life Form: therophyte; Voucher: Schumann (FR-0083322)

#### Polycarpaea linearifolia

(DC.) DC.

CD221422-54F0-578A-AF59-E7C718E69724

##### Distribution

Afrotropical

##### Notes

Life Form: therophyte; Voucher: Schumann (FR-0083321)

#### Polycarpon prostratum

(Forssk.) Asch. & Schweinf.

99D38F74-D59E-50CD-9B69-37B9B0D342F9

##### Distribution

Paleotropical

##### Notes

Life Form: therophyte; Voucher: Nacoulma (OUA-17195)

#### Celastraceae

586360A1-78E8-5AAA-AF8D-07F9137CE2B4

#### Gymnosporia senegalensis

(Lam.) Loes.

**Figure 3.**
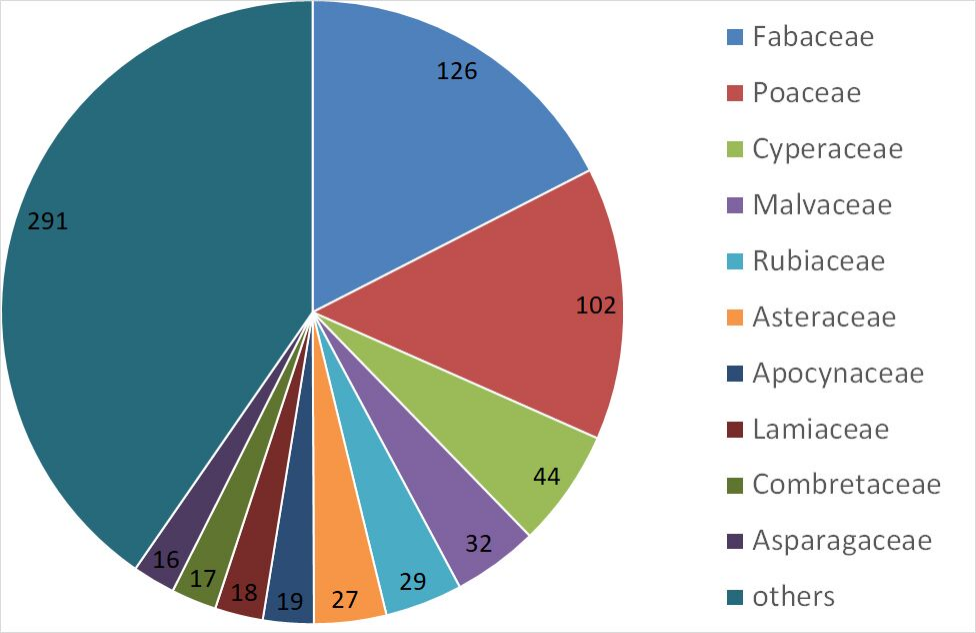
Family spectrum of the flora of the W National Park with number of species for the 10 largest plant families in the study area and all remaining families.

6F74ABB9-3ADB-576E-A3BF-05AE705CF1AC

##### Distribution

Sudano-Zambesian

##### Notes

Life Form: phanerophyte; Voucher: Zwarg 103 (FR)

#### Loeseneriella africana

(Willd.) R.Wilczek

7E9B62D3-623A-5D7B-8D43-2F340279435C

##### Distribution

Afrotropical

##### Notes

Life Form: phanerophyte; Voucher: Nacoulma 125 (OUA-13489)

#### Chrysobalanaceae

7A07CE3B-6306-510B-8682-E17C7EF4CDC9

#### Parinari congensis

Didr.

A5BBBC7E-8F74-5B5B-8C02-01B7F01878AF

##### Distribution

Guineo-Congolian

##### Notes

Life Form: phanerophyte; Voucher: Schmidt et al. (FR-0007622)

#### Parinari curatellifolia

Planch. ex Benth.

5B0BDF04-CC9E-5412-AC96-7E4C01C27FF6

##### Distribution

Sudano-Zambesian

##### Notes

Life Form: phanerophyte; Voucher: Nacoulma (APPG-70297)

#### Cleomaceae

56C21E69-1696-5755-BCB1-C9137F6D7B48

#### Cleome viscosa

L.

5E24A1C3-A8FA-5019-84B5-61D127805064

##### Distribution

Pantropical

##### Notes

Life Form: therophyte

#### Clusiaceae

6206D7E7-E6F0-55F6-A876-F114DC726FC0

#### Garcinia livingstonei

T.Anderson

7174181D-3176-5696-B956-1D139B603C1D

##### Distribution

Sudano-Zambesian

##### Notes

Life Form: phanerophyte; Voucher: Schmidt et al. (FR-0007444)

#### Colchicaceae

D5088805-F785-5864-9FE5-F4DFE72DB7C4

#### Gloriosa simplex

L.

562F82EA-D457-5423-ABB0-1AFB560D85E1

##### Distribution

Pluriregional African

##### Notes

Life Form: geophyte; Voucher: Nacoulma 214 (OUA-13577)

#### Gloriosa superba

L.

10F2B297-1A0B-5AAD-A0EB-061BC4A3612C

##### Distribution

Paleotropical

##### Notes

Life Form: geophyte; Voucher: Nacoulma 27 (OUA-13393)

#### Combretaceae

E99A0344-1E5A-570F-8C77-B1BD634E3D01

#### Anogeissus leiocarpa

(DC.) Guill. & Perr.

5C69C1FE-0210-516F-AC2D-07977BEEBA4B

Terminalia leiocarpa (DC.) Baill.

##### Distribution

Sudanian

##### Notes

Life Form: phanerophyte; Voucher: Schmidt et al. (FR-0007383)

#### Combretum aculeatum

Vent.

71E25779-BB47-5602-9881-81AB11BF3DA4

##### Distribution

Sudano-Zambesian

##### Notes

Life Form: phanerophyte; Voucher: Nacoulma (APPG-69837)

#### Combretum adenogonium

Steud. ex A.Rich.

6CD594B2-45AC-5A76-BD43-DDB2CEB58A31

##### Distribution

Sudanian

##### Notes

Life Form: phanerophyte

#### Combretum collinum

Fresen.

954148AF-90F0-529A-95A4-1391BCF1FF76

##### Distribution

Afrotropical

##### Notes

Life Form: phanerophyte; Voucher: Zwarg 16 (FR)

#### Combretum glutinosum

Perr. ex DC.

05186AC6-6B79-5709-947C-47C124B83541

##### Distribution

Sudanian

##### Notes

Life Form: phanerophyte; Voucher: Küppers 468 (FR-0011271)

#### Combretum micranthum

G.Don

4FDBBBBB-05B9-5685-A94E-63604024D843

##### Distribution

Sudanian

##### Notes

Life Form: phanerophyte; Voucher: Zwarg 120 (FR)

#### Combretum molle

R.Br. ex G.Don

4A758E05-8B95-543C-8E56-79B1EE70BA56

##### Distribution

Afrotropical

##### Notes

Life Form: phanerophyte; Voucher: Zwarg 91 (FR)

#### Combretum nigricans

Lepr. ex Guill. & Perr.

0634044F-B4A6-53A2-ABD6-74F538479751

##### Distribution

Sudanian

##### Notes

Life Form: phanerophyte; Voucher: Zwarg 15 (FR)

#### Combretum nioroense

Aubrév. ex Keay

F02915D7-9938-5AE1-8343-A731AC025D2F

##### Distribution

Sudanian

##### Notes

Life Form: phanerophyte; Voucher: Nacoulma (APPG-69849)

#### Combretum paniculatum

Vent.

243A2F76-915A-53E5-B508-558CB0D4147E

##### Distribution

Pluriregional African

##### Notes

Life Form: phanerophyte; Voucher: Nacoulma (APPG-69851)

#### Guiera senegalensis

J.F.Gmel.

59B37EB0-C478-5536-83E2-E235C64D6CB7

##### Distribution

Sudanian

##### Notes

Life Form: phanerophyte; Voucher: Zwarg 79 (FR)

#### Pteleopsis suberosa

Engl. & Diels

84FD16D6-ECF4-5A60-821E-55C30DEF4398

Terminalia engleri Gere & Boatwr.

##### Distribution

Pluriregional African

##### Notes

Life Form: phanerophyte; Voucher: Zwarg 95 (FR)

#### Terminalia avicennioides

Guill. & Perr.

91E49641-7AE1-580F-A4FB-5ECF6D4FD615

##### Distribution

Sudanian

##### Notes

Life Form: phanerophyte; Voucher: Katharina Schumann (APPG-2595)

#### Terminalia laxiflora

Engl.

A2F7DEB5-3672-5E54-8F83-33DE7967825E

##### Distribution

Sudanian

##### Notes

Life Form: phanerophyte; Voucher: Nacoulma 4539 (OUA-17106)

#### Terminalia macroptera

Guill. & Perr.

75AF9351-B38B-5306-8297-93A4635A76FE

##### Distribution

Sudanian

##### Notes

Life Form: phanerophyte; Voucher: Zwarg 132 (FR)

#### Terminalia mollis

M.A.Lawson

54E56E73-3A77-5E2A-BAC3-0C008939CEEC

##### Distribution

Pluriregional African

##### Notes

Life Form: phanerophyte

#### Terminalia schimperiana

Hochst. ex Delile

E8A13432-9AC7-50E4-823D-1FC4308A5A88

##### Distribution

Pantropical

##### Notes

Life Form: phanerophyte

#### Commelinaceae

0BA4A5EE-FDFB-5B37-8522-9EA5AEFB9EAB

#### Aneilema lanceolatum

Benth.

86F0CA4C-616D-5E81-836A-AED4EA419F88

##### Distribution

Sudano-Zambesian

##### Notes

Life Form: chamaephyte; Voucher: Nacoulma 4586 (OUA-17188)

#### Aneilema paludosum

A.Chev.

3C6754B2-2A87-59CC-B0A5-8544E436F2F6

##### Distribution

Sudano-Zambesian

##### Notes

Life Form: chamaephyte; Voucher: Nacoulma 4552 (OUA-17141)

#### Aneilema setiferum

A.Chev.

5937D608-E50D-5B89-BDF7-02E007419EBD

##### Distribution

Sudanian

##### Notes

Life Form: chamaephyte; Voucher: Nacoulma 4527 (OUA-17117)

#### Commelina benghalensis

L.

D081128D-8CE7-5510-A116-AFFFB8416721

##### Distribution

Paleotropical

##### Notes

Life Form: therophyte

#### Commelina erecta

L.

A0CC6998-1C61-5D57-B6DF-68C88B1354F1

##### Distribution

Pantropical

##### Notes

Life Form: therophyte; Voucher: Schmidt et al. (FR-0007618)

#### Commelina forskaolii

Vahl

6CF346EA-04FE-5CA8-97DE-4065943472BC

##### Distribution

Sudano-Zambesian

##### Notes

Life Form: therophyte; Voucher: Nacoulma 195 (OUA-13558)

#### Commelina nigritana

Benth.

D61A4BE0-F310-5BAF-9567-D8DB676A1C91

##### Distribution

Afrotropical

##### Notes

Life Form: therophyte; Voucher: Zwarg 31 (FR)

#### Commelina subulata

Roth

866A11A4-6490-57C6-BF23-5BD146BE8664

##### Distribution

Sudano-Zambesian

##### Notes

Life Form: therophyte; Voucher: Nacoulma 4495 (OUA-17071)

#### Cyanotis caespitosa

Kotschy & Peyr.

BFC4301A-6602-53CD-85F4-2B5490052A4F

##### Distribution

Afrotropical

##### Notes

Life Form: geophyte; Voucher: Nacoulma (APPG-69902)

#### Cyanotis lanata

Benth.

A767D6E6-E308-5F4D-886F-720D2CA392D7

##### Distribution

Pluriregional African

##### Notes

Life Form: therophyte

#### Cyanotis longifolia

Benth.

368D38EC-055C-553E-9961-EAAB2D9AC211

##### Distribution

Sudanian

##### Notes

Life Form: therophyte; Voucher: Nacoulma 232 (OUA-17225)

#### Floscopa africana

(P.Beauv.) C.B.Clarke

82AF7E18-26DE-5CBE-8993-495EE9C40393

##### Distribution

Pantropical

##### Notes

Life Form: geophyte

#### Floscopa axillaris

(Poir.) C.B.Clarke

C7D79FF8-74D2-510F-B90E-9ACF520D796A

##### Distribution

Pantropical

##### Notes

Life Form: geophyte

#### Murdannia simplex

(Vahl) Brenan

854DE27F-28D4-51B4-A072-7C7F3D34DA2C

##### Distribution

Paleotropical

##### Notes

Life Form: therophyte; Voucher: Schmidt et al. (FR-0007660)

#### Convolvulaceae

D8EB86F2-65DC-5ACC-B4D2-E13F93C15D54

#### Evolvulus alsinoides

(L.) L.

0D78E47A-051C-5FA8-AA26-818C15950AA6

##### Distribution

Pantropical

##### Notes

Life Form: therophyte; Voucher: Zwarg 101 (FR)

#### Ipomoea aquatica

Forssk.

75C8DE12-3BCC-5B57-B00C-E2A8C4927ABD

##### Distribution

Pantropical

##### Notes

Life Form: hemicryptophyte; Voucher: Nacoulma (APPG-70073)

#### Ipomoea argentaurata

Hallier f.

73CD41F4-9628-5CA4-BB35-504A214A9DE0

##### Distribution

Sudanian

##### Notes

Life Form: therophyte; Voucher: Nacoulma 106 (OUA-13470)

#### Ipomoea barteri

A. Chev.

7604BF96-43B5-51BD-A090-1AEAEA1F7918

##### Distribution

Afro-American

##### Notes

Life Form: therophyte

#### Ipomoea coptica

(L.) Roth ex Roem. & Schult.

71B8F25B-CA22-53E5-971E-8E47FAF5722D

##### Distribution

Paleotropical

##### Notes

Life Form: therophyte; Voucher: Nacoulma 81 (OUA-13445)

#### Ipomoea coscinosperma

Hochst. ex Choisy

60CA5CE7-3202-565F-955D-200FE1B4AAF6

##### Distribution

Pluriregional African

##### Notes

Life Form: therophyte; Voucher: Nacoulma 31 (OUA-13396)

#### Ipomoea eriocarpa

R. Br.

3DBAF2C7-6EE0-595B-BC70-7418508B157F

##### Distribution

Paleotropical

##### Notes

Life Form: therophyte; Voucher: Nacoulma 91 (OUA-13455)

#### Ipomoea heterotricha

Didr.

354B4810-DD32-5EE6-8D85-6FE1B7F10908

##### Distribution

Sudano-Zambesian

##### Notes

Life Form: therophyte; Voucher: Schmidt et al. (FR-0007482)

#### Ipomoea vagans

Baker

1D7A95C2-5EF1-5EC4-A23A-824EFCB27E4F

##### Distribution

Sudanian

##### Notes

Life Form: therophyte; Voucher: Schumann (FR-0083311)

#### Merremia hederacea

(Burm. f.) Hallier f.

62C615A4-763C-5DEE-86CF-528AFD13C7E5

##### Distribution

Paleotropical

##### Notes

Life Form: phanerophyte; Voucher: Nacoulma 4563 (OUA-17132)

#### Merremia kentrocaulos

Rendle

7D8987F5-AE45-5CE2-BF6D-EF519B3849AE

##### Distribution

Paleotropical

##### Notes

Life Form: phanerophyte; Voucher: Nacoulma 60 (OUA-13425)

#### Merremia pinnata

(Hochst. ex Choisy) Hallier f.

D26BF9B1-2B5D-5C3F-BF6D-C435149F8412

##### Distribution

Pluriregional African

##### Notes

Life Form: therophyte; Voucher: Schumann (FR-0083310)

#### Costaceae

255A4212-E6DD-5199-8EEF-DA40A9A2D9CA

#### Costus spectabilis

(Fenzl) K.Schum.

7306EE76-E02D-547D-98F2-4A864F68EF10

##### Distribution

Sudanian

##### Notes

Life Form: geophyte; Voucher: Erpenbach (APPG-29559)

#### Crassulaceae

4A2B95B0-834C-5FDF-856D-8123AFAED710

#### Kalanchoe crenata

(Andrews) Haw.

6D97B3A7-5BDA-5F87-8877-2C51910E8565

##### Distribution

Sudano-Zambesian

##### Notes

Life Form: chamaephyte; Voucher: Nacoulma (APPG-70130)

#### Kalanchoe lanceolata

(Forssk.) Pers.

F6D9F601-E3AA-5B61-B3A0-7E336D654B51

##### Distribution

Sudano-Zambesian

##### Notes

Life Form: chamaephyte

#### Cucurbitaceae

F4F9CE63-2BB0-5ABE-BB2E-C584A077C31A

#### Citrullus lanatus

(Thunb.) Matsum. & Nakai

FE514448-B43F-56A9-8AD8-868A6F3BAF17

##### Distribution

Afrotropical

##### Notes

Life Form: therophyte

#### Cucumis maderaspatanus

L.

8C105DEC-14A3-5293-B28A-D5BDE46F07C2

Cucumis maderaspatanus Mukia maderaspatana (L.) M.Roem.

##### Distribution

Paleotropical

##### Notes

Life Form: hemicryptophyte; Voucher: Nacoulma 32 (OUA-13397)

#### Cucumis melo

L.

337C3768-02D0-5E46-A57C-793C2532BDDF

##### Distribution

Afro-Malagasy

##### Notes

Life Form: phanerophyte; Voucher: Nacoulma 41 (OUA-13406)

#### Cucumis prophetarum

L.

6B96493A-841E-5E6C-AD35-F10E7A3E8995

##### Distribution

Pluriregional African

##### Notes

Life Form: therophyte; Voucher: Nacoulma (OUA-17154)

#### Luffa cylindrica

(L.) M.Roem.

C922A7B6-2DD4-5018-92EA-132D34FBDA2E

##### Distribution

Afro-American

##### Notes

Life Form: therophyte; Voucher: Schmidt et al. (FR-0007582)

#### Trochomeria macrocarpa

(Sond.) Harv.

A8095492-A06D-552A-A789-EA787C96CB0E

##### Distribution

Afrotropical

##### Notes

Life Form: geophyte; Voucher: Nacoulma 59 (OUA-13424)

#### Zehneria hallii

C.Jeffrey

A96A5666-3435-5C35-9673-9B18D10803F8

##### Distribution

Afrotropical

##### Notes

Life Form: phanerophyte; Voucher: Nacoulma 66 (OUA-13431)

#### Cyperaceae

CC7FF97A-1338-5D23-BD00-6C1D78BAAE56

#### Ascolepis protea

Welw.

F1016488-601A-5AF3-9A13-8FCA2AC92C48

##### Distribution

Afrotropical

##### Notes

Life Form: hemicryptophyte

#### Bulbostylis abortiva

(Steud.) C.B.Clarke

16930929-04E1-5894-A258-0FE1FF03A8A2

Abildgaardia abortiva (Steud.) Lye

##### Distribution

Pluriregional African

##### Notes

Life Form: therophyte; Voucher: Zwarg 5 (FR-25005)

#### Bulbostylis cioniana

(Pi.Savi) Lye

D8E25CCE-440E-5BB9-A67F-B3B8BE593D1A

##### Distribution

Pluriregional African

##### Notes

Life Form: therophyte

#### Bulbostylis coleotricha

(Hochst. ex A.Rich.) C.B.Clarke

F697A441-B224-5E30-9CB2-3198B24CC140

Abildgaardia coleotricha (Hochst. ex A.Rich.) Lye

##### Distribution

Sudanian

##### Notes

Life Form: therophyte

#### Bulbostylis densa

(Wall.) Hand.-Mazz.

0E17326A-061D-5C4F-B6F8-776861F40DE4

##### Distribution

Pantropical

##### Notes

Life Form: therophyte

#### Bulbostylis filamentosa

(Vahl) C.B.Clarke

6DC8419F-793A-5FDA-AC2A-7EAD0639F759

Bulbostylis metralis Cherm.

##### Distribution

Afrotropical

##### Notes

Life Form: hemicryptophyte

#### Bulbostylis hispidula

(Vahl) R.W.Haines

691097D4-50A3-501D-B98D-8F2DC433E219

Abildgaardia hispidula (Vahl) Lye|Fimbristylis hispidula (Vahl) Kunth

##### Distribution

Pluriregional African

##### Notes

Life Form: therophyte; Voucher: Mbayngone et al. (FR-7485)

#### Bulbostylis pilosa

(Willd.) Cherm.

8298F8BF-9DF4-5AE9-8493-3ABA8BF3E256

Bulbostylis pilosa Abildgaardia pilosa (Willd.) Nees

##### Distribution

Afrotropical

##### Notes

Life Form: hemicryptophyte

#### Cyperus difformis

L.

64190569-FB94-530E-9057-C70A65E593D9

##### Distribution

Pantropical

##### Notes

Life Form: hemicryptophyte

#### Cyperus esculentus

L.

66F04F8A-3347-589F-8199-6170E9348FE1

##### Distribution

Pantropical

##### Notes

Life Form: geophyte

#### Cyperus haspan

L.

0294F0C9-8CAE-5943-B7B1-FAFA05A94FC3

##### Distribution

Pantropical

##### Notes

Life Form: therophyte; Voucher: Schumann (FR-0083305)

#### Cyperus iria

L.

184A17AA-9785-52DD-9024-294F3457A655

##### Distribution

Pantropical

##### Notes

Life Form: therophyte; Voucher: Schumann (FR-0083303)

#### Cyperus podocarpus

Boeckeler

624733F4-436D-54A0-875F-795C9C7FD669

##### Distribution

Sudano-Zambesian

##### Notes

Life Form: hemicryptophyte

#### Cyperus procerus

Rottb.

CDC457E5-C6C6-5FA6-9665-09DBB9152EE8

##### Distribution

Paleotropical

##### Notes

Life Form: geophyte

#### Cyperus pustulatus

Vahl

009D8C72-DAF0-5AF5-B5EA-1AFC05DE6DD2

##### Distribution

Sudano-Zambesian

##### Notes

Life Form: therophyte; Voucher: Schumann (FR-0083302)

#### Cyperus reduncus

Hochst. ex Boeckeler

04AFB12F-169F-548E-8163-2DC5FF8A35D7

##### Distribution

Pantropical

##### Notes

Life Form: therophyte; Voucher: Schumann (FR-0083301)

#### Cyperus rotundus

L.

EA6D358E-90BE-5631-9DE5-265D5B5BDE52

##### Distribution

Afrotropical

##### Notes

Life Form: geophyte

#### Cyperus tenuiculmis

Boeckeler

E947B3FE-2FE2-5A19-B693-2F92EDA22582

##### Distribution

Pantropical

##### Notes

Life Form: geophyte

#### Diplacrum africanum

(Benth.) C.B.Clarke

DEE6FCC3-6CBF-50C7-A8E9-066F2C715B2B

##### Distribution

Afrotropical

##### Notes

Life Form: therophyte; Voucher: Nacoulma 111 (OUA-13475)

#### Eleocharis atropurpurea

(Retz.) J.Presl & C.Presl

65C861E0-1866-5954-87DE-3715882BACEC

##### Distribution

Pantropical

##### Notes

Life Form: therophyte

#### Eleocharis complanata

Boeckeler

1B5D4427-BA5F-590B-8B10-19F303FB3A1E

##### Distribution

Guineo-Congolian-Sudano-Zambesian

##### Notes

Life Form: therophyte

#### Eleocharis naumanniana

Boeckeler

735B1A33-96FE-5B22-8C80-193CE7B24807

##### Distribution

Guineo-Congolian-Sudano-Zambesian

##### Notes

Life Form: therophyte; Voucher: Schumann (FR-0083298); New species record for Burkina Faso.

#### Fimbristylis dichotoma

(L.) Vahl

449A1A70-64A4-5126-8187-4AF5234FAB7D

##### Distribution

Pantropical

##### Notes

Life Form: hemicryptophyte; Voucher: Nacoulma 4629 (OUA-17197)

#### Fimbristylis ferruginea

(L.) Vahl

2C52F12E-5B0E-5949-838D-A21C690468F7

##### Distribution

Pantropical

##### Notes

Life Form: hemicryptophyte

#### Fimbristylis pilosa

Vahl

FCD044EF-443B-5652-AA93-7C271B3E18BA

##### Distribution

Sudanian

##### Notes

Life Form: hemicryptophyte; Voucher: Schumann (APPG-6708)

#### Fuirena umbellata

Rottb.

DE5017CD-0AF5-5799-B158-3EA324160C03

##### Distribution

Afrotropical

##### Notes

Life Form: geophyte

#### Kyllinga debilis

C.B.Clarke

A9443319-B27D-51FD-9554-56F20BE9B662

##### Distribution

Sudano-Zambesian

##### Notes

Life Form: therophyte

#### Kyllinga erecta

Schumach.

BEBBD805-B9F2-5E0D-97DE-4BE51824A635

##### Distribution

Afro-Malagasy

##### Notes

Life Form: geophyte; Voucher: Zwarg 7 (FR-25007)

#### Kyllinga pumila

Michx.

D8C3DE93-1A02-5779-8A5F-79ACBE815E32

##### Distribution

Afro-American

##### Notes

Life Form: hemicryptophyte

#### Kyllinga squamulata

Vahl

41317293-13F6-54F0-A755-CE08D4FC31DA

##### Distribution

Pantropical

##### Notes

Life Form: therophyte; Voucher: Schumann 188 (FR-0083292)

#### Kyllinga tenuifolia

Steud.

661F80E4-57BD-596A-A1A4-60AC3CC981E3

##### Distribution

Paleotropical

##### Notes

Life Form: hemicryptophyte

#### Lipocarpha chinensis

(Osbeck) J.Kern

5984D69D-61A4-5D97-962D-3324AEDCA977

##### Distribution

Paleotropical

##### Notes

Life Form: therophyte

#### Lipocarpha kernii

(Raymond) Goetgh.

2A764B2A-727C-5437-8AF1-A7E4B4FB18CC

##### Distribution

Paleotropical

##### Notes

Life Form: therophyte; Voucher: Zwarg 21 (FR)

#### Mariscus cylindristachyus

Steud.

956DC1E3-9009-5768-B74E-27CD73D9932E

Mariscus alternifolius Vahl

##### Distribution

Pantropical

##### Notes

Life Form: hemicryptophyte; Voucher: Nacoulma 261 (OUA-17221)

#### Mariscus squarrosus

(L.) C.B.Clarke

B86F81B7-F1C6-5857-8C33-AFB26A117959

##### Distribution

Sudano-Zambesian

##### Notes

Life Form: therophyte; Voucher: Zwarg 32 (FR)

#### Pycreus macrostachyos

(Lam.) J.Raynal

3CDB3042-DD55-557B-8F82-BD986EAA1878

##### Distribution

Pantropical

##### Notes

Life Form: therophyte; Voucher: Katharina Schumann (APPG-6710)

#### Pycreus pumilus

(L.) Nees

C9733D79-9334-5903-ADA6-9F7763D60C89

##### Distribution

Paleotropical

##### Notes

Life Form: therophyte; Voucher: Zwarg 38 (FR)

#### Rhynchospora corymbosa

(L.) Britton

3F9DF081-5244-5DA6-996A-F17941F5CDEE

##### Distribution

Pantropical

##### Notes

Life Form: hemicryptophyte; Voucher: Schmidt et al. (FR-0007428)

#### Schoenoplectiella senegalensis

(Steud.) Lye

6990DA18-88A8-58AE-808B-5558E267AFF3

Schoenoplectiella senegalensis Scirpus jacobii C.E.C. Fisch.

##### Distribution

Paleotropical

##### Notes

Life Form: therophyte; Voucher: Thomas Janßen (APPG-5364)

#### Scleria bulbifera

Hochst. ex A.Rich.

D41ECCCA-48EF-531E-AD71-B3F479932474

##### Distribution

Afro-Malagasy

##### Notes

Life Form: geophyte; Voucher: Schumann (FR-0083289)

#### Scleria melanotricha

Hochst. & A.Rich.

13EAF64B-62D8-588D-9B59-C1E7C55CFACA

##### Distribution

Afrotropical

##### Notes

Life Form: therophyte

#### Scleria pergracilis

(Nees) Kunth

17EBDC2F-42CE-5AFE-843D-22409D40B5CC

##### Distribution

Paleotropical

##### Notes

Life Form: therophyte

#### Scleria sphaerocarpa

(E.A.Rob.) Napper

E7608370-03A5-5FA5-B6EF-F9A53A3935ED

##### Distribution

Sudanian

##### Notes

Life Form: therophyte; Voucher: Zwarg 37 (FR)

#### Scleria tessellata

Willd.

D64F3583-2B46-5593-9F15-417252F42728

##### Distribution

Paleotropical

##### Notes

Life Form: therophyte; Voucher: Schmidt et al. (FR-0007401)

#### Dioscoreaceae

818B8327-783E-504D-B8D7-0BB56C1E728F

#### Dioscorea bulbifera

L.

A47A406D-0CDD-5053-89F6-1A5B7D5BCDCA

##### Distribution

Pantropical

##### Notes

Life Form: geophyte; Voucher: Nacoulma 198 (OUA-13561)

#### Dioscorea dumetorum

(Kunth) Pax

B64150A1-CACD-5042-A8E8-0F717C3E602C

##### Distribution

Pantropical

##### Notes

Life Form: geophyte; Voucher: Nacoulma (APPG-6817)

#### Dioscorea hirtiflora

Benth.

132FF3A4-C9CD-51D3-86AA-AF99B45A1CA1

##### Distribution

Sudano-Zambesian

##### Notes

Life Form: geophyte; Voucher: Nacoulma (OUA-17127)

#### Dioscorea sagittifolia

Pax

BFE6F624-0F17-5CC3-82E2-BCDBC791692F

Dioscorea lecardii De Wild.

##### Distribution

Sudanian

##### Notes

Life Form: geophyte; Voucher: Nacoulma 63 (OUA-13428)

#### Tacca leontopetaloides

(L.) Kuntze

0C6B51D6-CC6C-5650-99B4-A01E80FBF652

##### Distribution

Paleotropical

##### Notes

Life Form: geophyte; Voucher: Georg Zizka (APPG-5658)

#### Dipterocarpaceae

32AE6EEC-32AD-5F03-ABEA-EAAE8225C14A

#### Monotes kerstingii

Gilg

A7CB32CA-6D92-58FF-B06F-85DCC0976319

##### Distribution

Sudanian

##### Notes

Life Form: phanerophyte; Voucher: Thiombiano et al. 3306 (OUA)

#### Droseraceae

CFC4231C-708F-55F8-82AD-2E8346E8F090

#### Drosera indica

L.

9DEDB0F8-ECD1-5F2A-A84B-63C942B31C3F

##### Distribution

Pantropical

##### Notes

Life Form: therophyte; Voucher: Nacoulma 231 (OUA-17070)

#### Ebenaceae

8986F0F8-D1AA-50F7-A192-527FC9E03E61

#### Diospyros mespiliformis

Hochst. ex A.DC.

2F0D663A-DB1E-5921-9FE1-D375836F13F4

##### Distribution

Paleotropical

##### Notes

Life Form: phanerophyte; Voucher: Schmidt et al. (FR-0007391)

#### Eriocaulaceae

C1AD505D-426D-595F-AEE8-1C9A933D193B

#### Eriocaulon afzelianum

Wikstr. ex Körn.

BCCFABD7-CB65-592C-A223-2C2A481FA2D7

##### Distribution

Sudano-Zambesian

##### Notes

Life Form: hydrophyte; Voucher: Schumann (FR-0083279)

#### Eriocaulon nigericum

Meikle

EB089CA8-B539-5B6E-A299-98E2A6174E6C

##### Distribution

Sudano-Zambesian

##### Notes

Life Form: therophyte

#### Eriocaulon plumale

N.E.Br.

449A051C-76E3-53BD-ADC7-7BD8566E2EEC

##### Distribution

Sudano-Zambesian

##### Notes

Life Form: therophyte; Voucher: Schumann (FR-0083278)

#### Euphorbiaceae

5C975E52-E7D1-52AD-BFD8-E9ACDF9FA107

#### Acalypha ciliata

Forssk.

8AF709BC-B214-55F6-B90B-1BCDD7BAB470

##### Distribution

Paleotropical

##### Notes

Life Form: therophyte

#### Acalypha segetalis

Müll.Arg.

BFD2D6CC-6B8F-54A1-9708-692B1E38D093

##### Distribution

Afrotropical

##### Notes

Life Form: therophyte; Voucher: Schumann (FR-0083275)

#### Alchornea cordifolia

(Schumach. & Thonn.) Müll.Arg.

2D955B0C-A56F-5FE5-B01A-1C84A9B2457B

##### Distribution

Afrotropical

##### Notes

Life Form: phanerophyte; Voucher: Nacoulma 4489 (OUA-17077)

#### Caperonia serrata

(Turcz.) C.Presl

FE92BDD1-58B6-52CF-8D96-EA89228FFEAB

##### Distribution

Sudanian

##### Notes

Life Form: therophyte

#### Croton nigritanus

Scott-Elliot

155B9F5B-2F53-5BDD-8ED3-535D526BB3B2

##### Distribution

Guineo-Congolian

##### Notes

Life Form: phanerophyte; Voucher: Nacoulma 197 (OUA-13560)

#### Euphorbia baga

A.Chev.

B47352E9-F4ED-5D2E-A7F6-16A718AFC296

##### Distribution

Afrotropical

##### Notes

Life Form: geophyte; Voucher: Nacoulma (APPG-69981)

#### Euphorbia convolvuloides

Hochst. ex Benth.

01BEA1DD-419C-5C84-A934-8AC037B9A3DE

##### Distribution

Sudanian

##### Notes

Life Form: therophyte

#### Euphorbia hirta

L.

38157904-798C-57F6-AFC2-39429BB4F61D

##### Distribution

Pantropical

##### Notes

Life Form: therophyte

#### Euphorbia poissoni

Pax

A81350FC-087E-5A83-A8F8-3FDFEC9EE157

##### Distribution

Guineo-Congolian

##### Notes

Life Form: phanerophyte; Voucher: Nacoulma (APPG-69984)

#### Euphorbia polycnemoides

Hochst. ex Boiss.

99E288C3-EDF6-596A-A12A-FF2BF2E80B16

##### Distribution

Sudanian

##### Notes

Life Form: therophyte

#### Jatropha atacorensis

A.Chev.

A6DEE096-70BE-5022-B3D9-BFA3E5D2DD5D

##### Distribution

Afrotropical

##### Notes

Life Form: geophyte; Voucher: Nacoulma (APPG-70120) ; New species record for Burkina Faso.

#### Mallotus oppositifolius

(Geiseler) Müll.Arg.

089E1E01-8948-5981-BD11-4B1B22E21927

##### Distribution

Pluriregional African

##### Notes

Life Form: phanerophyte; Voucher: Schmidt et al. (FR-0007604)

#### Tragia laminularis

Müll.Arg.

AB32431C-1E2E-5A4E-8C8A-6E3AD73AD84D

##### Distribution

Sudanian

##### Notes

Life Form: geophyte; Voucher: Nacoulma 4857 (OUA-17347); New species record for Burkina Faso.

#### Tragia senegalensis

Müll.Arg.

66BC07F8-0FC9-5BE9-A59E-523F3A6C512B

##### Distribution

Sudanian

##### Notes

Life Form: phanerophyte; Voucher: Nacoulma (APPG-70400)

#### Fabaceae

3CF1A25C-883A-5F7F-A7F4-692E80ABFE3F

#### Abrus melanospermus

Hassk.

C1C03890-1C0B-50A9-A703-73F205933247

Abrus pulchellus Wall. ex Thwaites

##### Distribution

Afrotropical

##### Notes

Life Form: chamaephyte; Voucher: Nacoulma 137 (OUA-13501)

#### Abrus precatorius

L.

C187D42E-4810-581E-92B0-78F6D260DBA3

##### Distribution

Paleotropical

##### Notes

Life Form: phanerophyte; Voucher: Nacoulma 213 (OUA-13576)

#### Acacia amythethophylla

Steud. ex A. Rich.

3E3AB414-A972-5D6C-9CB2-F76DB72C2A2D

##### Distribution

Sudanian

##### Notes

Life Form: phanerophyte; Voucher: Nacoulma 4528 (OUA-17116)

#### Acacia ataxacantha

DC.

E7998921-8BC9-5710-AC8E-6618DA3D3280

##### Distribution

Sudano-Zambesian

##### Notes

Life Form: phanerophyte

#### Acacia dudgeonii

Craib ex Holland

AB568005-2448-5FBF-B853-85B2C592C6D4

##### Distribution

Sudanian

##### Notes

Life Form: phanerophyte; Voucher: Zwarg 93 (FR)

#### Acacia erythrocalyx

Brenan

F92778F3-9C7F-5270-95F4-BDDEF6316696

##### Distribution

Sudano-Zambesian

##### Notes

Life Form: phanerophyte; Voucher: Nacoulma (APPG-69695)

#### Acacia gerrardii

Benth.

86F72ACB-2AD0-5F55-94DA-920442571DEE

##### Distribution

Sudanian

##### Notes

Life Form: phanerophyte; Voucher: Nacoulma 4518 (OUA-17083)

#### Acacia gourmaensis

A. Chev.

1472BC6D-4699-536A-A6F3-209B0761B2C6

##### Distribution

Sudanian

##### Notes

Life Form: phanerophyte

#### Acacia hockii

De Wild.

4270F39B-3521-57FC-ADCA-1218F3C732F3

##### Distribution

Pluriregional African

##### Notes

Life Form: phanerophyte; Voucher: Zwarg 99 (FR)

#### Acacia macrostachya

Reichenb. ex DC.

5FAB7A06-8FAD-58E7-8BC0-268B09722C73

##### Distribution

Sudano-Zambesian

##### Notes

Life Form: phanerophyte; Voucher: Zwarg 67 (FR)

#### Acacia nilotica

(L.) Willd. ex Delile

CC554A18-A1CB-5284-B07C-9193B42EF27E

##### Distribution

Sudano-Zambesian

##### Notes

Life Form: phanerophyte; Voucher: Nacoulma (APPG-69704)

#### Acacia polyacantha

Willd.

F3EB90FD-B62F-5A23-83E5-D1D76AAAF135

##### Distribution

Pluriregional African

##### Notes

Life Form: phanerophyte

#### Acacia senegal

(L.) Willd.

EFED43FA-DD88-54D5-A760-84A27ED8C7D0

##### Distribution

Sudano-Zambesian

##### Notes

Life Form: phanerophyte

#### Acacia seyal

Delile

344FB357-2911-544F-BFEE-27F520D52BF0

##### Distribution

Sudanian

##### Notes

Life Form: phanerophyte; Voucher: Erpenbach (APPG-26909)

#### Acacia sieberiana

DC.

B9825AA7-A298-53DB-BA1C-1143BBC47A40

##### Distribution

Afrotropical

##### Notes

Life Form: phanerophyte

#### Adenodolichos paniculatus

(Hua) Hutch. & Dalziel

2304ECE7-8F0B-5680-962F-A49EE07B43CA

##### Distribution

Sudanian

##### Notes

Life Form: chamaephyte; Voucher: Nacoulma 167 (OUA-13531)

#### Aeschynomene afraspera

J.Léonard

80094C39-AD4B-54ED-A664-2EA14F75A4CC

##### Distribution

Pluriregional African

##### Notes

Life Form: therophyte

#### Aeschynomene americana

L.

818A3755-B700-5FEC-91A9-6A0E60A9F6AA

##### Distribution

Cosmopolitan

##### Notes

Life Form: therophyte; Voucher: Schmidt et al. (FR-0007492); New species record for Burkina Faso.

#### Aeschynomene indica

L.

A48FDC44-B0E3-5FFD-9722-F2CA77365C4A

##### Distribution

Paleotropical

##### Notes

Life Form: chamaephyte

#### Aeschynomene sensitiva

Sw.

6665424E-12E7-5E9B-94C9-47018A736E14

##### Distribution

Afrotropical

##### Notes

Life Form: therophyte

#### Aeschynomene tambacoundensis

Berhaut

405E5E82-0358-595D-BD0D-41EB4800C95F

##### Distribution

Sudano-Zambesian

##### Notes

Life Form: therophyte

#### Afzelia africana

Sm.

0BF113CB-B809-5A37-8AD9-A20281A936EE

##### Distribution

Sudanian

##### Notes

Life Form: phanerophyte; Voucher: Nacoulma (APPG-69718)

#### Albizia chevalieri

Harms

EFCADDFE-B9C3-59DE-BED8-F97CDC085CCE

##### Distribution

Sudanian

##### Notes

Life Form: phanerophyte; Voucher: Zwarg 113 (FR)

#### Albizia malacophylla

(A.Rich.)Walp.

10A01D81-33DD-55D2-8C94-4D5164FC0E60

##### Distribution

Sudano-Zambesian

##### Notes

Life Form: phanerophyte; Voucher: Nacoulma 138 (OUA-13502)

#### Alysicarpus glumaceus

(Vahl) DC.

1405452E-82B0-5275-A9CC-794BA23D387B

##### Distribution

Pluriregional African

##### Notes

Life Form: therophyte

#### Alysicarpus ovalifolius

(Schumach.) J. Léonard

277F4213-CBBD-546B-86F0-0E8FA3D01BFB

##### Distribution

Paleotropical

##### Notes

Life Form: therophyte; Voucher: Zwarg 115 (FR)

#### Alysicarpus rugosus

(Willd.) DC.

405F692C-6160-5490-ACC7-A9544B13BA80

##### Distribution

Afro-Malagasy

##### Notes

Life Form: therophyte

#### Alysicarpus vaginalis

(L.) DC.

8A5DF686-A3D1-58E8-A350-D3922848623D

##### Distribution

Paleotropical

##### Notes

Life Form: therophyte; Voucher: Nacoulma 181 (OUA-13544); New species record for Burkina Faso.

#### Andira inermis

(Wright) DC.

C636F886-A847-5FF3-9DA1-2ECEF96B731A

##### Distribution

Sudanian

##### Notes

Life Form: phanerophyte

#### Bobgunnia madagascariensis

(Desv.)J.H.Kirkbr. & Wiersema

548DE0BC-61DE-5FF8-931E-B7237AB76844

Swartzia madagascariensis Desv.

##### Distribution

Afro-Malagasy

##### Notes

Life Form: phanerophyte; Voucher: Nacoulma (APPG-69771)

#### Burkea africana

Hook.

F6CD9312-4D03-5082-8A44-4C007AA5E1FE

##### Distribution

Afrotropical

##### Notes

Life Form: phanerophyte; Voucher: Zwarg 81 (FR)

#### Cajanus kerstingii

Harms

7C540449-587B-53E1-81C2-996274F948F3

##### Distribution

Sudano-Zambesian

##### Notes

Life Form: chamaephyte

#### Cassia absus

L.

1EA5B4E2-1F6B-5598-BED9-52F7FAECA97E

##### Distribution

Paleotropical

##### Notes

Life Form: chamaephyte; Voucher: Zwarg 23 (FR)

#### Cassia mimosoides

L.

D7DFA28A-377C-54F0-9FB8-C4DEF022A149

Cassia mimosoides Chamaecrista pratensis (R.Vig.)Du Puy

##### Distribution

Paleotropical

##### Notes

Life Form: therophyte; Voucher: Zwarg 13 (FR)

#### Cassia nigricans

Vahl

24571310-66E5-5320-A0B9-ED7781F020A8

##### Distribution

Paleotropical

##### Notes

Life Form: therophyte

#### Cassia obtusifolia

L.

7EA7B8B6-ABDA-58B0-BAA6-D0A00C825503

##### Distribution

Pantropical (introduced)

##### Notes

Life Form: therophyte

#### Cassia sieberiana

DC.

6FD8FE6F-EB13-5A13-86EE-AD86F82683DD

##### Distribution

Sudanian

##### Notes

Life Form: phanerophyte; Voucher: Zwarg 56 (FR)

#### Cassia singueana

Delile

C175EEC3-65CB-5F84-A100-8E7AD2DD68A7

##### Distribution

Sudanian

##### Notes

Life Form: phanerophyte

#### Crotalaria barkae

Schweinf.

A49C402A-3908-5D80-8993-EACEFDFC551F

##### Distribution

Sudanian

##### Notes

Life Form: chamaephyte; Voucher: Nacoulma 29 (OUA-13394)

#### Crotalaria calycina

Schrank

E7A4DD6D-4646-509C-87CF-515086638B17

##### Distribution

Paleotropical

##### Notes

Life Form: therophyte; Voucher: Nacoulma 89 (OUA-13453)

#### Crotalaria cephalotes

A.Rich.

EB35654B-BA89-5328-BEA2-BE3AA71A9569

##### Distribution

Pantropical

##### Notes

Life Form: therophyte

#### Crotalaria comosa

Baker

88566F5C-7F1A-575C-9B62-D7E7C50BB628

##### Distribution

Guineo-Congolian-Sudano-Zambesian

##### Notes

Life Form: therophyte

#### Crotalaria glauca

Willd.

3B1AF879-A349-54C1-9438-455054F1074D

##### Distribution

Sudanian

##### Notes

Life Form: therophyte; Voucher: Nacoulma 55 (OUA-13420)

#### Crotalaria goreensis

Guill. & Perr

0CF54218-F05A-5D2E-87D1-BA7A94BAA1D0

##### Distribution

Pluriregional African

##### Notes

Life Form: therophyte; Voucher: Zwarg 134 (FR)

#### Crotalaria hyssopifolia

Klotzsch

FDAC65AF-63BD-55AB-A3F3-51016F81B0A3

##### Distribution

Guineo-Congolian-Sudano-Zambesian

##### Notes

Life Form: chamaephyte; Voucher: Schumann (FR-0083259)

#### Crotalaria lachnophora

A.Rich.

6DA65AD5-F980-5D43-850B-1DC7852D9D7F

##### Distribution

Pluriregional African

##### Notes

Life Form: therophyte; Voucher: Nacoulma 4890 (OUA-17359); New species record for Burkina Faso.

#### Crotalaria lathyroides

Guill. & Perr.

3EF928A6-0A92-5E2E-9FE1-592B41FEFDD3

##### Distribution

Sudanian

##### Notes

Life Form: therophyte

#### Crotalaria leprieurii

Guill. & Perr.

6DB2B6A6-F38F-5830-839C-1226F4D46488

##### Distribution

Sudano-Zambesian

##### Notes

Life Form: therophyte; Voucher: Zwarg 35 (FR)

#### Crotalaria macrocalyx

Benth.

C1F1AFDC-5488-53A1-99E8-25E99147D7D8

##### Distribution

Sudanian

##### Notes

Life Form: therophyte; Voucher: Nacoulma 7 (OUA-13377)

#### Crotalaria microcarpa

Hochst. ex Benth.

247FD1BE-5F98-5F15-BA9A-CC2FBE8A6CBB

##### Distribution

Sudano-Zambesian

##### Notes

Life Form: therophyte

#### Crotalaria naragutensis

Hutch.

34E97865-912D-56CF-B2E1-AA0D6512D20E

##### Distribution

Sudanian

##### Notes

Life Form: therophyte; Voucher: Schmidt et al. (FR-0007647)

#### Crotalaria pallida

Aiton

0F1379EC-3E66-58C7-979A-7E7ED16CF1E9

##### Distribution

Pantropical

##### Notes

Life Form: therophyte; Voucher: Nacoulma 77 (OUA-13441)

#### Crotalaria podocarpa

DC.

BB92DC51-9914-5CFF-AD92-F15028C5B4DD

##### Distribution

Sudano-Zambesian

##### Notes

Life Form: therophyte

#### Crotalaria retusa

L.

78B9B3E6-5316-5742-AC01-61E2C2AC7068

##### Distribution

Pantropical

##### Notes

Life Form: chamaephyte; Voucher: Nacoulma (APPG-69877)

#### Crotalaria senegalensis

(Pers.) Bacle ex DC.

2DC0292D-D1BB-5F85-8F69-A34592181CAA

##### Distribution

Sudano-Zambesian

##### Notes

Life Form: therophyte; Voucher: Nacoulma 163 (OUA-13527)

#### Daniellia oliveri

(Rolfe)Hutch. & Dalziel

1591498F-A1F5-5B12-BB21-5A864C462F61

##### Distribution

Sudano-Zambesian

##### Notes

Life Form: phanerophyte

#### Desmodium adscendens

(Sw.) DC.

D88EB9F9-F688-59DC-AAC6-8B334EA205CF

##### Distribution

Paleotropical (introduced)

##### Notes

Life Form: chamaephyte; Voucher: Zwarg 50 (FR)

#### Desmodium gangeticum

(L.)DC.

B328435A-C45E-5B86-9A60-9C1FCA53AC7B

##### Distribution

Paleotropical

##### Notes

Life Form: chamaephyte; Voucher: Nacoulma 144 (OUA-13508)

#### Desmodium hirtum

Guill. & Perr.

4A4FC5E1-9CE3-5949-AB92-B80854359C99

##### Distribution

Sudano-Zambesian

##### Notes

Life Form: therophyte; Voucher: Nacoulma 221 (OUA-13583)

#### Desmodium ospriostreblum

Steud. ex A. Rich.

A9404532-CFAB-5023-AD3E-27BE2FD8065D

##### Distribution

Sudanian

##### Notes

Life Form: therophyte; Voucher: Nacoulma 72 (OUA-13436)

#### Desmodium ramosissimum

G.Don

7340EBF9-5EE3-54E4-95F7-957A9541DCB3

##### Distribution

Afro-Malagasy

##### Notes

Life Form: chamaephyte; Voucher: Nacoulma 5310 (OUA-17810); New species record for Burkina Faso.

#### Desmodium setigerum

(E.Mey.)Harv.

A58A824F-BD47-5E7D-A340-A3A245924906

##### Distribution

Sudano-Zambesian

##### Notes

Life Form: therophyte; Voucher: Nacoulma 5 (OUA-13375)

#### Desmodium velutinum

(Willd.) DC.

185955F4-70EC-5D38-907A-23EB498E126B

##### Distribution

Pantropical

##### Notes

Life Form: chamaephyte; Voucher: Schmidt et al. (FR-0007423)

#### Detarium microcarpum

Guill. & Perr.

FA4159AA-0A48-5EB4-BBCA-1C05E43A4016

##### Distribution

Sudanian

##### Notes

Life Form: phanerophyte; Voucher: Zizka (APPG-5662)

#### Dichrostachys cinerea

(L.) Wight & Arn.

0AA5C34D-A497-535C-8092-D0E42912B344

##### Distribution

Pantropical

##### Notes

Life Form: phanerophyte; Voucher: Zwarg 130 (FR)

#### Entada africana

Guill. & Perr.

D9157111-ED89-5805-B891-8B6E7CDB2222

##### Distribution

Sudanian

##### Notes

Life Form: phanerophyte; Voucher: Zwarg 89 (FR)

#### Eriosema psoraloides

(Lam.) G.Don

34B3464F-2E6E-51B0-87B6-B179018025E6

##### Distribution

Pantropical

##### Notes

Life Form: chamaephyte

#### Erythrina senegalensis

DC.

7621046D-954E-5425-8F02-838DFAE2DF83

##### Distribution

Guineo-Congolian-Sudano-Zambesian

##### Notes

Life Form: phanerophyte; Voucher: Nacoulma (APPG-69951)

#### Indigofera bracteolata

DC.

D9DB7C6F-AA07-5688-A394-6FB90BD31B35

##### Distribution

Sudanian

##### Notes

Life Form: chamaephyte; Voucher: Nacoulma 78 (OUA-13442)

#### Indigofera congolensis

De Wild. & T.Durand

F79148C2-751E-5475-8F2E-BAA72F741E76

##### Distribution

Afrotropical

##### Notes

Life Form: therophyte; Voucher: Zwarg 26 (FR)

#### Indigofera dendroides

Jacq.

35A6A155-65AD-5254-951D-730FF20DF89D

##### Distribution

Afrotropical

##### Notes

Life Form: therophyte; Voucher: Nacoulma 30 (OUA-13395)

#### Indigofera garckeana

Vatke

2FB35B44-C848-5E85-8B76-29A671E6B5A4

##### Distribution

Sudanian

##### Notes

Life Form: therophyte; Voucher: Nacoulma (APPG-70072); New species record for Burkina Faso.

#### Indigofera geminata

Baker

6D3016B9-3AAE-56E5-BEA3-6AEA100FD8F6

##### Distribution

Sudanian

##### Notes

Life Form: therophyte; Voucher: Zwarg 11 (FR)

#### Indigofera hirsuta

L.

FBE74FFD-7110-5F59-9FA6-86B2A5C93724

##### Distribution

Pantropical

##### Notes

Life Form: therophyte; Voucher: Nacoulma 4635 (OUA-17191)

#### Indigofera kerstingii

Harms

D86A6983-780A-565C-BE40-A940072CEED0

##### Distribution

Guineo-Congolian

##### Notes

Life Form: therophyte; Voucher: Nacoulma 236 (OUA-17224)

#### Indigofera leprieurii

Baker f.

D1CD7A59-D3F4-5411-BC8F-354B87F6779C

##### Distribution

Sudanian

##### Notes

Life Form: therophyte

#### Indigofera macrocalyx

Guill. & Perr.

B7AC5B52-9517-58E2-99F4-E603FA954F59

##### Distribution

Sudanian

##### Notes

Life Form: therophyte; Voucher: Schmidt et al. (FR-0007484)

#### Indigofera microcarpa

Desv.

6AE80ED8-4859-58D3-8F88-C70A6AD5C134

##### Distribution

Afro-Malagasy (introduced)

##### Notes

Life Form: therophyte

#### Indigofera nummulariifolia

(L.)Alston

17793B09-45EE-59BE-9A78-6975B1A83F2B

##### Distribution

Pluriregional African

##### Notes

Life Form: therophyte; Voucher: Schumann (FR-0083355)

#### Indigofera paniculata

Pers.

5ED35D21-BA6F-5542-8DA3-2F2277DC106F

##### Distribution

Afrotropical

##### Notes

Life Form: therophyte; Voucher: Schmidt et al. (FR-0007381)

#### Indigofera polysphaera

Baker

51CA1B49-EE78-5DB0-92FC-56C41574C289

##### Distribution

Sudanian

##### Notes

Life Form: phanerophyte; Voucher: Schumann (FR-0083243)

#### Indigofera pulchra

Willd.

D90287E7-8B18-5A13-9CA6-1CAA4846A5BD

##### Distribution

Sudano-Zambesian

##### Notes

Life Form: therophyte

#### Indigofera secundiflora

Poir.

A13AD0CF-BE96-5339-A2B0-628BC44DDE9F

##### Distribution

Paleotropical

##### Notes

Life Form: chamaephyte

#### Indigofera senegalensis

Lam.

1E734E6F-6760-55C7-B441-87B065553B28

##### Distribution

Sudanian

##### Notes

Life Form: therophyte; Voucher: Nacoulma 42 (OUA-13407)

#### Indigofera simplicifolia

Lam.

694D8D4F-9EDC-5FD1-A6CB-845E37CE8721

##### Distribution

Sudano-Zambesian

##### Notes

Life Form: therophyte; Voucher: Nacoulma 43 (OUA-13408)

#### Indigofera stenophylla

Guill. & Perr.

05313F4B-0004-5D8A-A2DB-B3E21F9FF875

##### Distribution

Sudanian

##### Notes

Life Form: therophyte; Voucher: Zwarg 59 (FR)

#### Indigofera tinctoria

L.

C6BF4B0F-44A4-5040-8C0E-933D6105A58C

##### Distribution

Paleotropical

##### Notes

Life Form: chamaephyte; Voucher: Schumann (FR-0083240)

#### Indigofera trita

L.f.

9E6451BE-647F-5030-8534-2BF23B53764B

Indigofera subulata Vahl ex Poir.

##### Distribution

Afrotropical

##### Notes

Life Form: chamaephyte

#### Isoberlinia doka

Craib & Stapf

D637DCB1-D8CA-5B33-AF49-F84EE3387681

##### Distribution

Sudanian

##### Notes

Life Form: phanerophyte; Voucher: Zwarg 128 (FR)

#### Macrotyloma biflorum

(Schumach. & Thonn.) Hepper

1F7A7386-EAB4-55B3-BABB-F400C4DF8C39

##### Distribution

Pluriregional African

##### Notes

Life Form: therophyte; Voucher: Nacoulma 68 (OUA-13432)

#### Melliniella micrantha

Harms

3C4961F2-EE74-5DC4-80D2-96681466DDA7

##### Distribution

Sudanian

##### Notes

Life Form: therophyte; Voucher: Zwarg 24 (FR)

#### Mimosa pigra

L.

E4190C4C-4253-5DF9-93E6-EA3633E863FE

##### Distribution

Pantropical

##### Notes

Life Form: phanerophyte; Voucher: Nacoulma (APPG-70218)

#### Neptunia oleracea

Lour.

4B03C7A5-373E-55A2-8F42-A44ECF4EEE43

##### Distribution

Pantropical

##### Notes

Life Form: hydrophyte; Voucher: Georg Zizka (APPG-3861)

#### Parkia biglobosa

(Jacq.)G.Don

46D6BE52-DAA1-51D7-8695-361570BCD5FF

##### Distribution

Paleotropical

##### Notes

Life Form: phanerophyte; Voucher: Schumann (APPG-2030)

#### Pericopsis laxiflora

(Baker)Meeuwen

ADCD5B8F-3A19-5260-B666-5637DF88074C

##### Distribution

Sudanian

##### Notes

Life Form: phanerophyte; Voucher: Nacoulma 10 (OUA-13380)

#### Philenoptera laxiflora

(Guill. & Perr.)Roberty

BD1D7BA2-A5AC-51A0-B236-A26F1A5B3029

##### Distribution

Pluriregional African

##### Notes

Life Form: phanerophyte; Voucher: Schumann (APPG-1663)

#### Piliostigma reticulatum

(DC.)Hochst.

DFB41813-39F8-5338-A5E0-070F3AF8BEA1

##### Distribution

Sudanian

##### Notes

Life Form: phanerophyte

#### Piliostigma thonningii

(Schum.)Milne-Redh.

1742EBA4-EEF5-55CE-BE71-3EFD2EFDF774

##### Distribution

Afrotropical

##### Notes

Life Form: phanerophyte; Voucher: Schumann (APPG-2124)

#### Prosopis africana

(Guill. & Perr.)Taub.

7C0DD16C-F4CF-52C6-BC25-3AC106F4BD6E

##### Distribution

Sudanian

##### Notes

Life Form: phanerophyte; Voucher: Zwarg 96 (FR)

#### Pterocarpus erinaceus

Poir.

3E0E1946-53F6-598A-819C-8D9D5900F4BA

##### Distribution

Sudano-Zambesian

##### Notes

Life Form: phanerophyte; Voucher: Zwarg 133 (FR)

#### Rhynchosia densiflora

(Roth)DC.

4F10B229-3202-5DEF-B945-7D47DF69D8D9

##### Distribution

Paleotropical

##### Notes

Life Form: therophyte; Voucher: Nacoulma 188 (OUA-13551)

#### Rhynchosia minima

(L.) DC.

BFB1A154-F9C7-5E40-A3AE-42536E89155D

##### Distribution

Pantropical

##### Notes

Life Form: therophyte; Voucher: Nacoulma 56 (OUA-13421)

#### Rhynchosia nyasica

Baker

4563355D-5DA3-513E-B51F-AB06675C983C

##### Distribution

Sudano-Zambesian

##### Notes

Life Form: chamaephyte; Voucher: Schumann (FR-0083236)

#### Rhynchosia sublobata

(Schum.)Meikle

587FAAD2-9774-5681-A836-C37AA9A416A1

##### Distribution

Pluriregional African

##### Notes

Life Form: chamaephyte

#### Sesbania sesban

(L.) Fawc. & Rendle

A353BC49-626D-5161-93AC-F97EF31347C0

##### Distribution

Paleotropical

##### Notes

Life Form: phanerophyte

#### Stylosanthes erecta

P.Beauv.

FFA0511F-8848-513A-86B1-B208D8A1C570

##### Distribution

Afrotropical

##### Notes

Life Form: chamaephyte; Voucher: Zwarg 114 (FR)

#### Tamarindus indica

L.

FC196E77-AF5B-5A95-B7BE-1CD144F710AA

##### Distribution

Pantropical

##### Notes

Life Form: phanerophyte; Voucher: Schumann (APPG-2554)

#### Tephrosia bracteolata

Guill. & Perr.

4C1D6782-A4F5-53FD-8CB9-2B425CE130B1

##### Distribution

Sudano-Zambesian

##### Notes

Life Form: therophyte; Voucher: Zwarg 63 (FR)

#### Tephrosia elegans

Schumach.

B6EF871C-93E1-50A8-910E-54B7BEABFC0C

##### Distribution

Sudano-Zambesian

##### Notes

Life Form: therophyte; Voucher: Nacoulma 53 (OUA-13418)

#### Tephrosia gracilipes

Guill. & Perr.

FB0B83D3-CBE0-572D-AF98-A2A46F3D4BA3

##### Distribution

Sudanian

##### Notes

Life Form: therophyte

#### Tephrosia humilis

Guill. & Perr.

ED7DD712-B0C2-5732-B523-5F6EEE38615B

##### Distribution

Sudanian

##### Notes

Life Form: therophyte

#### Tephrosia linearis

(Willd.)Pers.

E3E30083-A41F-5661-A499-A9FDFC459424

##### Distribution

Afro-Malagasy

##### Notes

Life Form: therophyte; Voucher: Nacoulma 103 (OUA-13467)

#### Tephrosia nana

Schweinf.

BE4C459B-DCFC-5AE7-B1DE-3F52D5CDB000

##### Distribution

Sudano-Zambesian

##### Notes

Life Form: therophyte; Voucher: Schmidt et al. (FR-0007624)

#### Tephrosia pedicellata

Baker

E146AE0F-97AC-5F4F-BE92-937B184AD788

##### Distribution

Sudanian

##### Notes

Life Form: therophyte; Voucher: Georg Zizka (APPG-3897)

#### Tephrosia platycarpa

Guill. & Perr.

7749B5F9-7D60-582B-9856-97D1A3944847

##### Distribution

Sudanian

##### Notes

Life Form: therophyte; Voucher: Zwarg 74 (FR)

#### Teramnus labialis

(L.f.)Spreng.

69306652-DCF2-579B-81E2-A1C6368D7DBC

##### Distribution

Pantropical

##### Notes

Life Form: therophyte; Voucher: Schmidt et al. (FR-0007466)

#### Uraria picta

(Jacq.) Desv.

94C76559-D2AF-5E19-B8BF-EA92CD394E1F

##### Distribution

Paleotropical

##### Notes

Life Form: therophyte; Voucher: Nacoulma (APPG-70403)

#### Vigna filicaulis

Hepper

EA0BB3CC-7611-50CC-8BEA-DE93EDDB2E22

##### Distribution

Sudano-Zambesian

##### Notes

Life Form: therophyte; Voucher: Schumann (FR-0083229)

#### Vigna gracilis

(Guill. & Perr.) Hook.f.

3A3060B0-F681-5D80-BF2B-8F80FD297550

##### Distribution

Pluriregional African

##### Notes

Life Form: therophyte; Voucher: Nacoulma 4511 (OUA-17090)

#### Vigna heterophylla

A.Rich.

E21F204A-9138-5486-9EC2-029CA3618395

##### Distribution

Sudano-Zambesian

##### Notes

Life Form: therophyte; Voucher: Nacoulma 37 (OUA-13402)

#### Vigna nigritia

Hook.f.

918A7E52-7853-52E9-8127-DE12A179627C

##### Distribution

Pluriregional African

##### Notes

Life Form: therophyte; Voucher: Nacoulma 4526 (OUA-17118); New species record for Burkina Faso.

#### Vigna racemosa

(G.Don)Hutch. & Dalziel

C4ED61EC-6CFB-5E6E-AEA2-CFA94FDF39CC

##### Distribution

Sudano-Zambesian

##### Notes

Life Form: therophyte; Voucher: Nacoulma 218 (OUA-13580)

#### Vigna reticulata

Hook.f.

63BF31C3-55EC-5E78-9DB2-AA287EB875EB

##### Distribution

Sudano-Zambesian

##### Notes

Life Form: therophyte

#### Vigna vexillata

(L.) A.Rich.

05AAACD3-F5B8-591B-A093-F1239593DADF

##### Distribution

Afro-Malagasy

##### Notes

Life Form: therophyte; Voucher: Schmidt et al. (FR-0007386)

#### Xeroderris stuhlmannii

(Taub.) Mendonça & E.C. Sousa

62AD84D3-EF62-53D0-BFB4-1661F81C8480

Aganope stuhlmannii (Taub.) Adema

##### Distribution

Sudano-Zambesian

##### Notes

Life Form: phanerophyte; Voucher: Nacoulma (APPG-70437)

#### Zornia glochidiata

DC.

8F0AE95A-80E7-5E35-9CE0-4B7A6CA4F93A

##### Distribution

Afrotropical

##### Notes

Life Form: therophyte; Voucher: Zwarg 41 (FR)

#### Gentianaceae

2A84260C-092B-5D6F-B202-8601C6CA1C8C

#### Canscora diffusa

(Vahl) R.Br. ex Roem. & Schult.

3E8BC71A-35D9-5354-B1BB-DB6140479A47

##### Distribution

Sudano-Zambesian

##### Notes

Life Form: therophyte; Voucher: Nacoulma 105 (OUA-13469)

#### Faroa pusilla

Baker

476DFC99-9A5F-59B9-A6EE-122D0E16B87E

##### Distribution

Sudano-Zambesian

##### Notes

Life Form: therophyte; Voucher: Nacoulma 4535 (OUA-17241)

#### Hydrocharitaceae

96EB67C2-C475-59C1-8B7D-0F85E5EB6216

#### Ottelia ulvifolia

(Planch.) Walp.

D72F254A-CBD0-5A09-9AC7-F10347D2E68F

##### Distribution

Afro-Malagasy

##### Notes

Life Form: therophyte; Voucher: Schmidt et al. (FR-0007433)

#### Hydroleaceae

F781F76E-0B5C-5662-9245-85888F6B1DD7

#### Hydrolea macrosepala

A.W.Benn.

E2DF9855-78B8-50D8-B93F-ADE8C8FC07F9

##### Distribution

Sudano-Zambesian

##### Notes

Life Form: therophyte

#### Hypericaceae

767B078E-39A3-503C-8C9A-F7005F4ACF2B

#### Psorospermum glaberrimum

Hochr.

BBB32BC1-E2DB-5308-A697-ECE75ADD7646

##### Distribution

Sudanian

##### Notes

Life Form: phanerophyte

#### Hypoxidaceae

DDA2F8A6-24BB-5770-8CE3-7EE20CDAAFA0

#### Curculigo pilosa

(Schumach. & Thonn.) Engl.

D2FF10A8-1DD0-5AFD-A8B6-8A65AB1453D3

##### Distribution

Afro-Malagasy

##### Notes

Life Form: geophyte; Voucher: Nacoulma (APPG-69892)

#### Iridaceae

DCE3F98D-1054-57F5-AF7B-47BC7E58243A

#### Gladiolus dalenii

Van Geel

5B49D2E8-EC42-5296-B6BF-586163C730AA

##### Distribution

Sudano-Zambesian

##### Notes

Life Form: geophyte; Voucher: Nacoulma 26 (OUA-13392)

#### Gladiolus gregarius

Welw. ex Baker

08D74780-56D8-5AD2-B8C2-CC0CB8C74D4A

Gladiolus klattianus Hutch.

##### Distribution

Sudano-Zambesian

##### Notes

Life Form: geophyte; Voucher: Nacoulma 69 (OUA-13433)

#### Gladiolus unguiculatus

Baker

36ECC486-65FF-5CDC-A00B-4FA9CE539D86

##### Distribution

Pluriregional African

##### Notes

Life Form: geophyte; Voucher: Nacoulma (APPG-70039); New species record for Burkina Faso.

#### Isoetaceae

F18514C3-C1B6-51DF-81B0-61BF80774993

#### Isoetes nigritiana

A. Braun

61596EB1-E48B-5D87-92FC-FA6FB0650AE6

##### Distribution

Sudano-Zambesian

##### Notes

Life Form: hemicryptophyte

#### Lamiaceae

5E64D301-C493-52E8-92F4-251CE7D214BC

#### Aeollanthus pubescens

Benth.

FAE1048D-5FF9-5F6B-8410-BC8B06808AC7

##### Distribution

Pluriregional African

##### Notes

Life Form: therophyte; Voucher: Schumann (FR-0083218)

#### Clerodendrum capitatum

(Willd.) Schumach. & Thonn.

0FA6A209-28CB-53EF-83A0-A7C378736CF4

##### Distribution

Sudanian

##### Notes

Life Form: phanerophyte; Voucher: Zizka (APPG-5665)

#### Gmelina arborea

Roxb.

7DA112C3-EFE0-5C4D-8979-31273A34A058

##### Distribution

Cultivated

##### Notes

Life Form: phanerophyte

#### Hoslundia opposita

Vahl

22346AE9-6693-598A-A4B8-CF4C3B1C75E4

##### Distribution

Afro-Malagasy

##### Notes

Life Form: phanerophyte; Voucher: Nacoulma 238 (OUA-17236)

#### Hyptis spicigera

Lam.

8ADB3A31-E937-5ED3-A8FC-32789A646515

##### Distribution

Pantropical (introduced)

##### Notes

Life Form: therophyte; Voucher: Zwarg 18 (FR)

#### Hyptis suaveolens

(L.) Poit.

AE4207C1-F53E-5549-A916-3E79A3874CCC

##### Distribution

Pantropical (introduced)

##### Notes

Life Form: therophyte

#### Lantana ukambensis

(Vatke) Verdc.

FB9D2E91-82A6-5899-8E76-164096465383

##### Distribution

Sudanian

##### Notes

Life Form: geophyte; Voucher: Nacoulma (APPG-70160)

#### Leonotis nepetifolia

(L.) R.Br.

E97C4F72-DC42-5A9F-A2A0-71A1E884362C

##### Distribution

Pluriregional African

##### Notes

Life Form: therophyte; Voucher: Schmidt et al. (FR-0007627)

#### Leucas martinicensis

(Jacq.) R.Br.

F953EC96-7B2B-5235-981F-802DAAEA646F

##### Distribution

Pantropical

##### Notes

Life Form: therophyte

#### Lippia chevalieri

Moldenke

58315E38-FADE-5380-AE18-368E10C2BD39

##### Distribution

Sudanian

##### Notes

Life Form: geophyte; Voucher: Nacoulma (APPG-70181)

#### Ocimum americanum

L.

A85C3F45-E335-5844-9748-10620161414B

##### Distribution

Pantropical

##### Notes

Life Form: therophyte; Voucher: Schumann (FR-0083214)

#### Platostoma africanum

P.Beauv.

A1F66456-E134-5533-B8E7-3DF67423CDE1

##### Distribution

Sudanian

##### Notes

Life Form: therophyte; Voucher: Zwarg 84 (FR)

#### Plectranthus gracillimus

(T.C.E.Fr.) Hutch. & Dandy

54EAECA2-773E-5189-ADBB-4BD2925CAADA

Plectranthus gracillimus Englerastrum gracillimum T.C.E.Fr.

##### Distribution

Sudanian

##### Notes

Life Form: therophyte

#### Plectranthus monostachyus

(P.Beauv.) B.J.Pollard

0E11B92B-C670-5B3B-818D-85B531F17D19

Solenostemon monostachyus (P.Beauv.) Briq.

##### Distribution

Afrotropical

##### Notes

Life Form: therophyte; Voucher: Schumann (FR-0083212)

#### Tinnea barteri

Gürke

CDFC7068-493C-53B1-A13B-6592C5F3A492

##### Distribution

Sudanian

##### Notes

Life Form: chamaephyte; Voucher: Nacoulma 80 (OUA-13444)

#### Vitex chrysocarpa

Planch.

5D88C1ED-6C09-58E9-9280-2DD4B8675B08

##### Distribution

Sudanian

##### Notes

Life Form: phanerophyte; Voucher: Nacoulma 47 (OUA-13412)

#### Vitex doniana

Sweet

1B4DA81B-71B0-5219-B3E6-8F9B665E6037

##### Distribution

Afrotropical

##### Notes

Life Form: phanerophyte; Voucher: Nacoulma (APPG-70433)

#### Vitex madiensis

Oliv.

5AFE1811-1017-506F-8C4C-1689A46AEAB0

##### Distribution

Sudanian

##### Notes

Life Form: phanerophyte; Voucher: Katharina Schumann (APPG-2848)

#### Lentibulariaceae

C5BB54D3-E442-5973-BA09-205F4D025F5B

#### Utricularia inflexa

Forssk.

1527E2E2-5B6A-553D-B603-31F3871A5288

##### Distribution

Paleotropical

##### Notes

Life Form: therophyte; Voucher: Nacoulma 4488 (OUA-17066)

#### Utricularia stellaris

L.f.

C3F821DC-56D2-55FF-8D8B-D4D3D05606EC

##### Distribution

Paleotropical

##### Notes

Life Form: hydrophyte; Voucher: Zizka (APPG-3900)

#### Linderniaceae

826528B5-6677-50D5-B4D9-907BAAACD05A

#### Lindernia exilis

Philcox

A81FE126-FB9B-562A-958E-0D1E4AFBF5C8

Ilysanthes gracilis Skan

##### Distribution

Sudanian

##### Notes

Life Form: therophyte

#### Loganiaceae

AA581BEC-C28D-5399-AA6E-1ECE7C5DCAD6

#### Strychnos innocua

Delile

947BFF94-6C38-5EF3-8772-CF91AF0A5E5B

##### Distribution

Sudano-Zambesian

##### Notes

Life Form: phanerophyte; Voucher: Nacoulma (APPG-70379)

#### Strychnos spinosa

Lam.

F7162EF7-2C40-5E5C-A834-44F3572CB580

##### Distribution

Afro-Malagasy

##### Notes

Life Form: phanerophyte; Voucher: Katharina Schumann (APPG-2530)

#### Loranthaceae

1664674A-4D7C-5AFA-B13A-E07BAEFB7498

#### Agelanthus dodoneifolius

(DC.) Polhill & Wiens

BEC5F62E-F059-5323-834F-0E7CB946B15C

Tapinanthus dodoneifolius (DC.) Danser

##### Distribution

Sudanian

##### Notes

Life Form: epiphyte

#### Tapinanthus bangwensis

(Engl. & K.Krause) Danser

63FB4D2F-304D-58DB-88B8-D7B7CEBD2072

##### Distribution

Guineo-Congolian

##### Notes

Life Form: epiphyte; Voucher: Nacoulma (APPG-6855)

#### Tapinanthus globiferus

(A.Rich.) Tiegh.

B62A3B31-1017-5B82-867F-D25E3717C6DC

##### Distribution

Afrotropical

##### Notes

Life Form: phanerophyte

#### Lythraceae

CEC102C1-C5A6-506E-A3F4-E6E051C1C784

#### Ammannia auriculata

Willd.

CB1B23BD-5959-5915-AFEF-7F7405694EB2

##### Distribution

Pantropical

##### Notes

Life Form: therophyte; Voucher: Schmidt et al. (FR-0007399)

#### Ammannia baccifera

L.

B8E11250-0669-5BF6-A4CF-1EF5AAFC6D93

##### Distribution

Paleotropical

##### Notes

Life Form: therophyte; Voucher: Nacoulma 4485 (OUA-17063)

#### Ammannia senegalensis

Lam.

58A9892D-C35E-51DB-BFDD-7DE03C322AE5

##### Distribution

Sudanian

##### Notes

Life Form: therophyte

#### Rotala stagnina

Hiern

47E5D88D-DB7A-5136-8A8A-2D42CECFCCC2

##### Distribution

Sudano-Zambesian

##### Notes

Life Form: hemicryptophyte; Voucher: Nacoulma 4486 (OUA-17064)

#### Malvaceae

9DE6151A-DCF7-5757-8D17-F575B7441190

#### Abutilon pannosum

(G.Forst.) Schltdl.

3018F9F8-8DF4-5F3F-8FA9-3D06E358C0A4

##### Distribution

Paleotropical

##### Notes

Life Form: chamaephyte; Voucher: Schumann (FR-0083206)

#### Abutilon ramosum

(Cav.) Guill. & Perr.

0D56EF36-44FD-5522-AE3A-2E346A1377C8

##### Distribution

Sudano-Zambesian

##### Notes

Life Form: chamaephyte

#### Adansonia digitata

L.

E580759E-B909-576E-AFAF-FA0408BD2BD9

##### Distribution

Pluriregional African

##### Notes

Life Form: phanerophyte; Voucher: Nacoulma (APPG-69709)

#### Bombax costatum

Pellegr. & Vuillet

C4048598-112E-5E0E-9ACF-C38FEFBD3EBC

##### Distribution

Sudanian

##### Notes

Life Form: phanerophyte; Voucher: Nacoulma (APPG-69773)

#### Cienfuegosia heteroclada

Sprague

DA9775D1-4979-5580-B764-75F3D83B399B

##### Distribution

Sudanian

##### Notes

Life Form: chamaephyte; Voucher: Nacoulma 83 (OUA-13447)

#### Cola laurifolia

Mast.

36F6C891-52FD-5944-8D80-B79091ABADD4

##### Distribution

Guineo-Congolian-Sudano-Zambesian

##### Notes

Life Form: phanerophyte; Voucher: Nacoulma 132 (OUA-13496)

#### Corchorus fascicularis

Lam.

5F530D5B-E0D9-58E4-9EB6-BA6C70280C62

##### Distribution

Paleotropical

##### Notes

Life Form: therophyte; Voucher: Nacoulma 196 (OUA-13559)

#### Corchorus olitorius

L.

9DDCBF3A-F06B-51B9-BC7A-CC317A2437BE

##### Distribution

Paleotropical

##### Notes

Life Form: therophyte

#### Corchorus tridens

L.

6DB03B5A-3639-5286-B025-F5F8C05ABB51

##### Distribution

Paleotropical

##### Notes

Life Form: therophyte; Voucher: Nacoulma 40 (OUA-13405)

#### Dombeya quinqueseta

(Delile) Exell

14BF3DC6-BE04-56C0-B60B-E08F7F1C3A44

##### Distribution

Afrotropical

##### Notes

Life Form: phanerophyte; Voucher: Nacoulma (APPG-6818)

#### Grewia barteri

Burret

5247A7C4-AC43-5BA6-AE6C-80CBB53865FE

##### Distribution

Sudano-Zambesian

##### Notes

Life Form: phanerophyte; Voucher: Nacoulma (APPG-70052)

#### Grewia bicolor

Juss.

F2E10855-0711-516E-8466-2481DFFBE6D0

##### Distribution

Sudano-Zambesian

##### Notes

Life Form: phanerophyte; Voucher: Schumann (APPG-1370)

#### Grewia cissoides

Hutch. & Dalziel

E18441B6-D6B4-51CB-B17C-CADA496574B2

##### Distribution

Sudanian

##### Notes

Life Form: chamaephyte; Voucher: Zwarg 122 (FR)

#### Grewia flavescens

Juss.

39166718-09B9-5186-9C52-D331230E9D26

##### Distribution

Paleotropical

##### Notes

Life Form: phanerophyte; Voucher: Nacoulma 179 (OUA-13542)

#### Grewia lasiodiscus

K.Schum.

CBB341C0-8630-584A-82E0-2FAF0D402F65

##### Distribution

Sudanian

##### Notes

Life Form: phanerophyte; Voucher: Zwarg 109 (FR)

#### Grewia mollis

Juss.

77AFC2F1-53DE-535F-8F01-E58A9516C167

##### Distribution

Pluriregional African

##### Notes

Life Form: phanerophyte; Voucher: Nacoulma 200 (OUA-13563)

#### Grewia villosa

Willd.

4D2377BE-D9C5-57BE-92F1-A52286DAE214

##### Distribution

Paleotropical

##### Notes

Life Form: phanerophyte; Voucher: Nacoulma (APPG-70059)

#### Hibiscus cannabinus

L.

18FA9F6C-DF27-5D66-8853-42027DDF243B

Hibiscus asper Hook.f.

##### Distribution

Cultivated

##### Notes

Life Form: therophyte; Voucher: Schmidt et al. (FR-0007392)

#### Hibiscus congestiflorus

Hochr.

D2E567C3-D4C3-5D57-BB68-EEE6AAA301E7

##### Distribution

Sudanian

##### Notes

Life Form: therophyte

#### Hibiscus physaloides

Guill. & Perr.

3B83959D-2271-53EF-ACD9-30C53E58961E

##### Distribution

Sudanian

##### Notes

Life Form: phanerophyte; Voucher: Schmidt et al. (FR-0007629)

#### Hibiscus squamosus

Hochr.

E62CA1BE-E44D-573E-8225-515FDB695623

##### Distribution

Pantropical

##### Notes

Life Form: therophyte; Voucher: Nacoulma 34 (OUA-13399)

#### Kosteletzkya buettneri

Gürke

CCAA6498-F115-5C3C-92A9-E8B05E8D770F

##### Distribution

Sudano-Zambesian

##### Notes

Life Form: phanerophyte; Voucher: Nacoulma (OUA-17144)

#### Melochia corchorifolia

L.

D846C385-66EB-5D5D-AF65-E9A8A80AFBD2

##### Distribution

Paleotropical

##### Notes

Life Form: chamaephyte

#### Sida acuta

Burm.f.

4FEBB6EC-6603-5536-9DF8-BA0A60556EC3

##### Distribution

Pantropical

##### Notes

Life Form: chamaephyte; Voucher: Katharina Schumann (APPG-3885)

#### Sida alba

Cav.

9FE04EB7-CC5E-50A8-9C5D-6BDFA7188ACD

##### Distribution

Pantropical

##### Notes

Life Form: therophyte; Voucher: Zwarg 48 (FR)

#### Sida linifolia

Juss. ex Cav.

8CF5F9B6-51A4-56A6-BB72-B144F041C7DC

##### Distribution

Afro-American

##### Notes

Life Form: therophyte; Voucher: Schumann (FR-0083202)

#### Sida rhombifolia

L.

3F3667BF-4AE9-56BE-9A33-381A4FA12E13

##### Distribution

Pantropical

##### Notes

Life Form: chamaephyte; Voucher: Nacoulma 82 (OUA-13446)

#### Sida urens

L.

F2FF74EB-5C10-5944-A14B-96CD9735E7D2

##### Distribution

Pantropical

##### Notes

Life Form: therophyte; Voucher: Nacoulma 44 (OUA-13409)

#### Sterculia setigera

Delile

416E85E7-D561-5DD3-ACF5-DF0360C33D2C

##### Distribution

Afrotropical

##### Notes

Life Form: phanerophyte; Voucher: Schmidt et al. (FR-0007476)

#### Triumfetta rhomboidea

Jacq.

6CE2F615-D6E0-5A9C-9F0E-37809CB2CE8F

##### Distribution

Pantropical

##### Notes

Life Form: chamaephyte; Voucher: Zwarg 20 (FR)

#### Waltheria indica

L.

E2403B7A-627A-594D-B284-9B74ACB9F30F

##### Distribution

Pantropical

##### Notes

Life Form: chamaephyte; Voucher: Zwarg 19 (FR)

#### Wissadula rostrata

(Schumach. & Thonn.) Planch.

27B35593-084A-5B2C-8CD7-FCCE524EF186

Wissadula amplissima (L.) R.E.Fr.

##### Distribution

Afro-American

##### Notes

Life Form: phanerophyte; Voucher: Zwarg 22 (FR)

#### Marantaceae

30F2298F-C82F-5DFE-AC4F-5ED46DF561FA

#### Thalia geniculata

L.

ACA93D69-3052-5F2E-A696-49791FDA6DA0

##### Distribution

Pluriregional African

##### Notes

Life Form: geophyte; Voucher: Schmidt et al. (FR-0007382)

#### Marsileaceae

AD59547E-67C8-59F1-83BE-7D7A9AD39CF5

#### Marsilea minuta

L.

DDE15360-2BA8-57C5-AC61-4750BAD8F54D

##### Distribution

Afro-Malagasy

##### Notes

Life Form: hemicryptophyte

#### Marsilea polycarpa

Hook. & Grev.

96EC27A3-57CC-5063-A4A1-6EA4D13145B9

##### Distribution

Pantropical

##### Notes

Life Form: hemicryptophyte; Voucher: Nacoulma 4510 (OUA-17091)

#### Martyniaceae

0E75718D-C4EE-5780-91AB-09FA50FD3491

#### Martynia annua

L.

CF400AD0-5E51-513C-91EB-F1E02DAD5338

##### Distribution

Pluriregional African (introduced)

##### Notes

Life Form: therophyte; Voucher: Zizka (APPG-3856)

#### Meliaceae

B85DEF51-D0EA-57F7-83AA-D5AB5CAFFD84

#### Azadirachta indica

A.Juss.

6F61A636-AD69-5D55-B40E-B33337BE55D7

##### Distribution

Paleotropical (introduced)

##### Notes

Life Form: phanerophyte

#### Khaya senegalensis

(Desv.) A.Juss.

ED9E20B2-C33F-5465-9BDE-AE91AF29C65E

##### Distribution

Sudanian

##### Notes

Life Form: phanerophyte; Voucher: Zwarg 104 (FR)

#### Pseudocedrela kotschyi

(Schweinf.) Harms

072E9D81-D910-5A11-B20A-4994789AE348

##### Distribution

Sudanian

##### Notes

Life Form: phanerophyte

#### Trichilia emetica

Vahl

F67F61C2-35BC-55FA-9A7E-7FE94DD61977

##### Distribution

Pantropical

##### Notes

Life Form: phanerophyte

#### Molluginaceae

11A9E87D-E3BB-5F7D-B93D-BBD70EF9171A

#### Glinus lotoides

L.

4CF56363-5A18-5805-9020-42E355A7B212

##### Distribution

Pantropical

##### Notes

Life Form: hemicryptophyte; Voucher: Nacoulma 176 (OUA-13540)

#### Mollugo nudicaulis

Lam.

041AB7D7-6279-5C9F-8228-07329FFD4B32

##### Distribution

Paleotropical

##### Notes

Life Form: therophyte

#### Moraceae

96394AEE-B435-5066-98C3-FA72739D7ED2

#### Ficus abutilifolia

(Miq.) Miq.

2E218941-369B-5E4D-A824-E544B5296198

##### Distribution

Sudanian

##### Notes

Life Form: phanerophyte; Voucher: Schmidt et al. (FR-0007478)

#### Ficus asperifolia

Miq.

E57E3F42-5057-584E-8BA7-43DF17D627E4

##### Distribution

Afrotropical

##### Notes

Life Form: phanerophyte

#### Ficus glumosa

Delile

DADB91CA-2CB6-5CEB-A9B2-CDB87A6A56D6

##### Distribution

Sudanian

##### Notes

Life Form: phanerophyte; Voucher: Zwarg 125 (FR)

#### Ficus ingens

(Miq.) Miq.

A8274AE3-B61A-5948-9A00-000FEC6884F6

##### Distribution

Pluriregional African

##### Notes

Life Form: phanerophyte; Voucher: Nacoulma 161 (OUA-13525)

#### Ficus platyphylla

Delile

DF5F0FC7-1AB3-5E6B-8F16-18831AF9FC0C

##### Distribution

Sudano-Zambesian

##### Notes

Life Form: phanerophyte; Voucher: Nacoulma (APPG-69996)

#### Ficus sur

Forssk.

1B9682BC-4BAF-5F6E-9835-535CC92A2EE9

##### Distribution

Afrotropical

##### Notes

Life Form: phanerophyte

#### Ficus sycomorus

L.

3CD88506-C6B3-5A33-ABCD-35DEB6CE011E

##### Distribution

Afrotropical

##### Notes

Life Form: phanerophyte

#### Myrtaceae

5A453E47-31C4-51DE-A19E-C233ABFA80CF

#### Syzygium guineense

(Willd.) DC.

4D7FB4FF-0891-5056-8857-96684BC4B952

##### Distribution

Afrotropical

##### Notes

Life Form: phanerophyte; Vouchernomenc: Nacoulma 152 (OUA-13516)

#### Nyctaginaceae

1FEE88A7-DB58-576D-B7AB-2FA00EFCD547

#### Boerhavia erecta

L.

32A7BE73-7E7B-5793-BBDB-FFC350BEB933

##### Distribution

Cosmopolitan

##### Notes

Life Form: therophyte; Voucher: Nacoulma 174 (OUA-13538)

#### Nymphaeaceae

41060ADD-75F5-5CD7-8DAE-4A866310AE5E

#### Nymphaea lotus

L.

43FC7EFD-7491-5666-B026-9EB171D19785

##### Distribution

Paleotropical

##### Notes

Life Form: geophyte; Voucher: Nacoulma (APPG-70246)

#### Nymphaea maculata

Schumach. & Thonn.

53B20622-70B4-5ECB-9D96-86ACF542722B

##### Distribution

Afro-American

##### Notes

Life Form: geophyte; Voucher: Nacoulma (APPG-70253)

#### Nymphaea micrantha

Guill. & Perr.

072FED20-440D-500C-942C-BCC69B61AF0A

##### Distribution

Guineo-Congolian-Sudano-Zambesian

##### Notes

Life Form: geophyte; Voucher: Nacoulma 205 (OUA-13568)

#### Ochnaceae

422D3184-0A94-5EB5-AC29-D8DC80B1006B

#### Ochna schweinfurthiana

F.Hoffm.

E8DE054F-0BEC-5C8B-842F-6F1B34640D19

##### Distribution

Sudano-Zambesian

##### Notes

Life Form: phanerophyte; Voucher: Nacoulma 15 (OUA-13386)

#### Oleaceae

B33D6E9A-6B86-5065-9600-3EA2E870C8DD

#### Jasminum obtusifolium

Baker

45919764-E941-5A88-B9CB-54CC255FE929

##### Distribution

Sudanian

##### Notes

Life Form: phanerophyte

#### Onagraceae

46527464-C02E-5E7A-9420-4AAD83C6B374

#### Ludwigia erecta

(L.) H.Hara

B4DCD062-BFAD-5C44-BF64-30DEFB19B03D

##### Distribution

Afro-Malagasy (introduced)

##### Notes

Life Form: therophyte; Voucher: Schumann (FR-0083198)

#### Ludwigia hyssopifolia

(G.Don) Exell

FFB62741-1C60-5861-898C-6F5F0427BABB

##### Distribution

Sudanian

##### Notes

Life Form: therophyte; Voucher: Schumann (FR-0083197)

#### Ludwigia octovalvis

(Jacq.) P.H.Raven

38FE10CF-269F-560B-80BC-82D77309B4A3

##### Distribution

Pluriregional African

##### Notes

Life Form: therophyte; Voucher: Schumann (FR-0083245)

#### Ophioglossaceae

3240366D-8D49-5160-87A8-6DC4A63E3C2A

#### Ophioglossum costatum

R. Br.

D370BFB6-22BD-53AC-AA3B-DF0BFE3A64B6

##### Distribution

Sudano-Zambesian

##### Notes

Life Form: geophyte; Voucher: Nacoulma 246 (OUA-17234)

#### Ophioglossum reticulatum

L.

1E484C90-907E-53D0-A03D-8D0481505C19

##### Distribution

Pantropical

##### Notes

Life Form: geophyte; Voucher: Zizka (APPG-3435)

#### Ophioglossum thomasii

R.T. Clausen

53F95ACA-71CF-5C96-983D-CB97AD66A4FD

##### Distribution

Sudano-Zambesian

##### Notes

Life Form: geophyte; Voucher: Nacoulma 230 (OUA-17238); New species record for Burkina Faso.

#### Opiliaceae

0E65024A-0406-505A-A2E1-DA028B47EC2C

#### Opilia amentacea

Roxb.

1CF85AFE-3380-5B01-9C78-4D87842DA308

##### Distribution

Paleotropical

##### Notes

Life Form: phanerophyte; Voucher: Nacoulma (APPG-70274)

#### Orchidaceae

BD9DF39A-780C-52AB-A374-229AEB6C3D44

#### Eulophia cucullata

(Afzel. ex Sw.) Steud.

73F33472-648D-5F19-BB90-D5235BC25517

##### Distribution

Sudanian

##### Notes

Life Form: geophyte; Voucher: Nacoulma (APPG-69956)

#### Eulophia guineensis

Lindl.

6BD48CB7-E2C8-5E4E-8CA0-7E10B82F7733

##### Distribution

Guineo-Congolian

##### Notes

Life Form: geophyte; Voucher: Nacoulma (APPG-69975)

#### Habenaria longirostris

Summerh.

6E131517-3FEE-579D-B91B-A48C05D2E72C

##### Distribution

Sudanian

##### Notes

Life Form: geophyte; Voucher: Schmidt (APPG-3361)

#### Nervilia bicarinata

(Blume) Schltr.

67B3C145-7D84-5F34-BEA7-754A54479599

##### Distribution

Afro-Malagasy

##### Notes

Life Form: geophyte; Voucher: Nacoulma (APPG-70240)

#### Orobanchaceae

0D81BEEA-EB43-5265-B21F-FFFAC0464FBA

#### Buchnera hispida

Buch.-Ham. ex D.Don

424CA666-93A0-5685-9035-3E7723CAA0B6

##### Distribution

Sudanian

##### Notes

Life Form: therophyte; Voucher: Zwarg 110 (FR)

#### Micrargeria filiformis

(Schumach. & Thonn.) Hutch. & Dalziel

E88C9F4F-802C-51F7-A603-4E8B0A7BF3D9

##### Distribution

Sudanian

##### Notes

Life Form: therophyte

#### Rhamphicarpa fistulosa

(Hochst.) Benth.

52047813-9B17-5B01-9AA4-46C88439C316

##### Distribution

Pluriregional African

##### Notes

Life Form: therophyte; Voucher: Schumann (FR-0083126)

#### Sopubia ramosa

(Hochst.) Hochst.

ECE49042-4CBE-51D9-8270-A7377D283691

##### Distribution

Afrotropical

##### Notes

Life Form: phanerophyte

#### Striga asiatica

(L.) Kuntze

B92350DC-5EE1-5271-AEF0-3441915D418C

##### Distribution

Paleotropical

##### Notes

Life Form: therophyte; Voucher: Zwarg 3 (FR)

#### Striga aspera

Benth.

270E22E8-4192-57F5-9470-E789F5AE3F28

##### Distribution

Sudano-Zambesian

##### Notes

Life Form: chamaephyte; Voucher: Zwarg 6 (FR)

#### Striga baumannii

Engl.

045F0B0B-AD52-52C7-85E7-9F56D7BD00DD

##### Distribution

Afrotropical

##### Notes

Life Form: hemicryptophyte; Voucher: Nacoulma 4544 (OUA-17150)

#### Striga brachycalyx

Skan

9430AA20-8FBE-5B32-A004-249818DBE9E0

##### Distribution

Sudano-Zambesian

##### Notes

Life Form: chamaephyte; Voucher: Schumann (FR-0083125)

#### Striga forbesii

Benth.

0B3E03D8-477E-551F-BCCF-8A4F7918C5C0

##### Distribution

Afrotropical

##### Notes

Life Form: therophyte

#### Striga gesnerioides

(Willd.) Vatke

6F7000C4-E837-5D0A-A3E0-2107EDC5A84C

##### Distribution

Paleotropical

##### Notes

Life Form: chamaephyte

#### Striga hermonthica

(Delile) Benth.

B0CCEAA1-EA0D-5513-98C1-D0483E189D72

##### Distribution

Afro-Malagasy

##### Notes

Life Form: chamaephyte; Voucher: Nacoulma 4576 (OUA-17163)

#### Striga klingii

(Engl.) Skan

05865E2C-13EB-5292-8FDB-54C6349F4526

##### Distribution

Paleotropical

##### Notes

Life Form: therophyte; Voucher: Nacoulma 4531 (OUA-17113)

#### Striga macrantha

(Benth.) Benth.

DE4BD938-660B-5BA6-824C-71969A8EEF9C

##### Distribution

Pluriregional African

##### Notes

Life Form: therophyte; Voucher: Nacoulma 4483 (OUA-17061)

#### Striga passargei

Engl.

E9128A02-84DC-5C87-89CA-CE7339195500

##### Distribution

Sudano-Zambesian

##### Notes

Life Form: therophyte; Voucher: Schumann (FR-0083124)

#### Oxalidaceae

14AFBD6A-557A-54E9-B727-F9BEE5FCFFAD

#### Biophytum umbraculum

Welw.

48F12BC6-6C40-51D8-8D69-97546CA2CB4E

##### Distribution

Paleotropical

##### Notes

Life Form: therophyte; Voucher: Nacoulma 4606 (OUA-17170)

#### Passifloraceae

D83AD2F2-65DA-59C2-9B51-33AB06316500

#### Passiflora foetida

L.

3491F082-0798-5C2A-9EE0-7C4BFF90A5B6

##### Distribution

Pantropical (introduced)

##### Notes

Life Form: phanerophyte; Voucher: Katharina Schumann (APPG-2045)

#### Tricliceras pilosum

(Willd.) R.Fern.

DA6EA287-8B8E-5AD0-83AA-6FF23141A45A

##### Distribution

Sudanian

##### Notes

Life Form: therophyte; Voucher: Katharina Schumann (APPG-5700)

#### Pedaliaceae

D08CCA1B-140A-59A0-A677-18B7DA20DF85

#### Ceratotheca sesamoides

Endl.

E7D53BB8-CCDA-5AD6-9B00-9BE7511BB13A

##### Distribution

Sudano-Zambesian

##### Notes

Life Form: chamaephyte; Voucher: Nacoulma (APPG-69793)

#### Phyllanthaceae

883DE70B-07E9-5585-A962-9A3B92CB186E

#### Antidesma venosum

E.Mey. ex Tul.

DFEAE057-D936-52AA-8B03-551CBB2C6AAE

##### Distribution

Afrotropical

##### Notes

Life Form: phanerophyte; Voucher: Schmidt et al. (FR-0007657)

#### Bridelia ferruginea

Benth.

ECC2B736-465F-5ED7-A2A3-AF2034DFEF75

##### Distribution

Sudano-Zambesian

##### Notes

Life Form: phanerophyte

#### Bridelia scleroneura

Müll.Arg.

8AC74BD7-AC80-5553-8A7F-D4CD8A7D4C58

##### Distribution

Afro-Malagasy

##### Notes

Life Form: phanerophyte; Voucher: Zwarg 123 (FR)

#### Flueggea virosa

(Roxb. ex Willd.) Royle

3B2B5A14-739E-5837-B9D0-4A89F4696346

##### Distribution

Paleotropical

##### Notes

Life Form: phanerophyte; Voucher: Zwarg 86 (FR)

#### Hymenocardia acida

Tul.

9CFC76B3-6EDB-5949-9D58-9426A71396D4

##### Distribution

Afrotropical

##### Notes

Life Form: phanerophyte; Voucher: Schmidt et al. (FR-0007427)

#### Margaritaria discoidea

(Baill.) G.L.Webster

63013E13-946F-58BF-99D3-7EB2582E193F

##### Distribution

Pluriregional African

##### Notes

Life Form: phanerophyte; Voucher: Nacoulma 92 (OUA-13456)

#### Phyllanthus amarus

Schumach. & Thonn.

B6DCB07E-F421-5E64-A2F0-B0A67BB8F83B

##### Distribution

Pantropical

##### Notes

Life Form: therophyte; Voucher: Zwarg 53 (FR)

#### Piperaceae

01ACFB89-60A9-5739-AC3B-33501C4424A4

#### Peperomia pellucida

(L.) Kunth

0F1D6897-806C-5EFA-8735-5838E761E292

##### Distribution

Pantropical

##### Notes

Life Form: therophyte; Voucher: Nacoulma (APPG-70317)

#### Plantaginaceae

BA926882-304A-58C0-A7C6-8FBBCD854FD9

#### Bacopa crenata

(P.Beauv.) Hepper

E9F3D837-A8D0-51CC-809A-32D4EB68C5A8

##### Distribution

Afro-Malagasy

##### Notes

Life Form: therophyte; Voucher: Nacoulma 4621 (OUA-17203)

#### Bacopa floribunda

(R.Br.) Wettst.

A4FB89D7-C97C-5259-8663-B6FA3DD4C9A8

##### Distribution

Paleotropical

##### Notes

Life Form: therophyte; Voucher: Nacoulma 258 (OUA-17217)

#### Bacopa hamiltoniana

(Benth.) Wettst.

54F06761-4543-520D-9DB5-E56A8E69427F

##### Distribution

Paleotropical

##### Notes

Life Form: therophyte; Voucher: Küppers 464 (FR-0035808)

#### Dopatrium longidens

Skan

164E3F2E-C7BA-50D6-9D6B-AAB1D199EAF6

##### Distribution

Sudano-Zambesian

##### Notes

Life Form: therophyte; Voucher: Nacoulma 58 (OUA-13423)

#### Dopatrium senegalense

Benth.

585BF828-BEE7-5388-B3C3-BD288A338B57

##### Distribution

Sudano-Zambesian

##### Notes

Life Form: therophyte; Voucher: Nacoulma (APPG-69943)

#### Scoparia dulcis

L.

FB168668-3F34-530D-BE63-587D032CE892

##### Distribution

Pluriregional African (introduced)

##### Notes

Life Form: therophyte

#### Plumbaginaceae

6BC1C47E-7151-520E-93B1-A00C78155F83

#### Plumbago zeylanica

L.

4FB71E5E-7156-51EF-A783-1FA6FBF2D044

##### Distribution

Paleotropical (introduced)

##### Notes

Life Form: phanerophyte; Voucher: Schumann (FR-0083193)

#### Poaceae

9D6626A0-A384-51A7-A4A4-332D3A1B2F2D

#### Acroceras amplectens

Stapf

0F5856C1-AEC9-5E5E-B467-3497A5402A2F

##### Distribution

Pluriregional African

##### Notes

Life Form: chamaephyte

#### Acroceras zizanioides

(Kunth) Dandy

70BCEE9C-9A2B-5E4E-9FA9-BFD2E43D1984

##### Distribution

Pantropical

##### Notes

Life Form: chamaephyte

#### Anadelphia afzeliana

(Rendle) Stapf

78F6BB5A-356A-56AE-AC7C-44EF69ED6474

##### Distribution

Sudano-Zambesian

##### Notes

Life Form: hemicryptophyte; Voucher: Nacoulma 4548 (OUA-17146)

#### Andropogon chinensis

(Nees) Merr.

55D717B2-EC18-5AEC-AA6B-7ADF845D3ACD

Andropogon ascinodis C.B. Clarke

##### Distribution

Paleotropical

##### Notes

Life Form: hemicryptophyte; Voucher: Nacoulma 4516 (OUA-17085)

#### Andropogon fastigiatus

Sw.

83A6A11D-86BD-5724-8A22-BE0E53B0EE16

##### Distribution

Pantropical

##### Notes

Life Form: therophyte; Voucher: Zwarg 46 (FR)

#### Andropogon gayanus

Kunth

32A53849-06EA-5D3C-BDD0-9E227BE871D1

##### Distribution

Sudanian

##### Notes

Life Form: hemicryptophyte; Voucher: Nacoulma 4610 (OUA-17166)

#### Andropogon pseudapricus

Stapf

B54E0236-3F2A-5AC8-85F2-E67BF168E96D

##### Distribution

Afro-American

##### Notes

Life Form: therophyte; Voucher: Nacoulma 4537 (OUA-17108)

#### Andropogon schirensis

Hochst.

94C31621-FD6E-57D1-8A6C-F582114B0833

##### Distribution

Pluriregional African

##### Notes

Life Form: hemicryptophyte; Voucher: Nacoulma 182 (OUA-13545)

#### Andropogon tectorum

Schumach. & Thonn.

40FCE9FE-B289-553F-8A02-6F7F2F8DE821

##### Distribution

Sudanian

##### Notes

Life Form: hemicryptophyte; Voucher: Nacoulma 4501 (OUA-17100)

#### Aristida adscensionis

L.

2BA6962F-12BE-5D33-9BAE-89C8A9635C8B

##### Distribution

Pantropical

##### Notes

Life Form: therophyte

#### Aristida hordeacea

Kunth

665FD8AD-FB14-58BA-8189-6014542CA7B5

##### Distribution

Sudano-Zambesian

##### Notes

Life Form: therophyte

#### Aristida kerstingii

Pilg.

5D70ABF3-2E52-55BA-B853-02B51DBF1DEF

##### Distribution

Sudanian

##### Notes

Life Form: therophyte; Voucher: Nacoulma 4581 (OUA-17158)

#### Brachiaria deflexa

(Schumach.) C.E.Hubb. ex Robyns

BBB793D1-BF50-5A15-88C5-341B02D3C8CE

##### Distribution

Paleotropical

##### Notes

Life Form: therophyte

#### Brachiaria falcifera

(Trin. ex Nees) Stapf

9DE795B9-458F-5469-AD46-D61032349167

##### Distribution

Sudanian

##### Notes

Life Form: hemicryptophyte; Voucher: Schumann (FR-0083189)

#### Brachiaria lata

(Schumach.) C.E.Hubb.

2D229647-75E4-57A9-82C6-AC4554CA7D8F

##### Distribution

Paleotropical

##### Notes

Life Form: therophyte

#### Brachiaria serrata

(Thunb.) Stapf

F9859DCD-5374-5E7A-BD98-133461804111

##### Distribution

Afrotropical

##### Notes

Life Form: hemicryptophyte; Voucher: Nacoulma (OUA-17186)

#### Brachiaria stigmatisata

(Mez) Stapf

D6D6AE00-FF64-5F7B-85D7-79C37BFA3F42

##### Distribution

Sudanian

##### Notes

Life Form: therophyte; Voucher: Schumann (FR-0083187)

#### Brachiaria villosa

(Lam.) A.Camus

689FD1A6-5C21-5887-9E97-FC22BC0A25D4

##### Distribution

Paleotropical

##### Notes

Life Form: therophyte; Voucher: Zwarg 27 (FR)

#### Chasmopodium caudatum

(Hack.) Stapf

9963E38B-F65C-555E-B3F9-07BBBDA21250

##### Distribution

Sudano-Zambesian

##### Notes

Life Form: therophyte

#### Chloris gayana

Kunth

F1429967-45E2-59AC-8576-5093532F7F27

##### Distribution

Sudano-Zambesian

##### Notes

Life Form: therophyte; Voucher: Nacoulma (OUA-17319); New species record for Burkina Faso

#### Chloris pilosa

Schumach. & Thonn.

0C8A272A-485C-5E0A-804C-987E49D8536D

##### Distribution

Sudano-Zambesian

##### Notes

Life Form: therophyte

#### Chrysopogon nigritanus

(Benth.) Veldkamp

358F49EF-7B67-5921-B6B0-255578713334

##### Distribution

Sudano-Zambesian

##### Notes

Life Form: hemicryptophyte

#### Ctenium elegans

Kunth

7BFB808E-F480-585F-A148-5CDE831FFA76

##### Distribution

Sudanian

##### Notes

Life Form: therophyte; Voucher: Zwarg 1 (FR)

#### Ctenium newtonii

Hack.

1BD2F258-CBD5-5F07-8023-1CCB9B236B8A

##### Distribution

Afrotropical

##### Notes

Life Form: hemicryptophyte

#### Ctenium villosum

Berhaut

B3B0E56B-EFF1-5430-8BA5-1652B6113CD7

##### Distribution

Sudanian

##### Notes

Life Form: therophyte; Voucher: Küppers 458 (FR-0015111)

#### Cymbopogon caesius

(Nees ex Hook. & Arn.) Stapf

1CB2BA35-AA8D-550A-BF53-CD5A5DA5F7CC

##### Distribution

Afrotropical

##### Notes

Life Form: hemicryptophyte; Voucher: Schmidt et al. (FR-0007430)

#### Cymbopogon schoenanthus

(L.) Spreng.

72D4D4D2-D12E-54F6-87BF-AE1C76EFCD93

##### Distribution

Pantropical

##### Notes

Life Form: hemicryptophyte; Voucher: Nacoulma 4599 (OUA-17177)

#### Cynodon dactylon

(L.) Pers.

BF1B1287-FD72-5DB1-881D-9352BA908F79

##### Distribution

Sudano-Zambesian

##### Notes

Life Form: hemicryptophyte

#### Digitaria gayana

(Kunth) A.Chev.

0B1218D8-AF94-5594-AAC2-1742F2DF8D6A

##### Distribution

Pantropical

##### Notes

Life Form: therophyte; Voucher: Zwarg 44 (FR)

#### Digitaria horizontalis

Willd.

8A2590F1-FC13-5052-9981-0C670FF1F688

##### Distribution

Afrotropical

##### Notes

Life Form: therophyte

#### Diheteropogon amplectens

(Nees) Clayton

FE1A20AF-0234-5A31-BC44-F1EE29BB0C2C

##### Distribution

Pantropical

##### Notes

Life Form: hemicryptophyte

#### Echinochloa colona

(L.) Link

804AB61D-EF8B-5CE1-9445-FD81F98913DA

##### Distribution

Pantropical

##### Notes

Life Form: therophyte

#### Echinochloa pyramidalis

(Lam.) Hitchc. & Chase

79D58446-BA55-53E5-8DAF-D2177E9B3ACE

##### Distribution

Afro-American

##### Notes

Life Form: hemicryptophyte; Voucher: Schmidt et al. (FR-0007578)

#### Echinochloa stagnina

(Retz.) P.Beauv.

1F748163-B546-55B2-AA29-4CD2298DBA2C

##### Distribution

Paleotropical

##### Notes

Life Form: therophyte; Voucher: Schmidt et al. (FR-0007406)

#### Eleusine indica

(L.) Gaertn.

157F3784-3508-5634-8C16-1D32CA580219

##### Distribution

Pantropical

##### Notes

Life Form: therophyte; Voucher: Schumann (FR-0083175)

#### Elionurus ciliaris

Kunth

E80E5925-0343-5D2C-B5F4-EE67A72F48DD

Elionurus pobeguinii Stapf

##### Distribution

Sudanian

##### Notes

Life Form: hemicryptophyte; Voucher: Nacoulma (OUA-17123)

#### Elionurus elegans

Kunth

7D0E52A5-9B51-5CC8-A81C-666EF1CB3655

##### Distribution

Sudano-Zambesian

##### Notes

Life Form: therophyte

#### Elymandra androphila

(Stapf) Stapf

D593D472-5F3A-5402-B400-8679A9C83483

##### Distribution

Sudano-Zambesian

##### Notes

Life Form: hemicryptophyte

#### Elytrophorus spicatus

(Willd.) A.Camus

4AB4EF35-CFBA-54AF-A637-8D88A5AB3FA8

##### Distribution

Paleotropical

##### Notes

Life Form: therophyte; Voucher: Schmidt et al. (FR-0007405)

#### Eragrostis amabilis

(L.) Wight & Arn.

871C6227-F45D-56CE-A613-0CFC7B145ABF

Eragrostis tenella (L.) P.Beauv. ex Roem. & Schult.

##### Distribution

Paleotropical

##### Notes

Life Form: therophyte

#### Eragrostis ciliaris

(L.) R.Br.

089AFAE2-5D6B-513F-9C02-F6998D018EAC

##### Distribution

Paleotropical

##### Notes

Life Form: therophyte; Voucher: Schumann (FR-0083172)

#### Eragrostis pilosa

(L.) P.Beauv.

278B98B5-2A1B-5372-B242-91A580C773C4

##### Distribution

Cosmopolitan

##### Notes

Life Form: therophyte

#### Eragrostis tremula

Hochst. ex Steud.

D58A2CAB-3EFA-5928-AA76-E4E860E627D5

##### Distribution

Paleotropical

##### Notes

Life Form: therophyte; Voucher: Zwarg 111 (FR)

#### Eragrostis turgida

(Schumach.) De Wild.

13801C62-4B6A-59AB-9820-D6D26C68E213

##### Distribution

Paleotropical

##### Notes

Life Form: therophyte; Voucher: Zwarg 28 (FR)

#### Euclasta condylotricha

(Steud.) Stapf

D09F46D5-1499-50AF-BC77-C1FFA5C04919

##### Distribution

Pantropical

##### Notes

Life Form: therophyte

#### Hackelochloa granularis

(L.) Kuntze

7C39909E-A36D-59DF-9C52-684446035B00

##### Distribution

Pantropical

##### Notes

Life Form: therophyte; Voucher: Zwarg 33 (FR)

#### Heteropogon contortus

(L.) P.Beauv. ex Roem. & Schult.

AC49ECE5-C0F2-544B-AC20-7BA67585C775

##### Distribution

Pantropical

##### Notes

Life Form: hemicryptophyte; Voucher: Nacoulma (APPG-70065)

#### Hyparrhenia cyanescens

(Stapf) Stapf

A70A6FA3-BB34-522B-9C8D-3DD8858D2FE3

##### Distribution

Guineo-Congolian

##### Notes

Life Form: hemicryptophyte; Voucher: Nacoulma 202 (OUA-13565)

#### Hyparrhenia diplandra

(Hack.) Stapf

DE81217D-AC49-503D-A89A-72E8540FB031

##### Distribution

Pluriregional African

##### Notes

Life Form: hemicryptophyte

#### Hyparrhenia glabriuscula

(Hochst. ex A.Rich.) Andersson ex Stapf

FD6C4817-2B5C-5362-BF1F-5BF6ED7BA499

##### Distribution

Sudano-Zambesian

##### Notes

Life Form: hemicryptophyte; Voucher: Schumann (FR-0083165)

#### Hyparrhenia involucrata

Stapf

510359D0-B609-5D91-8F72-2B404598B518

##### Distribution

Sudanian

##### Notes

Life Form: therophyte; Voucher: Nacoulma 4547 (OUA-17147)

#### Hyparrhenia rufa

(Nees) Stapf

75448F2F-C52B-5691-A7FA-A560077B900F

##### Distribution

Pantropical

##### Notes

Life Form: hemicryptophyte; Voucher: Schmidt et al. 6111 (FR-0116607)

#### Hyparrhenia smithiana

(Hook.f.) Stapf

81508820-E32B-5B9B-91F3-9DF023F942F5

##### Distribution

Sudanian

##### Notes

Life Form: hemicryptophyte; Voucher: Nacoulma 186 (OUA-13549)

#### Hyparrhenia subplumosa

Stapf

A45A9662-3E96-581A-9965-4DE224C7A308

##### Distribution

Sudano-Zambesian

##### Notes

Life Form: hemicryptophyte; Voucher: Nacoulma 4532 (OUA-17112)

#### Hyperthelia dissoluta

(Nees ex Steud.) Clayton

3BBE32EF-6F86-5BCD-8A50-BAD04502FB9C

##### Distribution

Afro-American

##### Notes

Life Form: hemicryptophyte

#### Imperata cylindrica

(L.) Raeusch.

949AF8A5-CAC8-55A6-96C1-8F559C14CF8D

##### Distribution

Afro-Malagasy

##### Notes

Life Form: geophyte

#### Loudetia flavida

(Stapf) C.E.Hubb.

973DCF99-FFB2-55E5-86A4-A3B21E5605A3

##### Distribution

Afro-American

##### Notes

Life Form: therophyte; Voucher: Nacoulma 1 (OUA-13370)

#### Loudetia simplex

(Nees) C.E.Hubb.

2FEBB745-0745-5355-BDFA-5C12EC8E204C

##### Distribution

Sudano-Zambesian

##### Notes

Life Form: hemicryptophyte; Voucher: Nacoulma 4549 (OUA-17145)

#### Loudetia togoensis

(Pilg.) C.E.Hubb.

E2A6EE9C-93E3-53C1-A73A-D7468E926AEE

##### Distribution

Sudanian

##### Notes

Life Form: therophyte; Voucher: Nacoulma 4553 (OUA-17140)

#### Loudetiopsis kerstingii

(Pilg.) Conert

857BA742-AFB0-5F64-A363-DA4F2AC4F266

##### Distribution

Afrotropical

##### Notes

Life Form: hemicryptophyte; Voucher: Schumann (FR-0083162)

#### Loxodera ledermannii

(Pilg.) Launert

7CA526DF-2352-5CBF-B19F-2050A9B8F425

##### Distribution

Sudanian

##### Notes

Life Form: therophyte; Voucher: Nacoulma (APPG-70183); New species record for Burkina Faso.

#### Microchloa indica

(L.f.) P.Beauv.

0F581E49-C4D7-5BED-81E9-D90233847F9F

##### Distribution

Pantropical

##### Notes

Life Form: therophyte; Voucher: Zwarg 14 (FR)

#### Monocymbium ceresiiforme

(Nees) Stapf

27567AD0-C2E4-5327-B2CB-71AC4D8213BC

##### Distribution

Pluriregional African

##### Notes

Life Form: hemicryptophyte; Voucher: Schumann (FR-0083163)

#### Oropetium aristatum

(Stapf) Pilg.

D912AB3A-C828-5EBE-8AC5-77F556F2D224

##### Distribution

Sudano-Zambesian

##### Notes

Life Form: hemicryptophyte; Voucher: Schumann (FR-0083161)

#### Oryza longistaminata

A.Chev.& Roehr.

92A31480-1DB0-5EE1-B5BF-1AFF19F4AF62

##### Distribution

Afro-Malagasy

##### Notes

Life Form: hemicryptophyte; Voucher: Schumann (FR-0083160)

#### Oxytenanthera abyssinica

(A.Rich.) Munro

05100020-E8C7-5427-B779-33409494B949

##### Distribution

Sudano-Zambesian

##### Notes

Life Form: phanerophyte

#### Panicum anabaptistum

Steud.

7F7B5353-BCBB-5B27-A3EC-9985421C4837

##### Distribution

Sudanian

##### Notes

Life Form: hemicryptophyte; Voucher: Schmidt et al. (FR-0007417)

#### Panicum fluviicola

Steud.

A78C4B3B-7FA9-58FB-B172-1823ABC7CA3B

##### Distribution

Pluriregional African

##### Notes

Life Form: hemicryptophyte; Voucher: Nacoulma 216 (OUA-13579)

#### Panicum humile

Steud.

36308208-1344-5B6E-A177-535D73B6A632

Panicum walense Mez

##### Distribution

Sudano-Zambesian

##### Notes

Life Form: therophyte; Voucher: Schmidt et al. (FR-0007407)

#### Panicum maximum

Jacq.

0BB0C048-8E69-5C1A-BD43-C57BBAE1E96A

##### Distribution

Pantropical

##### Notes

Life Form: hemicryptophyte

#### Panicum pansum

Rendle

20B345B7-946D-5C3D-A228-152D7E4F4115

##### Distribution

Sudano-Zambesian

##### Notes

Life Form: therophyte; Voucher: Zwarg 25 (FR)

#### Panicum phragmitoides

Stapf

6BEE6985-394E-5AA5-A7DD-40A05DB1954F

##### Distribution

Sudanian

##### Notes

Life Form: hemicryptophyte

#### Paspalum scrobiculatum

L.

4AB32F99-55C3-59BE-A9E3-AD6F72F114B6

##### Distribution

Paleotropical

##### Notes

Life Form: hemicryptophyte; Voucher: Katharina Schumann (APPG-3868)

#### Pennisetum pedicellatum

Trin.

FE359EA5-F463-5887-AB4B-D5FA4808223C

##### Distribution

Paleotropical

##### Notes

Life Form: therophyte; Voucher: Zwarg 39 (FR)

#### Pennisetum polystachion

(L.) Schult.

948AF1BA-3DF7-54DF-905C-F823F379E3A3

##### Distribution

Pantropical

##### Notes

Life Form: therophyte; Voucher: Zwarg 51 (FR)

#### Pennisetum unisetum

(Nees) Benth.

85A4C903-4F51-5C57-A096-3D1C074FA0F9

Pennisetum unisetum Beckeropsis uniseta (Nees) K.Schum.

##### Distribution

Sudano-Zambesian

##### Notes

Life Form: hemicryptophyte

#### Pennisetum violaceum

(Lam.) Rich.

8A73DC6A-E9C9-5A81-AAA3-73F9C8CD48D9

##### Distribution

Sudanian

##### Notes

Life Form: therophyte; Voucher: Nacoulma (APPG-70314)

#### Rhytachne triaristata

(Steud.) Stapf

2237690B-C249-5E97-BAA3-F00A7458691D

##### Distribution

Paleotropical

##### Notes

Life Form: therophyte; Voucher: Schumann (FR-0083156)

#### Rottboellia cochinchinensis

(Lour.) Clayton

FDF8F043-CA0A-5CF5-9093-1F9E83F2D40B

##### Distribution

Pantropical

##### Notes

Life Form: therophyte

#### Sacciolepis africana

C.E.Hubb. & Snowden

809D7124-0BC8-56C9-87F1-05E6AEE6A622

##### Distribution

Paleotropical

##### Notes

Life Form: therophyte; Voucher: Schumann (FR-0083154)

#### Sacciolepis ciliocincta

(Pilg.) Stapf

3A91B717-4FE2-5F2B-BC45-B6944C261AC9

##### Distribution

Sudano-Zambesian

##### Notes

Life Form: therophyte

#### Sacciolepis micrococca

Mez

1ED43E36-711D-5699-9BCD-E711C52E1D0A

##### Distribution

Sudano-Zambesian

##### Notes

Life Form: therophyte; Voucher: Schumann (FR-0083153)

#### Schizachyrium brevifolium

(Sw.) Buse

A2C3FFC5-0E21-5AAF-AADB-80F5A0E8614C

##### Distribution

Pantropical

##### Notes

Life Form: therophyte; Voucher: Schmidt et al. (FR-0007400)

#### Schizachyrium exile

(Hochst.) Pilg.

EC43EFAE-D972-5F5A-A3E0-3187E4DBBB53

##### Distribution

Paleotropical

##### Notes

Life Form: therophyte; Voucher: Schumann (FR-0083150)

#### Schizachyrium nodulosum

(Hack.) Stapf

DC37A75E-81E3-5D9C-9136-514F7D6928D3

##### Distribution

Pluriregional African

##### Notes

Life Form: therophyte

#### Schizachyrium platyphyllum

(Franch.) Stapf

9254A0F3-B080-5E9E-9374-D45EAAF6236E

##### Distribution

Sudanian

##### Notes

Life Form: therophyte

#### Schizachyrium ruderale

Clayton

62846A67-C9F4-54EC-95AA-9F8C2FBED23B

##### Distribution

Sudanian

##### Notes

Life Form: therophyte; Voucher: Schumann (FR-0083149)

#### Schizachyrium rupestre

(K.Schum.) Stapf

47A619DF-EBF2-57B8-9CC8-5646615033DC

##### Distribution

Sudanian

##### Notes

Life Form: hemicryptophyte; Voucher: Schmidt et al. (FR-0007480)

#### Schizachyrium sanguineum

(Retz.) Alston

CC3D4824-354B-5FD4-A659-873DFA70A7C6

##### Distribution

Pantropical

##### Notes

Life Form: hemicryptophyte

#### Schizachyrium urceolatum

(Hack.) Stapf

B9BEB886-2F20-51DE-A343-B3E6DFC032DB

##### Distribution

Sudano-Zambesian

##### Notes

Life Form: therophyte; Voucher: Schumann (FR-0083148)

#### Schoenefeldia gracilis

Kunth

8647FE37-28F1-5B45-A454-D651FDC0E565

##### Distribution

Paleotropical

##### Notes

Life Form: therophyte; Voucher: Zwarg 112 (FR)

#### Setaria barbata

(Lam.) Kunth

E61FA7E0-29E9-566C-9167-31AF2E02857B

##### Distribution

Pantropical

##### Notes

Life Form: therophyte; Voucher: Katharina Schumann (APPG-4475)

#### Setaria pumila

(Poir.) Roem. & Schult.

DA313773-9749-530C-86E7-92E4A89E0512

##### Distribution

Afrotropical

##### Notes

Life Form: therophyte; Voucher: Zizka (APPG-3882)

#### Setaria sphacelata

(Schumach.) Stapf & C.E.Hubb. ex Moss

2AB1F05F-BD1B-565A-B268-4A95EB39C6F3

##### Distribution

Sudano-Zambesian

##### Notes

Life Form: therophyte

#### Sorghastrum bipennatum

(Hack.) Pilg.

C2CA2617-CE08-5DD3-A18C-C9C5067D3721

##### Distribution

Paleotropical

##### Notes

Life Form: therophyte; Voucher: Küppers 463 (FR-0015883)

#### Sporobolus festivus

Hochst. ex A.Rich.

D416BC2E-C42C-5FF7-961F-3B7A268D2DB0

##### Distribution

Pluriregional African

##### Notes

Life Form: hemicryptophyte; Voucher: Zwarg 36 (FR)

#### Sporobolus pyramidalis

P.Beauv.

F9D7D7C1-6B1D-5B99-9EE1-EC8E06948C2A

##### Distribution

Afro-American

##### Notes

Life Form: hemicryptophyte

#### Thelepogon elegans

Roth

E8A53822-D3E5-57BF-A67C-569EF72236EC

##### Distribution

Sudano-Zambesian

##### Notes

Life Form: therophyte; Voucher: Schmidt et al. (FR-0007637)

#### Trachypogon spicatus

(L.f.) Kuntze

A93C23BB-3DD6-530E-B140-5D1330BA8244

##### Distribution

Paleotropical

##### Notes

Life Form: therophyte

#### Tripogon minimus

(A.Rich.) Hochst. ex Steud.

AFA4982F-B93B-539D-9B80-2946B91AD550

##### Distribution

Pluriregional African

##### Notes

Life Form: hemicryptophyte; Voucher: Schumann (FR-0083142)

#### Urelytrum muricatum

C.E.Hubb.

F3299C68-0FB0-5E3D-AB41-0D381C813E6B

##### Distribution

Sudanian

##### Notes

Life Form: hemicryptophyte

#### Urochloa jubata

(Fig. & De Not.) Sosef

39FED583-FBFA-531E-AF1C-0D21C41EA42B

Brachiaria jubata (Fig. & De Not.) Stapf

##### Distribution

Afrotropical

##### Notes

Life Form: hemicryptophyte

#### Polygalaceae

965F23D7-17AA-5DE8-B481-BCCCF363853F

#### Polygala arenaria

Willd.

55DC9877-3D51-5008-BD66-FAA0B6EC44F2

##### Distribution

Pluriregional African

##### Notes

Life Form: therophyte; Voucher: Zwarg 9 (FR)

#### Polygala multiflora

Poir.

B33FE9BC-4C1F-5E1C-9A58-04F6B5B28BC4

##### Distribution

Sudanian

##### Notes

Life Form: therophyte; Voucher: Nacoulma 87 (OUA-13451)

#### Securidaca longipedunculata

Fresen.

86BD494A-BE1E-5991-A94A-3ED3A794CCC4

##### Distribution

Afrotropical

##### Notes

Life Form: phanerophyte; Voucher: Zwarg 124 (FR)

#### Polygonaceae

8B755D66-BC5C-52B5-B840-C9C38AFF9279

#### Persicaria decipiens

(R.Br.) K.L.Wilson

54BCA8BF-18E6-5D4F-80B0-CC63EEC6F8BA

##### Distribution

Paleotropical

##### Notes

Life Form: therophyte; Voucher: Schumann (FR-0083139)

#### Persicaria senegalensis

(Meisn.) Soják

CA342EFF-A09E-53E5-BC17-09FF915939DE

Polygonum senegalense Meisn.

##### Distribution

Pluriregional African

##### Notes

Life Form: chamaephyte; Voucher: Nacoulma 4560 (OUA-17153)

#### Pontederiaceae

02EE540E-8B2A-501A-815F-1910EF279155

#### Eichhornia natans

(P.Beauv.) Solms

306B9663-1E30-5D75-B3C9-AE1BB4EDEDD5

##### Distribution

Afro-American

##### Notes

Life Form: therophyte; Voucher: Nacoulma 4498 (OUA-17068)

#### Heteranthera callifolia

Rchb. ex Kunth

4F58F37D-0A86-586A-9762-39A8AC23FCA5

##### Distribution

Afro-American

##### Notes

Life Form: therophyte; Voucher: Nacoulma 4591 (OUA-17185)

#### Monochoria brevipetiolata

Verdc.

11D97DD8-49BD-5357-88A6-D860193FA0E6

##### Distribution

Sudano-Zambesian

##### Notes

Life Form: hemicryptophyte; Voucher: Nacoulma 4508 (OUA-17093)

#### Portulacaceae

1B605EAE-511E-586C-8945-2B9FCE4AC83B

#### Portulaca foliosa

Ker Gawl.

B0211734-B0EF-562B-A0D2-99F9F075FDF2

##### Distribution

Pantropical

##### Notes

Life Form: therophyte; Voucher: Zwarg 117 (FR)

#### Portulaca quadrifida

L.

1D5D03AC-24B9-5B96-8FB5-938833CB4B20

##### Distribution

Paleotropical

##### Notes

Life Form: therophyte; Voucher: Schmidt et al. (FR-0007669)

#### Primulaceae

59D2BCB1-6ED3-5FB1-BA16-7DC4021B1FE7

#### Anagallis pumila

Sw.

B08BA80B-E05A-5C28-902E-F247167B59DD

##### Distribution

Paleotropical

##### Notes

Life Form: therophyte; Voucher: Schmidt et al. (FR-0007697)

#### Pteridaceae

47776CD7-247A-5702-8573-E0F1C1DD44FC

#### Ceratopteris cornuta

(P. Beauv.) Lepr.

CC4A1D7E-CFC0-5930-AB15-ECB3B763A360

Ceratopteris thalictroides (L.) Brongn.

##### Distribution

Afrotropical

##### Notes

Life Form: hydrophyte

#### Rhamnaceae

7E43988B-6232-563E-9BD5-803BCCA8317C

#### Ziziphus abyssinica

Hochst. ex A.Rich.

84DCA324-101F-5E88-A008-1A2061E491BE

##### Distribution

Afrotropical

##### Notes

Life Form: phanerophyte; Voucher: Zwarg 82 (FR)

#### Ziziphus mauritiana

Lam.

79D6441A-2E67-5C6E-A7FA-A2D3A75A31A1

##### Distribution

Paleotropical

##### Notes

Life Form: phanerophyte

#### Ziziphus mucronata

Willd.

C02419DB-FE2F-5980-A531-222100A987A2

##### Distribution

Pluriregional African

##### Notes

Life Form: phanerophyte; Voucher: Nacoulma (APPG-70453)

#### Ziziphus spina-christi

(L.) Desf.

D3EF795D-9283-5DEF-B1C1-3AA786A0399B

##### Distribution

Paleotropical

##### Notes

Life Form: phanerophyte; Voucher: Nacoulma 121 (OUA-13485)

#### Rubiaceae

8B68277D-D30E-5925-BCF3-82EE16A4C920

#### Cremaspora triflora

(Thonn.) K.Schum.

7B979788-2361-5C65-8DFD-52655ED7F7E1

##### Distribution

Afro-Malagasy

##### Notes

Life Form: phanerophyte; Voucher: Nacoulma (OUA-17130)

#### Crossopteryx febrifuga

(Afzel. ex G.Don) Benth.

6A6B6FBD-09E9-5A9C-B33D-09AE069371D6

##### Distribution

Afrotropical

##### Notes

Life Form: phanerophyte; Voucher: Zwarg 88 (FR)

#### Fadogia agrestis

Schweinf. ex Hiern

E1D0348A-EF9D-5974-88D8-C5D65EA80BB9

##### Distribution

Sudanian

##### Notes

Life Form: chamaephyte; Voucher: Nacoulma 160 (OUA-13524)

#### Feretia apodanthera

Delile

05B4E30E-3D0C-5228-8699-047655A110C6

##### Distribution

Sudanian

##### Notes

Life Form: phanerophyte; Voucher: Zwarg 98 (FR)

#### Gardenia aqualla

Stapf & Hutch.

2FDF236E-1A47-51A0-8C7F-FEB7455B8A01

##### Distribution

Sudano-Zambesian

##### Notes

Life Form: phanerophyte; Voucher: Nacoulma 4519 (OUA-17082)

#### Gardenia erubescens

Stapf & Hutch.

06A42E2D-53DA-5AEA-AA90-8EC4680E9916

##### Distribution

Sudanian

##### Notes

Life Form: phanerophyte; Voucher: Nacoulma (APPG-70012)

#### Gardenia sokotensis

Hutch.

546EC613-AAE0-571C-B775-8BAE953251BF

##### Distribution

Sudanian

##### Notes

Life Form: phanerophyte; Voucher: Nacoulma (APPG-70020)

#### Gardenia ternifolia

Schumach. & Thonn.

72CC6E3A-5CF6-572F-980F-0FE112DE68EE

##### Distribution

Paleotropical

##### Notes

Life Form: phanerophyte; Voucher: Nacoulma 199 (OUA-13562)

#### Keetia cornelia

(Cham. & Schltdl.) Bridson

43C0A0EA-D331-5361-8161-25FDFF75D01B

Canthium cornelia Cham. & Schltdl.

##### Distribution

Guineo-Congolian

##### Notes

Life Form: phanerophyte; Voucher: Nacoulma 131 (OUA-13495)

#### Keetia venosa

(Oliv.) Bridson

D43104F9-C016-57B3-83F8-8ACC428A8897

##### Distribution

Pluriregional African

##### Notes

Life Form: phanerophyte; Voucher: Janßen (APPG-5627)

#### Kohautia confusa

(Hutch. & Dalziel) Bremek.

89CA5AB0-3980-5B1A-832A-9C5705C815C7

##### Distribution

Sudanian

##### Notes

Life Form: therophyte

#### Kohautia grandiflora

DC.

E18FD708-B6F3-5ED0-8778-274C404FE5DF

##### Distribution

Sudano-Zambesian

##### Notes

Life Form: therophyte; Voucher: Schumann (FR-0083134)

#### Kohautia tenuis

(Bowdich) Mabb.

E5FB19F4-4CFF-57B6-8582-F89570704A40

Kohautia senegalensis Cham. & Schltdl.

##### Distribution

Pluriregional African

##### Notes

Life Form: therophyte; Voucher: Zwarg 34 (FR)

#### Macrosphyra longistyla

(DC.) Hiern

8D4998E4-21ED-5FF1-9DB5-80D5BDD2333B

##### Distribution

Sudano-Zambesian

##### Notes

Life Form: phanerophyte; Voucher: Nacoulma 172 (OUA-13536)

#### Mitracarpus hirtus

(L.) DC.

0053E59A-353E-5488-AB55-DBA14A61946A

##### Distribution

Pantropical

##### Notes

Life Form: therophyte; Voucher: Zwarg 42 (FR)

#### Mitragyna inermis

(Willd.) Kuntze

91429963-853C-5E92-8B12-7169848DB4AA

##### Distribution

Sudanian

##### Notes

Life Form: phanerophyte; Voucher: Schmidt et al. (FR-0007387)

#### Morelia senegalensis

A.Rich. ex DC.

4BE72C20-79CB-5373-A044-8458D3BD2FD4

##### Distribution

Guineo-Congolian-Sudano-Zambesian

##### Notes

Life Form: phanerophyte; Voucher: Nacoulma 134 (OUA-13498)

#### Oldenlandia capensis

L.f.

BB10FE82-2FF6-55FE-AD30-C72B27034023

##### Distribution

Cosmopolitan

##### Notes

Life Form: therophyte

#### Pavetta crassipes

K.Schum.

9C5C3C78-F78B-5F38-9FC3-181D72FBB580

##### Distribution

Afrotropical

##### Notes

Life Form: phanerophyte; Voucher: Nacoulma 93 (OUA-13457)

#### Pouchetia africana

A.Rich. ex DC.

BA96226A-C790-5BC0-BEC2-9BB8C9344D97

##### Distribution

Afrotropical

##### Notes

Life Form: phanerophyte; Voucher: Nacoulma 120 (OUA-13484)

#### Rytigynia senegalensis

Blume

23462F52-0A9C-5BC0-913E-022F89D70DDF

##### Distribution

Sudanian

##### Notes

Life Form: phanerophyte; Voucher: Schmidt et al. (FR-0007390)

#### Sabicea venosa

Benth.

EB4D6B6A-517B-5FE5-BDB6-6BC8C36CC2B1

##### Distribution

Sudanian

##### Notes

Life Form: phanerophyte; Voucher: Schmidt et al. (FR-0007422)

#### Sarcocephalus latifolius

(Sm.) E.A.Bruce

F71B0BAA-5BC8-5183-A0B8-56BA302944EA

##### Distribution

Sudano-Zambesian

##### Notes

Life Form: phanerophyte; Voucher: Zizka (APPG-3871)

#### Spermacoce chaetocephala

DC.

E4020C00-6850-5DD2-843A-A678BA41385B

##### Distribution

Pluriregional African

##### Notes

Life Form: therophyte; Voucher: Nacoulma (APPG-70369)

#### Spermacoce filifolia

(Schumach. & Thonn.) J.-P.Lebrun & Stork

4AA8189B-E3B1-5554-A541-7AEA4E1091CF

##### Distribution

Pluriregional African

##### Notes

Life Form: therophyte; Voucher: Zwarg 8 (FR)

#### Spermacoce hepperana

Verdc.

F37FE482-8B31-5CF3-BEB0-AB9168BC9905

##### Distribution

Sudano-Zambesian

##### Notes

Life Form: therophyte; Voucher: Nacoulma 4493 (OUA-17073)

#### Spermacoce radiata

(DC.) Hiern

4C4CD8A8-AEBD-508E-BF24-02A17E17892B

##### Distribution

Sudano-Zambesian

##### Notes

Life Form: therophyte; Voucher: Nacoulma (APPG-70372)

#### Spermacoce stachydea

DC.

33442F0D-2338-5378-9756-B9E70AC9C32A

##### Distribution

Pantropical

##### Notes

Life Form: therophyte; Voucher: Zwarg 69 (FR)

#### Spermacoce verticillata

L.

304FA738-5337-508F-826F-6F0701191067

##### Distribution

Afrotropical (introduced)

##### Notes

Life Form: chamaephyte; Voucher: Nacoulma 253 (OUA-17219)

#### Rutaceae

4651903F-1817-5C72-8F23-6404E6B104B5

#### Zanthoxylum zanthoxyloides

(Lam.) Zepern. & Timler

4864A99F-6366-55CC-8EEC-2AB68F601066

##### Distribution

Sudanian

##### Notes

Life Form: phanerophyte

#### Salicaceae

7C50C511-DA7F-54E2-8302-CA18C9342EF9

#### Flacourtia indica

(Burm.f.) Merr.

C667E5E9-DF57-5F7F-8D5B-73E467287DD0

Flacourtia flavescens Willd.

##### Distribution

Pantropical

##### Notes

Life Form: phanerophyte; Voucher: Nacoulma 4538 (OUA-17107)

#### Oncoba spinosa

Forssk.

BA277731-C037-5182-9C6D-57CCC5B0756F

##### Distribution

Paleotropical

##### Notes

Life Form: phanerophyte

#### Salix chevalieri

Seemen

C039AAED-378E-5E48-B2FB-9450D3307089

##### Distribution

Afrotropical

##### Notes

Life Form: phanerophyte; Voucher: Schumann (FR-0083133); New species record for Burkina Faso.

#### Sapindaceae

333A7D6A-BBAE-5FCF-A36B-604D4CDB49C5

#### Allophylus africanus

P.Beauv.

264B6AE6-9552-5314-98E3-A7E5B7F41E92

##### Distribution

Afrotropical

##### Notes

Life Form: phanerophyte; Voucher: Schmidt et al. (FR-0007404)

#### Allophylus spicatus

(Poir.) Radlk.

F96A546B-60C8-5EE9-BA54-A661F3678D32

##### Distribution

Guineo-Congolian

##### Notes

Life Form: phanerophyte; Voucher: Nacoulma (OUA-17074)

#### Cardiospermum halicacabum

L.

60859E88-2498-53B1-B64F-6E9752648E74

##### Distribution

Pantropical

##### Notes

Life Form: phanerophyte; Voucher: Nacoulma 39 (OUA-13404)

#### Paullinia pinnata

L.

9BC1F0E9-AC6D-5D26-B0BE-194EB8BED81C

##### Distribution

Afro-American

##### Notes

Life Form: phanerophyte; Voucher: Schmidt et al. (FR-0007393)

#### Zanha golungensis

Hiern

479E686C-1655-56F7-964A-33916C6CB0F8

##### Distribution

Afrotropical

##### Notes

Life Form: phanerophyte; Voucher: Nacoulma 4566 (OUA-17129)

#### Sapotaceae

E64C6681-778C-5BB8-86F9-5F576A275BC1

#### Manilkara multinervis

(Baker) Dubard

EBF66431-839A-5574-891E-C5DA4B709127

##### Distribution

Pluriregional African

##### Notes

Life Form: phanerophyte; Voucher: Nacoulma 109 (OUA-13473)

#### Mimusops kummel

Bruce ex A.DC.

02ADB0CE-38A9-5818-A766-7ABC7F7EEAAE

##### Distribution

Sudanian

##### Notes

Life Form: phanerophyte

#### Vitellaria paradoxa

C.F.Gaertn.

99338910-7FD5-554F-AAF2-F1C5E4A803AE

##### Distribution

Sudanian

##### Notes

Life Form: phanerophyte; Voucher: Georg Zizka (APPG-3906)

#### Selaginellaceae

8777ADC0-3381-58F7-AB4C-6B6CBF48FAB6

#### Selaginella buchholzii

Hieron.

667324C4-A7EF-5D57-B462-EDB81331268B

##### Distribution

Guineo-Congolian

##### Notes

Life Form: therophyte; Voucher: Nacoulma 5289 (OUA-17775); New species record for Burkina Faso.

#### Simaroubaceae

1ECF9833-5189-580E-AC3A-2D31349CC810

#### Quassia undulata

(Guill. & Perr.) D.Dietr.

AC26CDEF-8AEE-5101-AABB-77D815D3A695

Hannoa undulata (Guill. & Perr.) Planch.

##### Distribution

Sudanian

##### Notes

Life Form: phanerophyte; Voucher: Nacoulma 100 (OUA-13464)

#### Solanaceae

DB5E6520-2619-5D99-AAEF-23404EC83E1F

#### Physalis lagascae

Roem. & Schult.

36A0FEC8-4C9A-5647-AB62-6BD88EFBB761

##### Distribution

Sudanian

##### Notes

Life Form: therophyte; Voucher: Schumann (FR-0083123)

#### Solanum incanum

L.

61E16CA3-61C4-5835-AA47-0A3F9AA26E91

##### Distribution

Paleotropical

##### Notes

Life Form: phanerophyte; Voucher: Schumann (FR-0083122)

#### Sphenocleaceae

932D4D72-D1F2-537C-9063-AA7F74820894

#### Sphenoclea zeylanica

Gaertn.

70FE0D8F-8A2D-52DB-9369-BB28E284C298

##### Distribution

Pantropical

##### Notes

Life Form: therophyte; Voucher: Nacoulma 210 (OUA-13573)

#### Thymelaeaceae

D02B18D7-C9F8-58CC-A4A3-33C5B5E53D0B

#### Gnidia kraussiana

Meisn.

591F74F2-D28B-50A4-9149-451C49440C48

##### Distribution

Sudanian

##### Notes

Life Form: chamaephyte; Voucher: Nacoulma 4490 (OUA-17076)

#### Synaptolepis retusa

H.Pearson

14A4E8E7-0CF4-549E-8EED-E6EB4C805850

##### Distribution

Sudanian

##### Notes

Life Form: chamaephyte; Voucher: Nacoulma (OUA-17124); New species record for Burkina Faso.

#### Urticaceae

E176E5EB-3183-556E-B3B8-CC6142A8414E

#### Pouzolzia guineensis

Benth.

2692717A-789E-5984-8665-9B83C971F285

##### Distribution

Pantropical

##### Notes

Life Form: phanerophyte; Voucher: Schmidt et al. (FR-0007631)

#### Violaceae

1E7542C3-BE04-5029-AC78-4EDAC2E152CD

#### Hybanthus enneaspermus

(L.) F.Muell.

5C5FA621-845B-56BB-B1ED-31F76912C24F

##### Distribution

Paleotropical

##### Notes

Life Form: therophyte

#### Vitaceae

082B5A81-F48A-5D38-99DA-72C9A3C019FA

#### Ampelocissus bombycina

(Baker) Planch.

9CF02EA0-B0E4-587B-B095-B05D59258D16

##### Distribution

Sudanian

##### Notes

Life Form: phanerophyte; Voucher: Nacoulma 4567 (OUA-17128); New species record for Burkina Faso.

#### Ampelocissus leonensis

(Hook.f.) Planch.

59086296-4E7A-5F79-80A8-669837550A52

##### Distribution

Sudanian

##### Notes

Life Form: phanerophyte

#### Ampelocissus multistriata

(Baker) Planch.

2DA49B82-C039-5A95-85B5-5221751DD590

Ampelocissus pentaphylla Gilg & M.Brandt

##### Distribution

Sudano-Zambesian

##### Notes

Life Form: phanerophyte; Voucher: Nacoulma 4617 (OUA-17208)

#### Cayratia gracilis

(Guill. & Perr.) Suess.

0EC9A04A-EB44-510B-87C6-88D82D62E6D4

##### Distribution

Afro-American

##### Notes

Life Form: phanerophyte

#### Cissus aralioides

(Welw. ex Baker) Planch.

160F9D47-A8EC-5F30-A257-829E7145EEF9

##### Distribution

Afrotropical

##### Notes

Life Form: phanerophyte; Voucher: Nacoulma 51 (OUA-13416)

#### Cissus cornifolia

(Baker) Planch.

62794964-527B-577F-8A44-4A98244D9A2C

##### Distribution

Sudanian

##### Notes

Life Form: chamaephyte

#### Cissus diffusiflora

(Baker) Planch.

0938D3C1-5E65-5538-AAE7-C52AEA3F6802

##### Distribution

Guineo-Congolian

##### Notes

Life Form: hemicryptophyte

#### Cissus palmatifida

(Baker) Planch.

EF7C4E54-D149-5C70-890F-8FD954D4A9DE

##### Distribution

Pluriregional African

##### Notes

Life Form: phanerophyte; Voucher: Nacoulma (APPG-69807)

#### Cissus populnea

Guill. & Perr.

BE5F12A3-E5B1-5DCD-9ADC-607B9578262D

##### Distribution

Sudanian

##### Notes

Life Form: phanerophyte; Voucher: Nacoulma (APPG-69810)

#### Cissus quadrangularis

L.

705623A1-4E05-5DEB-B6C9-EBE36D64F026

##### Distribution

Paleotropical

##### Notes

Life Form: phanerophyte; Voucher: Nacoulma (APPG-69812)

#### Cissus rufescens

Guill. & Perr.

B48F4BAE-913C-5618-986B-C142D59B9ED3

##### Distribution

Sudanian

##### Notes

Life Form: geophyte

#### Cyphostemma adenocaule

(Steud. ex A.Rich.) Desc. ex Wild & R.B.Drumm.

170F90BC-C253-54AF-ACAC-A8E2FFE338B2

##### Distribution

Sudanian

##### Notes

Life Form: chamaephyte; Voucher: Nacoulma (APPG-69920)

#### Cyphostemma flavicans

(Baker) Desc.

F97547BD-0319-5BF7-8A0C-B1613E67211D

##### Distribution

Sudanian

##### Notes

Life Form: geophyte; Voucher: Nacoulma (APPG-69805)

#### Xanthorrhoeaceae

07424144-9484-5D88-8F8A-CF3A853CFCD3

#### Aloe buettneri

A.Berger

D5E7C358-AA72-5E4C-AB84-30A883762213

##### Distribution

Sudanian

##### Notes

Life Form: geophyte

#### Ximeniaceae

A8CE3E98-76EC-57B9-B893-89A13B4DFC76

#### Ximenia americana

L.

EB8A0FA6-0F9D-50C8-A13A-288826484F32

##### Distribution

Pantropical

##### Notes

Life Form: phanerophyte; Voucher: Hahn (APPG-2867)

#### Zingiberaceae

70073AB2-0CA1-512B-994B-65F07C4BAAEE

#### Siphonochilus aethiopicus

(Schweinf.) B.L.Burtt

B1908582-8666-5128-BD44-BA99CEBE06B0

##### Distribution

Sudano-Zambesian

##### Notes

Life Form: geophyte; Voucher: Nacoulma (APPG-70133)

#### Zygophyllaceae

4F813C95-0A7E-545B-97BC-D0597A467B16

#### Balanites aegyptiaca

(L.) Delile

A4CB6E2B-F636-51D7-B740-5FCF8EA3A77F

##### Distribution

Sudanian

##### Notes

Life Form: phanerophyte

## Analysis

### Composition of the local flora

The 721 species found in the study area belong to 385 genera and 102 plant families, the families with most species being Fabaceae (126 spp.), Poaceae (102 spp.) and Cyperaceae (44 spp.) (Fig. 3). The genera with most species in the study area are *Indigofera* (20 spp.), *Crotalaria* (17 spp.) and *Acacia* (13 spp.), all belonging to Fabaceae.

**Figure 4.**
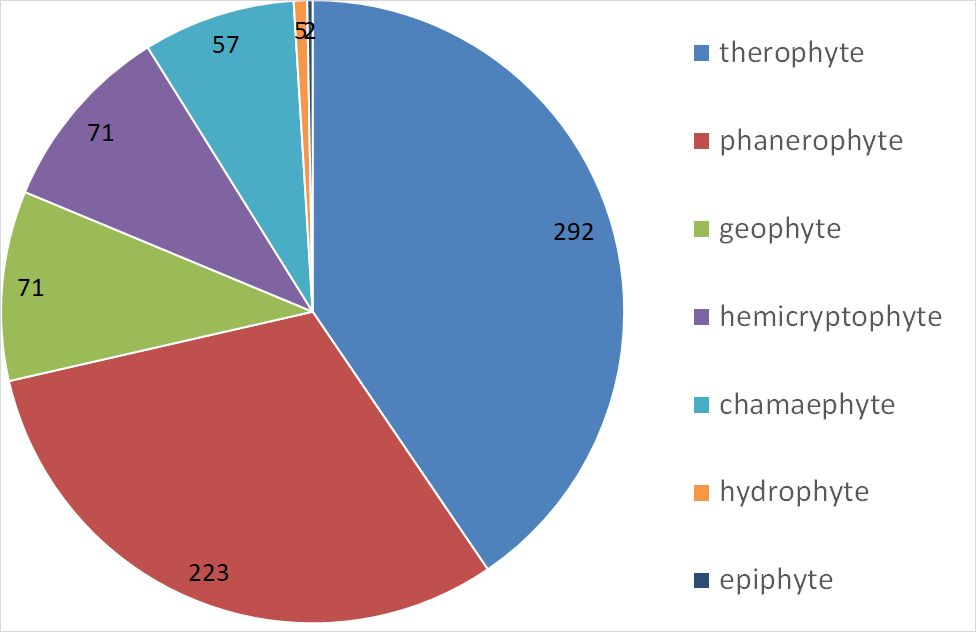
Life form spectrum of the flora of the W National Park.

Therophytes and phanerophytes are the most prominent life forms, followed by hemicryptophytes, geophytes and chamaephytes (Fig. 4). Only very few hydrophytes and epiphytes are present.

**Figure 5.**
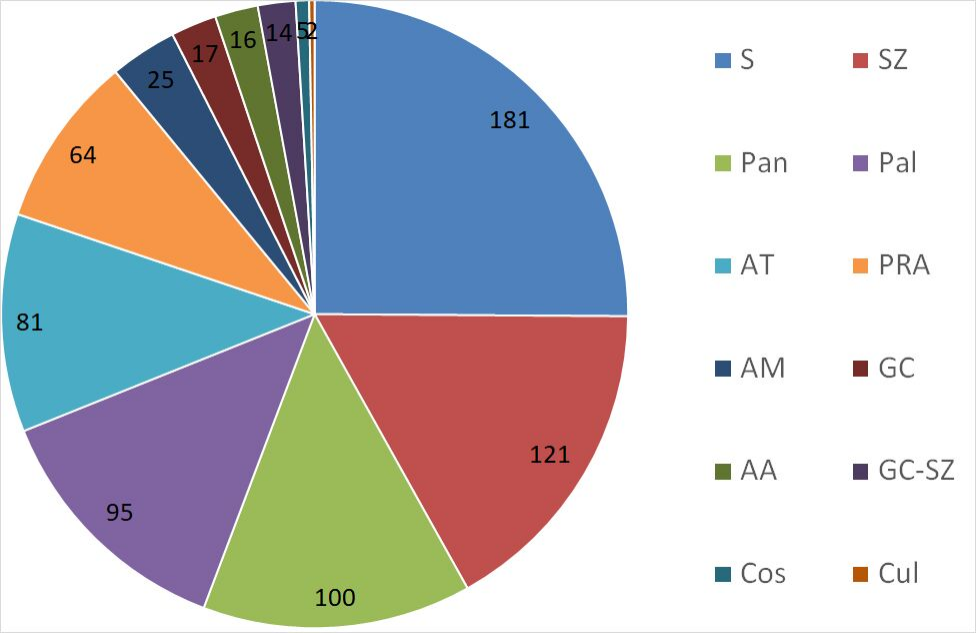
Distribution types of the flora of W National Park: sudanian (S), sudano-zambesian (SZ), pantropical (Pan), paleotropical (Pal), afrotropical (AT), pruriregional African (PRA), afro-malagasy (AM), guineo-congolian (GC), afro-american (AA), guineo-congolian/sudano-zambesian (GC-SZ), cosmopolitan (Cos), of cultivated origin (Cul).

The best represented distribution types in the flora are specific to the vegetation zone, either Sudanian or Sudano-Zambesian (Fig. 5). Species with wider distributions in the tropics (pantropical, paleotropical) or within continental Africa (afrotropical, pluriregional African) follow. The remaining distribution types have smaller shares and comprise either distributions extending to other continents or Madagascar or belong to the neighbouring zones under higher rainfall regimes, typically extending into the Sudan along gallery forests of the larger watercourses.

### New species records for Burkina Faso

A number of species have been found in the study area that have not been documented in the vascular plant catalogue of Burkina Faso (Thiombiano et al. 2012), nor in the supplement by Schmidt (2018); some of these are shown in Fig. 2. These 19 species are *Aponogeton vallisnerioides* (Aponogetonaceae), *Drimiopsis barteri* (Asparagaceae), *Eleocharis naumanniana* (Cyperaceae), *Jatropha atacorensis*, *Tragia laminularis* (Euphorbiaceae), *Aeschynomene americana*, *Alysicarpus vaginalis*, *Crotalaria lachnophora*, *Desmodium ramosissimum*, *Indigofera garckeana*, *Vigna nigritia* (Fabaceae), *Gladiolus unguiculatus* (Iridaceae), *Ophioglossum thomasii* (Ophioglossaceae), *Chloris gayana*, *Loxodera ledermannii* (Poaceae), *Salix chevalieri* (Salicaceae), *Selaginella buchholzii* (Selaginellaceae), *Synaptolepis retusa* (Thymelaeaceae) and *Ampelocissus bombycina* (Vitaceae). The number of vascular plants known for Burkina Faso therefore increases to 2099 species.

### Red Lists

Unlike for neighbouring Benin (Neuenschwander et al. 2011), there is no country-specific Red List for Burkina Faso yet. Amongst the 14 species listed for Burkina Faso on the global IUCN Red List (IUCN 2020) in the categories endangered (EN), near threatened (NT) or vulnerable (VU), the following occur in our study area: *Pterocarpus erinaceus* (EN), *Afzelia africana* (VU) and *Khaya senegalensis* (VU).

### Introduced species

Amongst the species occurring in the study area, 17 species have been identified as being introduced: *Acanthospermum hispidum, Ageratum conyzoides, Azadirachta indica, Bidens pilosa, Cassia obtusifolia, Chrysanthellum indicum, Desmodium adscendens, Hyptis spicigera, H. suaveolens, Indigofera microcarpa, Ludwigia erecta, Martynia annua, Passiflora foetida, Plumbago zeylanica, Scoparia dulcis, Spermacoce verticillata* and *Tridax procumbens*. The families best represented in this group are Asteraceae (5 spp.) and Fabaceae (3 spp.).

## Discussion

With 721 documented species, the Burkina Faso part of the W National Park is, so far, the most species-rich nature reserve in Burkina Faso. Neither the protected areas of the same Sudano-sahelian climatic zone nor those in the Sudanian climatic zone have an equally-high diversity. In the Sudano-sahelian zone, the reserve of Pama and Arly National Park sustain 450 and 490 species, respectively (Mbayngone et al. 2008, Ouédraogo et al. 2011), whereas the classified forests of Kou and Niangoloko, as well as the classified forest and partial faunal reserve of Comoé-Léraba in the Sudanian zone, have 353, 275 and 521 species, respectively (Guinko and Thiombiano 2005, Ouôba 2006, Gnoumou et al. 2015).

Several factors might explain this: to a large extent, the high plant diversity can be assigned to habitat diversity and a moderate degree of disturbance, but also to a relatively large area within an even larger complex of neighbouring protected areas. In Burkina Faso, the study sites are known for the diversity of their natural habitats, i.e. the Gobnangou and Atakora mountains, wetlands and gallery forests, termite mounds as well as anthropogenic relics of the past. Anthropogenic relics are still visible with metallurgical sites and baobab vegetation types, being closely linked to human settlements as shown by Duvall (2007).

Thorough spatial, ecological and seasonal sampling have certainly contributed to a good documentation of the area, building on experiences from similar studies in protected areas and focussed collection activities for the documentation of the flora of Burkina Faso (van der Burgt et al. 2010).

Finally, based on different scenarios, recent investigations (Schmidt et al. 2017) identified the southeast of Burkina Faso, around the W National Park, as one of the two hotspots of plant diversity of the country. Our present results confirm and strengthen these scenarios. There are no species endemic to W National Park and Isoetes jaegeri, the only plant still recognised as endemic to Burkina Faso does not occur here, being known exclusively from the sandstone massif in the southwest of the country. Including the 19 newly-documented species, the W National Park harbours more than one third of the plant diversity known for Burkina Faso, making it an important area for plant conservation in a landscape setting where biodiversity outside of protected areas is increasingly threatened by intensified agriculture (Zoungrana et al. 2018). At the country level, Da et al. (2018) evaluated the percentage of modelled plant species distributions covered by protected areas and came up with a figure of 77% for Burkina Faso. Our results confirm the importance of these reserves for plant conservation with primary biodiversity data.

The composition of the flora by families, life forms and distribution types follows the same patterns as in other protected areas of the WAP complex (Assédé et al. 2012, Mbayngone et al. 2008, Ouédraogo et al. 2011). The dominance of Fabaceae and Poaceae may be explained by their respective innovations in N-fixation and C_4_-photosynthesis, making them well-adapted to savanna environments. Therophytes and phanerophytes are the dominant life forms here and in the wider area of the West African savanna belt, with therophytes being more important in the drier north and phanerophytes in the wetter south (Schmidt et al. 2005). The large proportion of widespread species in West African savanna areas like the W National Park may be explained by the dominance of the savanna vegetation in a vast continuous band throughout West Africa in combination with the lack of effective barriers, such as higher mountains which separate savanna areas in other parts of Africa (White 1983).

Only few species from Burkina Faso have been Red-listed by IUCN, the three species in the W National Park all being sought-after timber species. The IUCN Red List is a good instrument to target species for conservation measures.However, only few species of the West African Flora have been evaluated, for Burkina Faso less than 20% (400 out of 2099 spp., IUCN 2020). Continued efforts will be necessary to get a better idea on threats and necessary conservation measures and a prerequisite are scientifically reliable assessments, accessible to the community and representing important parts of the region.

The number of 17 introduced plant species (2.3%) is rather low when compared to the 116 introduced species (5.5%) on the country scale (Zizka et al. 2015). It shows the success of these neophytes in protected areas under limited anthropogenic influence and may stimulate research on introduction routes and more in-depth studies on neobiota in Burkina Faso in general.

## Figures and Tables

**Figure 2a.**
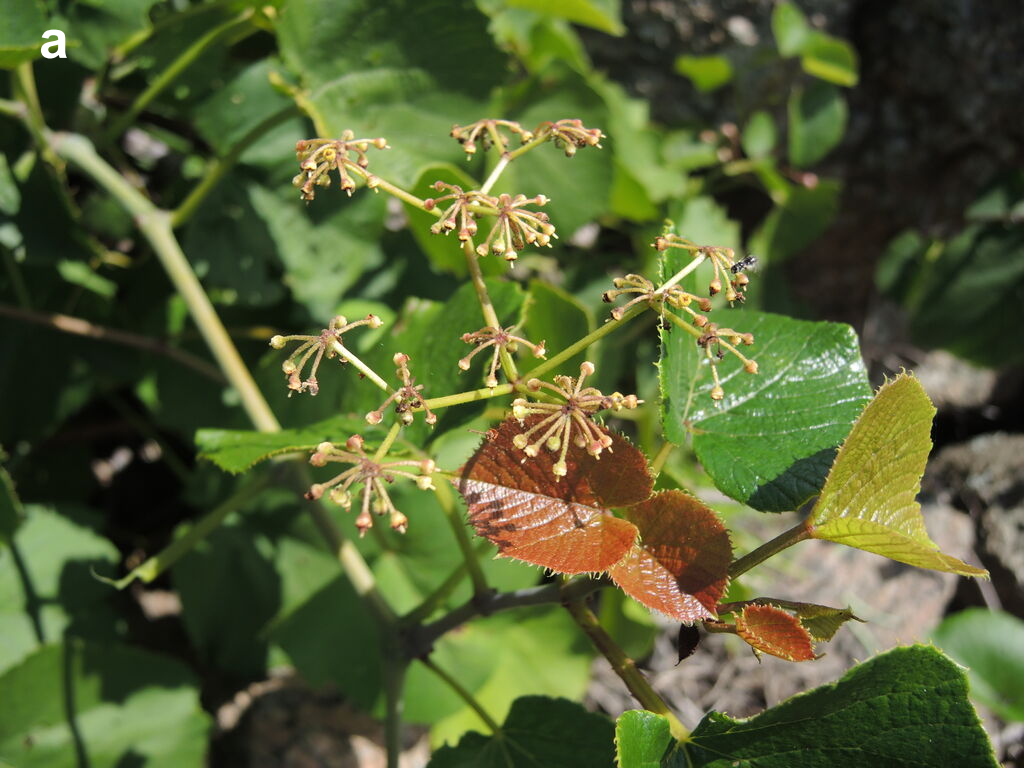
*Ampelocissus bombycina* (Photo by Meike Piepenbring)

**Figure 2b.**
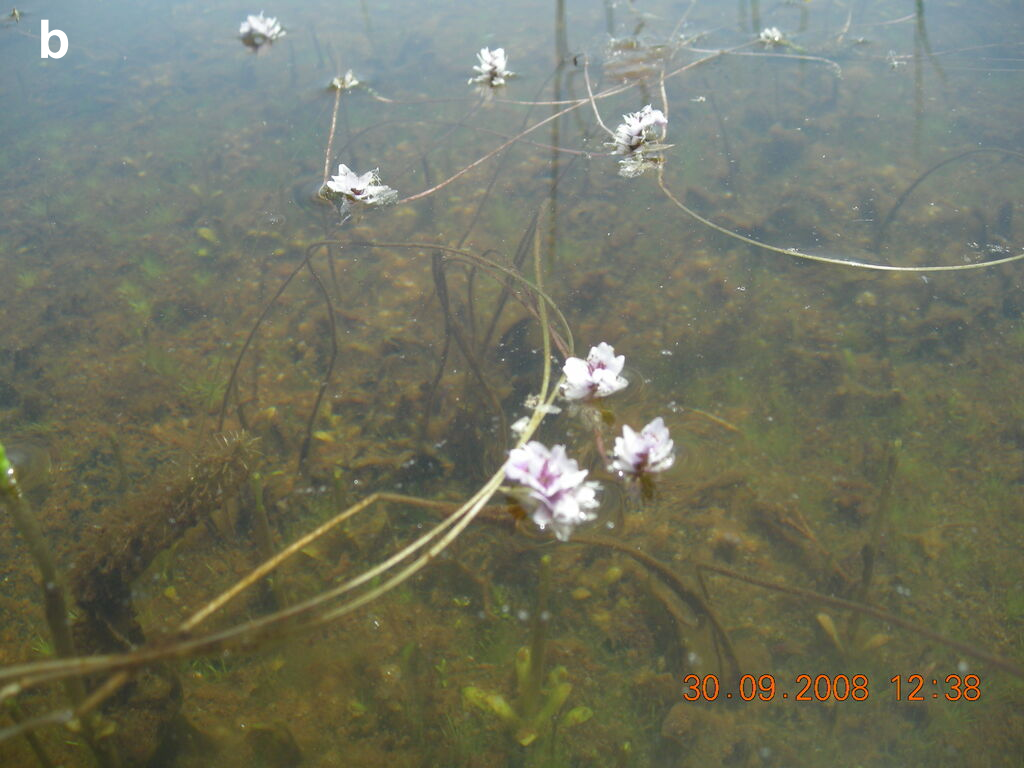
*Aponogeton vallisnerioides*

**Figure 2c.**
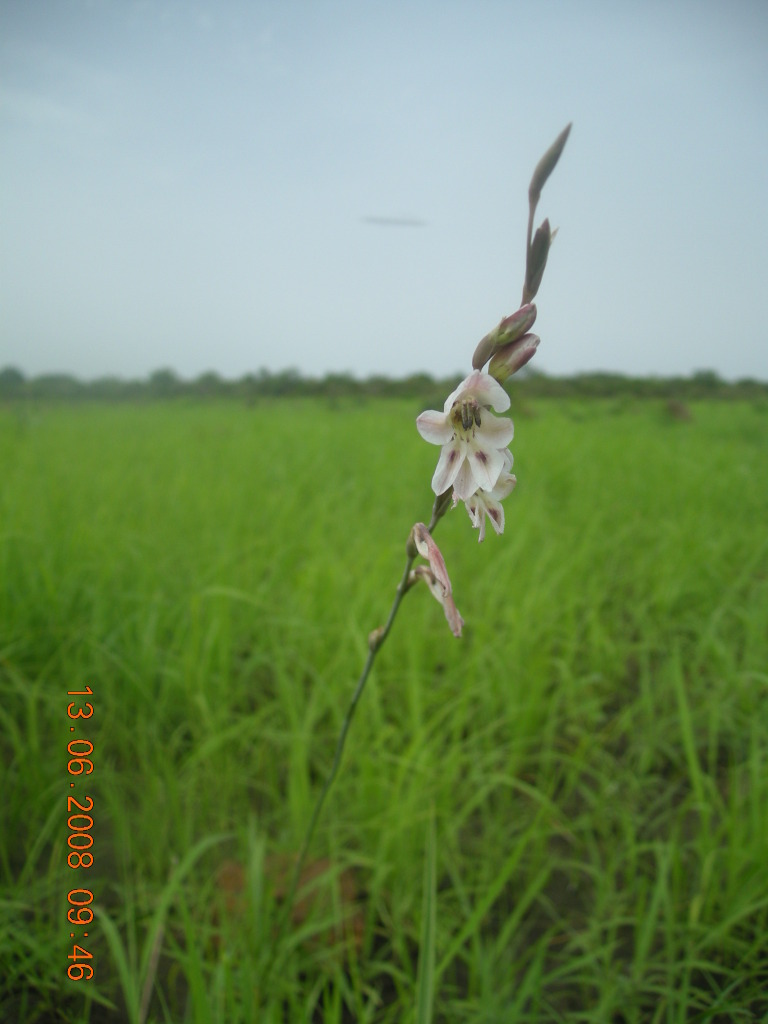
*Gladiolus unguiculatus*

**Figure 2d.**
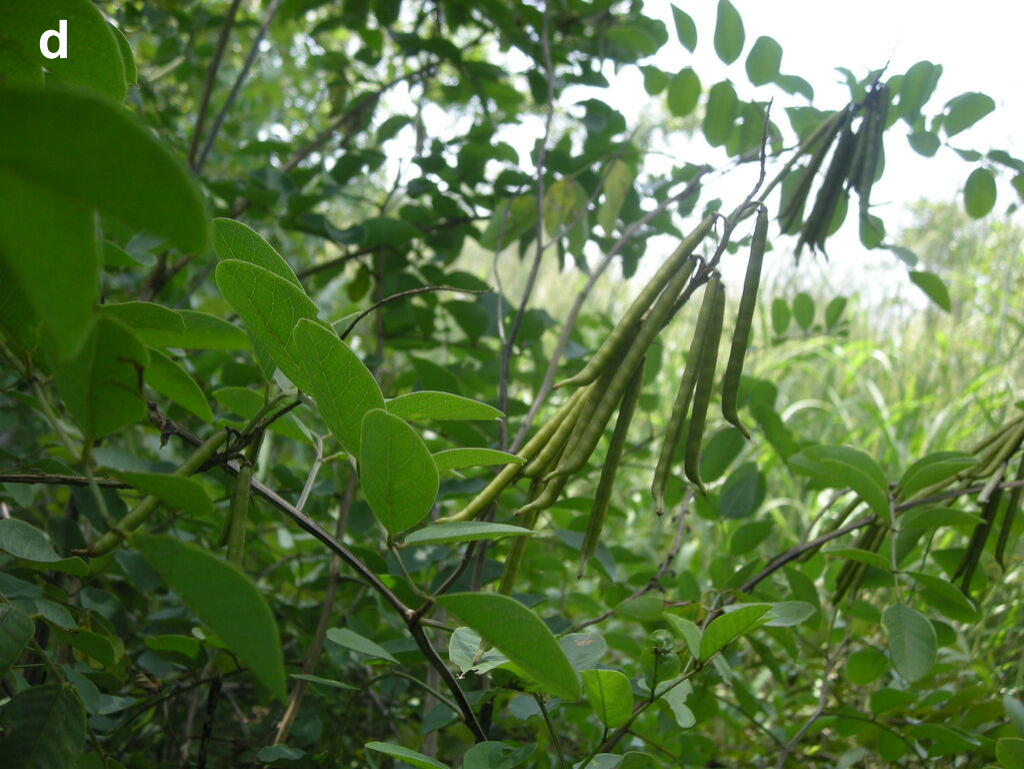
*Indigofera garckeana*

**Figure 2e.**
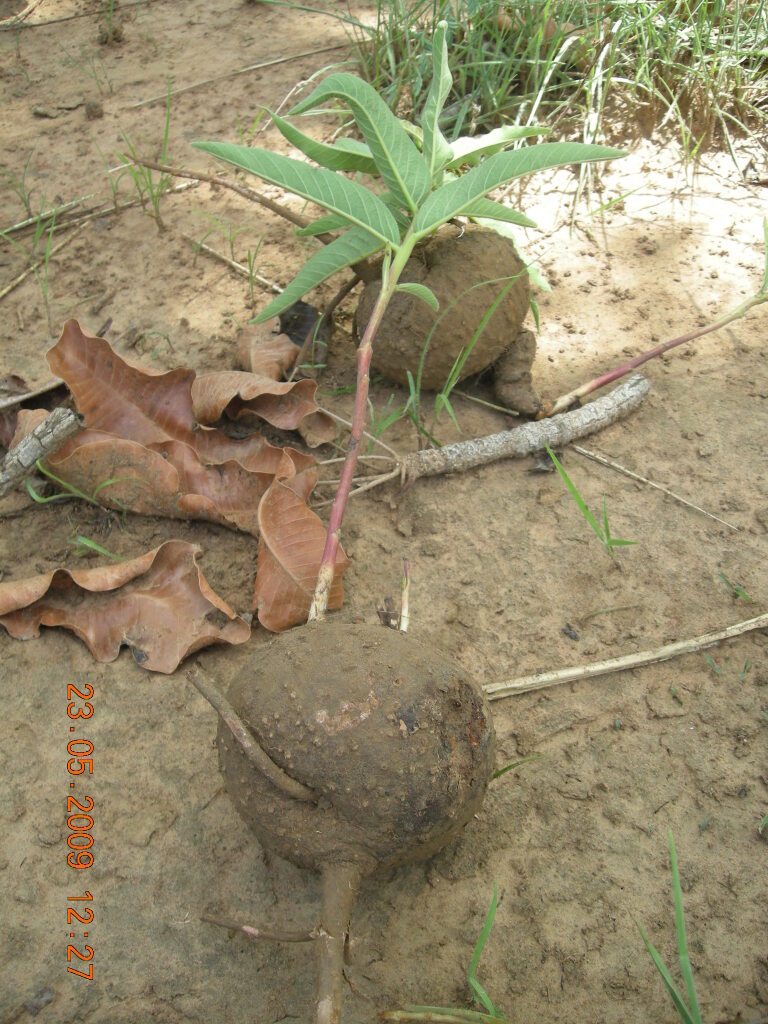
*Jatropha atacorensis*

**Figure 2f.**
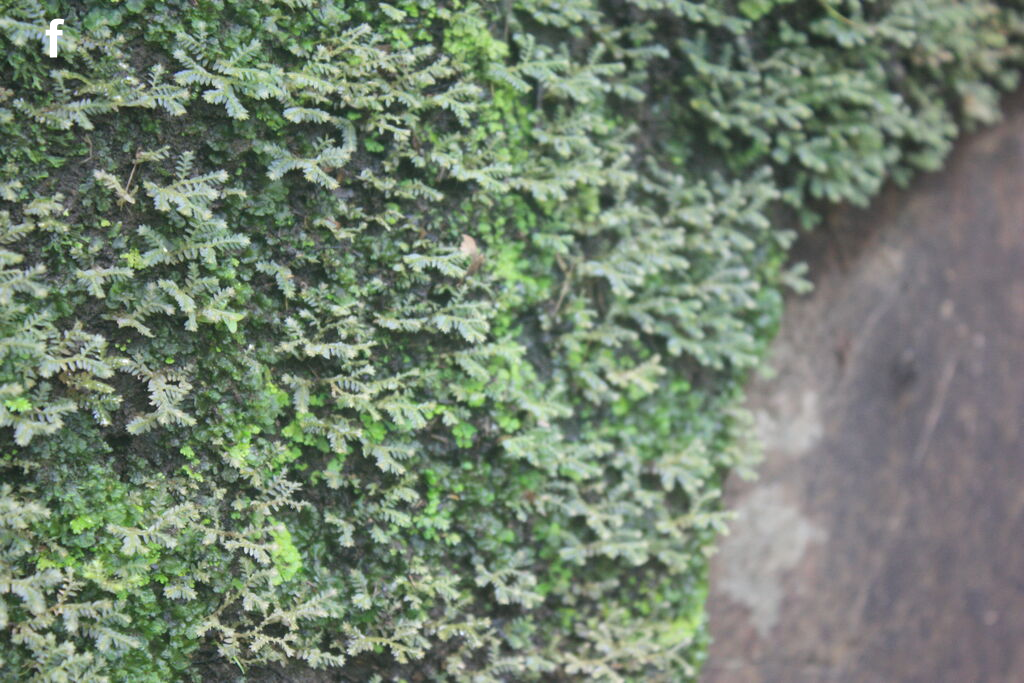
*Selaginella buchholzii*

## References

[B5792336] Thiombiano Adjima, Kampmann Dorothea, Adouabou Basile, Belemsobgo Urbain, Nana Sonemanegré, Kafando Pierre (2010). Fauna. Biodiversity Atlas of West Africa - Burkina Faso.

[B5700373] Akoegninou A., van der Burg W. J., van der Maesen L. J.G. (2006). Flore analytique du Bénin.

[B5700382] Arbonnier Michel (2000). Arbres, arbustes et lianes des zones sèches d’Afrique de l’Ouest.

[B5700391] Assédé Emeline P. S., Adomou Aristide C., Sinsin Brice (2012). Magnoliophyta, Biosphere Reserve of Pendjari, Atacora Province, Benin. Check List.

[B5700628] Balança G., Cornélis D., Wilson R. (2007). Les oiseaux du Complexe WAP.

[B5700657] Clerici Nicola, Bodini Antonio, Eva Hugh, Grégoire Jean-Marie, Dulieu Dominique, Paolini Carlo (2007). Increased isolation of two biosphere reserves and surrounding protected areas (WAP ecological complex, West Africa). Journal for Nature Conservation.

[B5885275] Sahara Conseil Scientifique pour l'Afrique au Sud du (1956). Specialist Meeting on Phytogeography, Yangambi, 28th July-8th August, 1956.

[B5792292] Da Sié Sylvestre, García Márquez Jaime Ricardo, Sommer Jan Henning, Thiombiano Adjima, Zizka Georg, Dressler Stefan, Schmidt Marco, Chatelain Cyrille, Barthlott Wilhelm (2018). Plant biodiversity patterns along a climatic gradient and across protected areas in West Africa. African Journal of Ecology.

[B5700669] Dressler Stefan, Schmidt Marco, Zizka Georg (2014). Introducing African plants — A photo guide — An interactive photo data-base and rapid identification tool for Continental Africa. Taxon.

[B5700679] Duvall Chris S. (2007). Human settlement and baobab distribution in south-western Mali. Journal of Biogeography.

[B5866730] Erpenbach Arne, Bernhardt-Römermann Markus, Wittig Rüdiger, Thiombiano Adjima, Hahn Karen (2012). The influence of termite-induced heterogeneity on savanna vegetation along a climatic gradient in West Africa. Journal of Tropical Ecology.

[B5700980] Fontès J., Guinko Sita (1995). Carte de la végétation et de l’occupation du sol au Burkina Faso. Notice explicative.

[B5700716] Gnoumou Assan, Ouedraogo Oumarou, Schmidt Marco, Thiombiano Adjima (2015). Floristic diversity of classified forest and partial faunal reserve of Comoé-Léraba, southwest Burkina Faso. Check List.

[B5700726] Guinko Sita, Thiombiano Adjima (2005). Florule de la Forêt Classée du Kou (Burkina Faso).

[B5885422] Hahn-Hadjali Karen (1998). Pflanzensoziologische Studien der sudanesischen Savannen im Südosten Burkina Fasos (Westafrika). Etudes sur la flore et la végétation du Burkina Faso et des pays avoisinants.

[B5700735] Hutchinson J., Dalziel J. M. (1972). Flora of West Tropical Africa.

[B5746777] IUCN The IUCN Red List of Threatened Species. Version 2020-1. www.iucnredlist.org.

[B5700762] Konrad T. (2015). Governance of protected areas in West Africa. The case of the W-Arly-Pendjari (WAP) complex in Benin and Burkina Faso.

[B5700771] Lamarque F. (2004). Les grands mammifères du complexe WAP.

[B5700780] Mahamane Ali (2005). Etudes floristique, phytosociologique et phytogéographique de la végétation du Parc Régional du W du Niger.

[B5700790] Mbayngone Elisée, Schmidt Marco, Hahn-Hadjali Karen, Thiombiano Adjima, Guinko Sita (2008). Magnoliophyta of the partial faunal reserve of Pama, Burkina Faso. Check List.

[B5746786] Miller Daniel C, Minn Michael, Sinsin Brice (2015). The importance of national political context to the impacts of international conservation aid: evidence from the W National Parks of Benin and Niger. Environmental Research Letters.

[B5794363] Thiombiano Adjima, Kampmann Dorothea, Nacoulma Blandine Marie Ivette, Ouédraogo Oumarou (2010). W National Park of Burkina Faso: a park with enormous potential. Biodiversity Atlas of West Africa - Burkina Faso.

[B5700810] Nacoulma Blandine Marie Ivette, Schumann Katharina, Traoré Salifou, Bernhardt-Römermann Markus, Hahn Karen, Wittig Rüdiger, Thiombiano Adjima (2011). Impacts of land-use on West African savanna vegetation: a comparison between protected and communal area in Burkina Faso. Biodiversity and Conservation.

[B5700801] Nacoulma Blandine Marie Ivette (2012). Dynamique et stratégies de conservation de la végétation et de la phytodiversité du complexe écologique du Parc National du W du Burkina Faso..

[B5746768] Neuenschwander Peter, Sinsin Brice, Goergen Georg (2011). Protection de la Nature en Afrique de l’Ouest: Une Liste Rouge pour le Bénin.

[B5885432] Ouédraogo Amadé (2006). Diversité et dynamique de la végétation ligneuse de la partie orientale du Burkina Faso.

[B5700823] Ouédraogo Oumarou, Schmidt Marco, Thiombiano Adjima, Hahn Karen, Guinko Sita, Zizka Georg (2011). Magnoliophyta, Arly National Park, Tapoa, Burkina Faso. Check List.

[B5700835] Ouôba Paulin (2006). Flore et végétation de la forêt classée de Niangoloko, Sud-ouest du Burkina Faso.

[B5700853] Perez-Vera F. (2003). Les orchidées de Côte d’Ivoire.

[B5700844] PNUD (2004). Renforcer l’efficacité et catalyser la durabilité du système des aires protégées du W-Arly-Pendjari (WAP).

[B5700862] Poilecot Pierre (1995). Les Poaceae de Côte d’Ivoire.

[B5700871] Poilecot Pierre (1999). Les Poaceae du Niger.

[B5792282] Schmidt Marco, Kreft Holger, Thiombiano Adjima, Zizka Georg (2005). Herbarium collections and field data-based plant diversity maps for Burkina Faso. Diversity & Distributions.

[B5700953] van der Burgt Xander, van der Maesen Jos, Onana J. M., Schmidt Marco, Thiombiano Adjima, Ouédraogo Amadé, Dressler Stefan, Hahn-Hadjali Karen, Zizka Georg (2010). Assessment of the flora of Burkina Faso. Systematics and conservation of African plants.

[B5700942] Schmidt Marco, Assédé Emeline, Oebel Horst, Fahr Jakob, Sinsin Brice (2016). Biota of the WAP complex – starting a citizen science project for West Africa’s largest complex of protected areas. Flora et Vegetatio Sudano-Sambesica.

[B5700916] Schmidt Marco, Zizka Alexander, Traoré Salifou, Ataholo Mandingo, Chatelain Cyrille, Daget Philippe, Dressler Stefan, Hahn Karen, Kirchmair Ivana, Krohmer Julia, Mbayngone Elisée, Müller Jonas V., Nacoulma Blandine, Ouédraogo Amadé, Ouédraogo Oumarou, Sambaré Oumarou, Schumann Katharina, Wieringa Jan J., Zizka Georg, Thiombiano Adjima (2017). Diversity, distribution and preliminary conservation status of the flora of Burkina Faso. Phytotaxa.

[B5700880] Schmidt Marco (2018). New species records for the flora of Burkina Faso. Flora et Vegetatio Sudano-Sambesica.

[B5883095] Schmidt Marco, Ouédaogo Amadé, Thiombiano Adjima, Wong Lian Jenna, Pagad Shyama (2020). Global Register of Introduced and Invasive Species - Burkina Faso.

[B5746878] Schulte to Bühne Henrike, Wegmann Martin, Durant Sarah M., Ransom Chris, de Ornellas Paul, Grange Sophie, Beatty Hope, Pettorelli Nathalie (2017). Protection status and national socio-economic context shape land conversion in and around a key transboundary protected area complex in West Africa. Remote Sensing in Ecology and Conservation.

[B5866749] Schumann Katharina, Nacoulma Blandine M. I., Hahn Karen, Traoré Salifou, Thiombiano Adjima, Bachmann Yvonne (2016). Modeling the distributions of useful woody species in eastern Burkina Faso. Journal of Arid Environments.

[B5700969] Thiombiano Adjima, Schmidt Marco, Dressler Stefan, Ouédraogo Amadé, Hahn Karen, Zizka Georg (2012). Catalogue des plantes vasculaires du Burkina Faso.

[B5792327] Committee UNESCO World Heritage Decision : 41 COM 8B.3 W-Arly-Pendjari Complex (Benin, Burkina Faso, Niger). http://whc.unesco.org/en/decisions/6867.

[B5792391] White Frank (1983). The Vegetation of Africa, a descriptive memoir to accompany the UNESCO/AETFAT/UNSO vegetation map of Africa.

[B5883118] Zizka Alexander, Thiombiano Adjima, Dressler Stefan, Nacoulma Blandine M. I., Ouédraogo Amadé, Ouédraogo Issaka, Ouédraogo Oumarou, Zizka Georg, Hahn Karen, Schmidt Marco (2015). The vascular plant diversity of Burkina Faso (West Africa) — A quantitative analysis and implications for conservation. Candollea.

[B5746867] Zoungrana Benewinde J-B., Conrad Christopher, Thiel Michael, Amekudzi Leonard K., Da Evariste Dapola (2018). MODIS NDVI trends and fractional land cover change for improved assessments of vegetation degradation in Burkina Faso, West Africa. Journal of Arid Environments.

[B5866761] Zwarg Alexandra, Schmidt Marco, Janßen Thomas, Hahn Karen, Zizka Georg (2012). Plant diversity, functional traits and soil conditions of grass savannas on lateritic crusts (bowé) in south eastern Burkina Faso. Flora et Vegetatio Sudano-Sambesica.

